# TRIM47-facilitated PLK1 stabilization promotes the proliferation of liver cancer cells

**DOI:** 10.1007/s13402-025-01130-0

**Published:** 2025-12-29

**Authors:** Tao Xie, Xiaoqi Fan, Tian Yu, Qing Zhu, Qi Zhang, Na Li, Tao Wang, Yisong Qian, Keyu Deng, Hongbo Xin, Yong Li, Xuan Huang

**Affiliations:** 1 The MOE Basic Research and Innovation Center for the Targeted Therapeutics of Solid Tumors, Jiangxi Province Key Laboratory of Bioengineering Drugs, Institute of Translational Medicine, Jiangxi Medical College, Nanchang University, Nanchang, China; 2 School of Basic Medical Sciences, Institute of Biomedical Innovation, The First Affiliated Hospital, Jiangxi Medical College, Nanchang University, Nanchang, China; 3 School of Future Technology, Nanchang University, Nanchang, China; 4 HuanKui Academy, Jiangxi Medical College, Nanchang University, Nanchang, China; 5 Department of Anesthesiology, The First Affiliated Hospital, Jiangxi Medical College, Nanchang University, Nanchang, China

**Keywords:** TRIM47, Liver cancer, Cell proliferation, PLK1, Ubiquitination

## Abstract

**Background:**

Liver cancer (LC) remains a leading cause of cancer-related mortality worldwide, with limited therapeutic options for advanced-stage disease. While tripartite motif-containing 47 (TRIM47), a typical E3 ubiquitin ligase, has been implicated in cancer progression, its precise role in LC pathogenesis remains unclear.

**Methods:**

TRIM47 expression in hepatocellular carcinoma (HCC) tissues was analyzed through public databases. Functional assays, including TRIM47 knockdown and overexpression experiments, were performed to investigate its impact on cell proliferation, apoptosis and the cell cycle both in vitro and in vivo. The investigation was conducted using a combination of methodologies, including yeast-two hybrid screening, ubiquitination assays, and signaling pathway analyses, to elucidate the underlying mechanisms. In addition, rescue assays were performed to validate the effect of TRIM47-PLK1 axis on LC growth.

**Results:**

TRIM47 was significantly elevated in HCC tissues and positively correlated with advanced clinical stage and poor overall survival. Functionally, TRIM47 knockdown suppressed LC cell growth, while its overexpression promoted tumor proliferation. Furthermore, TRIM47 facilitates cell cycle progression by alleviating G2/M phase arrest and inhibits apoptosis in LC cells. Mechanistically, TRIM47 catalyzes K63-linked ubiquitination of polo-like kinase 1 (PLK1), leading to its stabilization and subsequent activation of the NF-κB and MAPK pathways. Clinical validation confirmed a significant positive correlation between TRIM47 and PLK1 expression in human HCC specimens. Importantly, pharmacological inhibition of PLK1 effectively abrogated TRIM47-driven tumor growth in xenograft models.

**Conclusion:**

This study identifies TRIM47 as a critical regulator of LC progression through PLK1 stabilization and proposes the TRIM47-PLK1 axis as a potential therapeutic target for LC treatment.

**Supplementary Information:**

The online version contains supplementary material available at 10.1007/s13402-025-01130-0.

## Introduction

Liver cancer (LC) is a highly malignant tumor that is prone to recurrence and metastasis and has a poor prognosis [1, 2]. Currently, primary carcinoma of the liver (PCL) ranks as the sixth most commonly diagnosed cancer and the third leading cause of cancer-related death worldwide. The main pathological subtypes include hepatocellular carcinoma (HCC), intrahepatic cholangiocarcinoma (ICC), and combined hepatocellular-cholangiocarcinoma (cHCC-CCA), among which HCC represents the predominant form [3–5]. Orthotopic transplantation and surgical resection remain the mainstays of treatment for LC [6]. However, due to the lack of obvious symptoms in the early stages, most patients are diagnosed at intermediate or advanced stages, thereby missing the window for surgical intervention. Sorafenib and Lenvatinib are the first-line regimens recommended by the current guidelines for advanced LC, but their efficacy is limited, extending the survival period by only approximately three months [7, 8]. Although immunotherapy has shown remarkable advances in recent years, the objective response rate to single-agent PD-1/PD-L1 inhibitors in advanced LC is merely 15%-20% [9, 10]. Given these therapeutic challenges, there is an urgent need to explore the underlying molecular mechanisms driving LC progression and to identify novel targets for more effective treatment strategies.

Tripartite motif (TRIM) proteins are a large family of E3 ubiquitin ligases that play crucial roles in various cellular processes, including the immune response [11], antiviral defense [12] and carcinogenesis [13–15]. The TRIM family consists of more than 70 members in humans and is characterized by the presence of a tripartite motif consisting of a RING domain, one or two B-box domains, and a coiled-coil region [16]. The RING domain confers E3 ubiquitin ligase activity on TRIM proteins, allowing them to transfer ubiquitin moieties to specific target proteins, leading to their degradation or modification [17]. As a typical E3 ligase of the TRIM family, TRIM47 is emerging as an important player in multiple cellular processes, has attracted increasing attention in recent years, and has been reported to be involved in apoptosis and inflammation in various diseases [18]. Subsequent studies revealed that TRIM47 is widely expressed in various tissues and has a broad range of functions. In cancer research, TRIM47 has been found to be upregulated in some cancers, such as ovarian cancer, glioma angiogenesis and breast cancer [19–21]. Nevertheless, the functional significance and underlying mechanisms of TRIM47 in LC remain poorly understood.

Polo-like kinases (PLKs) are a group of conserved serine/threonine protein kinases with five known members: PLK1 to PLK5 [22]. Structurally, PLK1-3 and PLK4 are highly homologous, whereas PLK5 lacks a C-terminal kinase domain. Among them, PLK1 is the most widely studied protein, which plays a key role in cell cycle control, apoptosis and tumor development [12, 23]. For example, PLK1 is markedly expressed in a multitude of malignant neoplasms, particularly in LC [24], and has the capacity to facilitate tumor development through classical pathways such as the PI3K-AKT, NF-κB, and MAPK pathways [25, 26]. Evidence that PLK1 inhibitors (e.g., volasertib) can reduce tumor cell proliferation and migration, induce apoptosis, and sensitize cells to chemotherapy highlights the promise of PLK1 as an anticancer drug target [27, 28]. However, although several PLK1 inhibitors have entered phase III clinical trials, none have yet achieved significant success in the clinic [29]. Thus, a deeper understanding of the regulatory mechanisms of PLK1, including its post-translational modifications, is warranted.

In this study, we identified TRIM47 as significantly upregulated in HCC clinical specimens and cell lines of LC. Functional assays demonstrated that TRIM47 knockdown effectively suppresses cell proliferation and progression of LC both in vitro and in vivo. Mechanistically, we revealed that TRIM47 exerts its oncogenic effects by facilitating K63-linked ubiquitination and stabilization of PLK1, leading to activation of the NF-κB and MAPK signaling pathways. This molecular cascade ultimately leads to cell cycle shortening and accelerated proliferation in LC. Our findings establish TRIM47 as a bona fide oncogene and propose that therapeutic targeting of the TRIM47/PLK1 axis may represent a promising strategy for LC treatment.

## Results

### TRIM47 is upregulated in human HCC tissues

To assess the relevance of TRIM47 in hepatocellular carcinoma (HCC), we first analyzed its expression using public proteomic and transcriptomic databases. Analysis of the Clinical Proteomic Tumor Analysis Consortium (CPTAC) database revealed that the protein expression levels of TRIM47 were significantly elevated in primary tumor tissues (*n* = 165) compared to normal liver tissues (*n* = 165) (Figure S1A). Consistent with this, immunohistochemistry (IHC) data from the Human Protein Atlas (HPA014933) demonstrated stronger TRIM47 staining in HCC specimens relative to normal controls (Figure S1B). Transcriptomic data from The Cancer Genome Atlas (TCGA) database further confirmed that the mRNA levels of TRIM47 were significantly higher in liver cancer tissues (*n* = 371) than in normal liver tissues (*n* = 50) (Figure S1C). Notably, TRIM47 mRNA levels positively correlated with advanced tumor stage (Figure S1D) and lymph node metastasis (Figure S1E). Moreover, Kaplan-Meier (K-M) survival analysis indicated that high TRIM47 expression was associated with shorter overall survival in HCC patients (Figure S1F).

### TRIM47 promotes the proliferation of LC cells

**Fig. 1:**
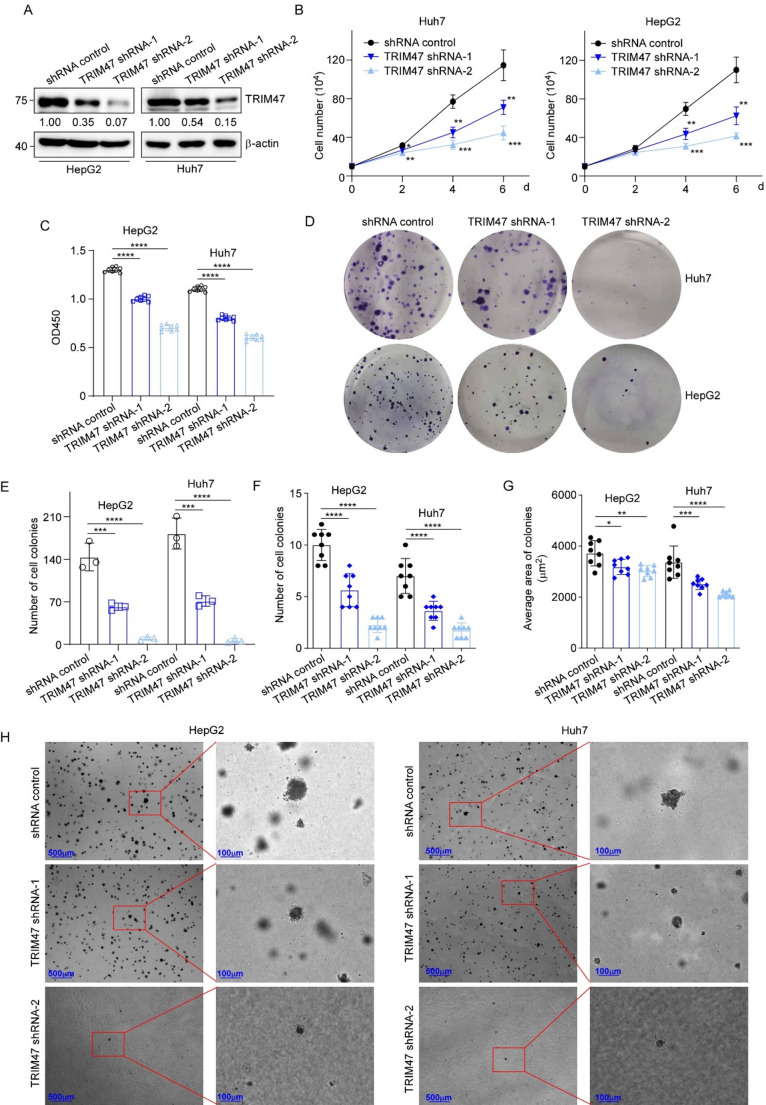
Knockdown of TRIM47 suppressed the proliferation of LC cells. **A.** Huh7 and HepG2 cells were infected with two types of lentivirus, TRIM47 shRNA (shRNA-1 and shRNA-2) or the control, to generate stable cell lines. Western blotting was used to detect the protein expression of TRIM47 in stable TRIM47-knockdown Huh7 and HepG2 cells and the corresponding control cells. **B.** The above cells were detected via a low-serum growth assay in 1% FBS medium, and cell counts were performed at 0, 2, 4, and 6 days. The data are presented as mean ± SD (*n* = 3). **C.** CCK-8 assay. The indicated cells were seeded into 96-well plate and then subjected to CCK-8 analysis. **D-E.** Colony formation assay. A total of 5 × 10^3^ cells were seeded in each well of a 6-well plate. Macroscopic clonal clusters appeared after 2–3 weeks and were fixed with 4% paraformaldehyde and stained with crystal violet. The number of clones was counted and analyzed statistically. **F-H.** Soft agar colony formation assay. The above stable LC cells were seeded in 6-well plates in full growth medium containing 0.35% agar (1 mL per well) on top of a layer of growth medium containing 0.6% agar (1 mL per well). After 20–25 days, the cells were imaged, and colonies larger than 50 μm were counted **F**. after which the area was calculated **G**. * *P* < 0.05, ** *P* < 0.01, *** *P* < 0.001 by one-way ANOVA followed by Tukey’s test

To investigate the oncogenic role of TRIM47 in LC, we examined its effects on proliferation and anchorage-independent growth in vitro. Using lentiviral-based transduction, we established two distinct TRIM47-knockdown cell lines (designated shRNA-1 and shRNA-2) in both Huh7 and HepG2 cells (Fig. 1A), along with stable TRIM47-overexpressed Hep3B cells for functional characterization (Figure S2A). The results of the cell counting and CCK-8 assays revealed that stable transfection of different shRNAs inhibited cell proliferation in both cell lines tested (Fig. 1B and C). As expected, downregulation of TRIM47 also inhibited colony formation in LC cells (Fig. 1D and E). In addition, we found that TRIM47 knockdown significantly reduced the anchorage-independent growth ability of LC cells via a soft agar assay (Fig. 1F-H).

Complementary gain-of-function experiments in Hep3B cells revealed that TRIM47 overexpression enhanced proliferation (Figure S2B and C) and promoted anchorage-independent growth in soft agar (Figure S2D and E). Taken together, these results indicate that TRIM47 exerts a pro-proliferative function in LC cells in vitro.

### TRIM47 promotes tumor growth in vivo

**Fig. 2:**
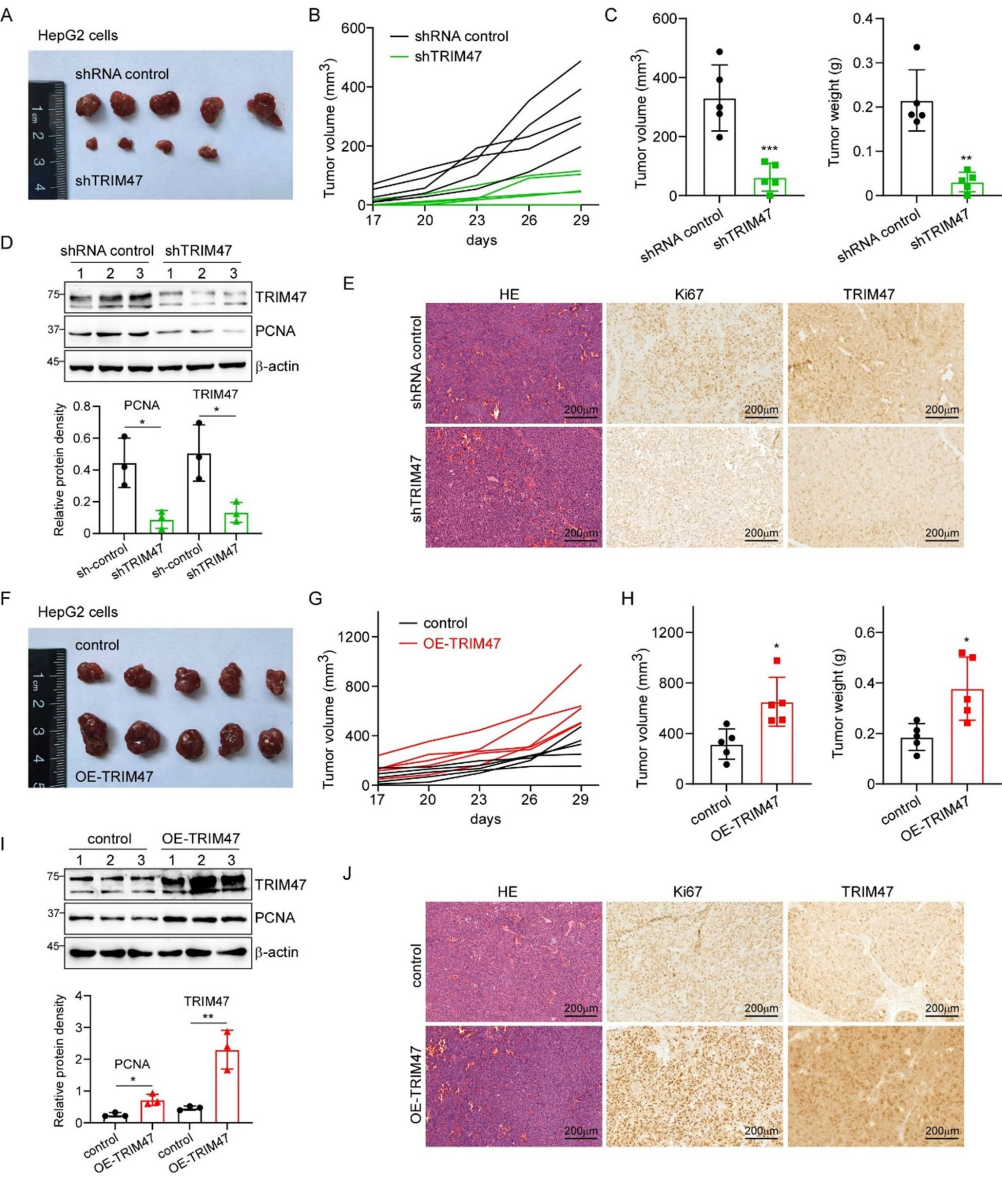
TRIM47 facilitated LC cell tumor growth in vivo. **A-C.** TRIM47 knockdown inhibited the formation of tumors with HepG2 cells in vivo. Five male nude mice received bilateral subcutaneous injections of 1 × 10^7^ TRIM47-knockdown stable cells or control cells in the dorsal cervical region. Seventeen days later, the tumor volume was measured every 3 days **B**, and the mice were sacrificed on day 29. Tumors were isolated, measured and weighed. Images of the tumors are shown in **A**, while data on the tumor volume and weight are listed in **C**. Data are presented as the means ± SD (*n* = 5). **D.** Downregulation of TRIM47 suppressed the protein expression of PCNA in vivo. Results are expressed as the mean ± SD (*n* = 3). **E.** Tumors were isolated, fixed, and subjected to HE and IHC assays. Silencing of TRIM47 inhibited the expression of the proliferation marker Ki67. **F-H.** TRIM47 overexpression promoted HepG2 cell growth in a xenograft model. Five male nude mice received bilateral subcutaneous injections of 1 × 10^7^ TRIM47-overexpressed stable cells or control cells in the dorsal cervical region. 17 days later, the tumor volume was measured every 3 days **G**, and the mice were sacrificed on day 29. Tumors were isolated, measured and weighed. The images of the tumors are shown in **F**, while the tumor volume and weight data are listed in **H**. The data are presented as the means ± SD (*n* = 5). **I.** Upregulation of TRIM47 increased the protein expression of PCNA in vivo. Results are expressed as the mean ± SD (*n* = 3). **J.** Tumors were isolated, fixed, and subjected to HE and IHC assays. TRIM47 promoted the expression of the proliferation marker Ki67. * *P* < 0.05, ** *P* < 0.01, *** *P* < 0.001 by Student’s t test

To investigate the oncogenic role of TRIM47 in LC tumorigenesis in vivo, we established xenograft models using stable TRIM47 knockdown/overexpressing HepG2 cells in male BALB/c-nu mice. Following validation of knockdown efficiency, TRIM47 shRNA-2 was selected for in vivo studies due to its superior interference efficacy compared to shRNA-1. Stable TRIM47-knockdown HepG2 cells (shRNA-2) and control cells were bilaterally implanted subcutaneously, with tumors harvested at the experimental endpoint (Fig. 2A). Tumor volumes were quantified every three days from day 17 post-implantation (Fig. 2B). Notably, TRIM47 depletion dramatically suppressed HepG2-derived tumor formation (Fig. 2C), demonstrating its critical role in LC growth in vivo. Results of immunoblotting and IHC assays revealed that knockdown of TRIM47 inhibited the expression of proliferation marker PCNA and Ki67 (Fig. 2D-E). Parallel experiments with TRIM47-overexpressed tumor cells revealed a significantly enhancement in tumor growth compared to the control group (Fig. 2F-H), with tumor volume and weight measurements showing consistent results between both cell models. Molecular analysis confirmed that TRIM47 upregulation elevated PCNA and Ki67 protein levels, as demonstrated by western blot (Fig. 2I) and immunohistochemistry (Fig. 2J). Furthermore, stable TRIM47 knocking-down Huh7 cells and overexpressing Hep3B cells (and related control cells) were used to generate subcutaneous xenograft modes to verify the effect of TRIM47 on tumor growth. Results of Figure S3 showed that knockdown of TRIM47 significantly suppressed the tumor formation, while upregulation of TRIM47 increased both the volumes and weighs of subcutaneous tumors. Taken together, these results demonstrate that TRIM47 plays a key role in promoting the growth of LC xenograft tumors.

### TRIM47 knockdown induces G2/M phase arrest and promotes apoptosis in LC cells

**Fig. 3:**
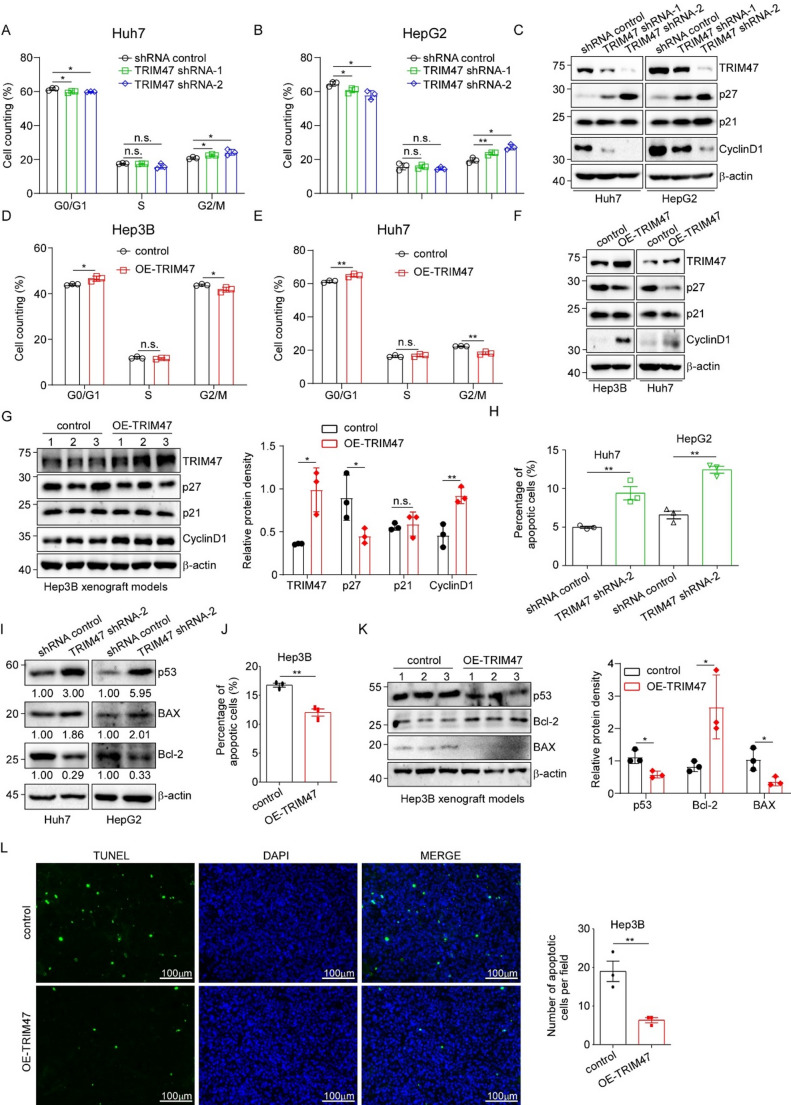
Effects of TRIM47 on cell cycle and apoptosis of LC cells. **A/B.** The cell cycle distribution of Huh7/HepG2 shRNA control and shTRIM47 (shRNA-1 and shRNA-2) stable cells was detected by flow cytometry, figures revealed the proportion of cells in each phase of the cell cycle (*n* = 3). **C.** Cell cycle-related proteins in the above two cell lines were detected via western blot. **D/E.** The cell cycle distribution of control and TRIM47-overexpressed Hep3B/Huh7 cells was detected by flow cytometry (*n* = 3). **F.** Cell cycle-related proteins in the above two cell lines (Hep3B and Huh7) were detected via western blot. **G.** Western blot was used to detect the expression of cycle-related proteins in Hep3B tumor tissues. Right panel, quantification of the western blot bands was carried out using ImageJ software. **H.** Huh7 and HepG2 shRNA control and shTRIM47 stable cells were used for cell apoptosis detection, the percentage of apoptotic cells was quantified, and the results were statistically analyzed. **I.** Western blot was used to detect the protein expression of apoptosis-related markers in the above cells (reference β-actin). **J.** The level of apoptosis in the Hep3B stable cell lines was detected via flow cytometry. **K.** Western blot was used to detect the expression of apoptosis-related proteins in Hep3B tumor tissues. Right panel, quantification of the western blot bands was carried out using ImageJ software. **L.** The levels of apoptosis in control and TRIM47-overexpressed Hep3B tumor tissues were determined via a TUNEL apoptosis kit, with blue fluorescence representing nuclei and green fluorescence representing apoptotic cells. The number of apoptotic cells per field was calculated and is shown in the right panel. All results are expressed as the mean ± SD. n.s. *P* > 0.05, * *P* < 0.05, ** *P* < 0.01

Given that dysregulated cell proliferation in malignancies frequently correlates with cell cycle aberrations and apoptotic resistance, and considering the established role of TRIM47 in modulating these processes across various tumor types [30], we sought to investigate its specific functions in LC. First, PI staining followed by flow cytometry analysis was used for the cell cycle experiments. Compared with the control group, TRIM47 knockdown (shRNA-1 and shRNA-2) resulted in cell cycle retardation at the G2/M phase in both the Huh7 and HepG2 cell lines (Fig. 3A and B). We then detected the expression of several critical cycle-related proteins, including p27, p21 and Cyclin D1. Western blot analysis revealed that TRIM47 knockdown significantly upregulated the cyclin-dependent kinase inhibitors p27, while downregulating Cyclin D1 expression (Fig. 3C). Consistent with the above results, TRIM47 overexpression in both Hep3B and Huh7 cells markedly increased the G0/G1 phase proportion while attenuating G2/M phase accumulation (Fig. 3D and E). Moreover, overexpression of TRIM47 significantly reduced the expression of cyclin-dependent kinase inhibitor p27 and increased the expression of Cyclin D1 (Fig. 3F). Similar results were obtained in Hep3B xenograft tumor tissues (Fig. 3G).

To investigate the effect of TRIM47 on LC apoptosis, we used Annexin V/PI apoptosis kits combined with flow cytometry. In both LC cell lines (Huh7 and HepG2) tested, TRIM47 knockdown significantly promoted cell apoptosis compared with the control cells (Fig. 3H). Then, western blot was used to detect apoptosis-related indicators in the above cell lines with TRIM47 knockdown and in the control group. TRIM47 depletion significantly upregulated pro-apoptotic proteins p53 and BAX while downregulating anti-apoptotic Bcl-2 expression compared to control cells (Fig. 3I). In the Hep3B cell line, TRIM47 overexpression reduced the percentage of apoptotic cells (Fig. 3J), as evidenced by decreased p53 and BAX expression alongside upregulated Bcl-2 levels in tumor tissues (Fig. 3K), which was consistent with the results of TRIM47 knockdown. Furthermore, the TUNEL staining results indicated that the number of apoptotic cells was reduced in tumor tissues overexpressing TRIM47 (Fig. 3L). These results together proved that TRIM47 plays a role in shortening the cell cycle and reducing apoptosis in LC cells.

### TRIM47 directly interacts with PLK1

**Fig. 4:**
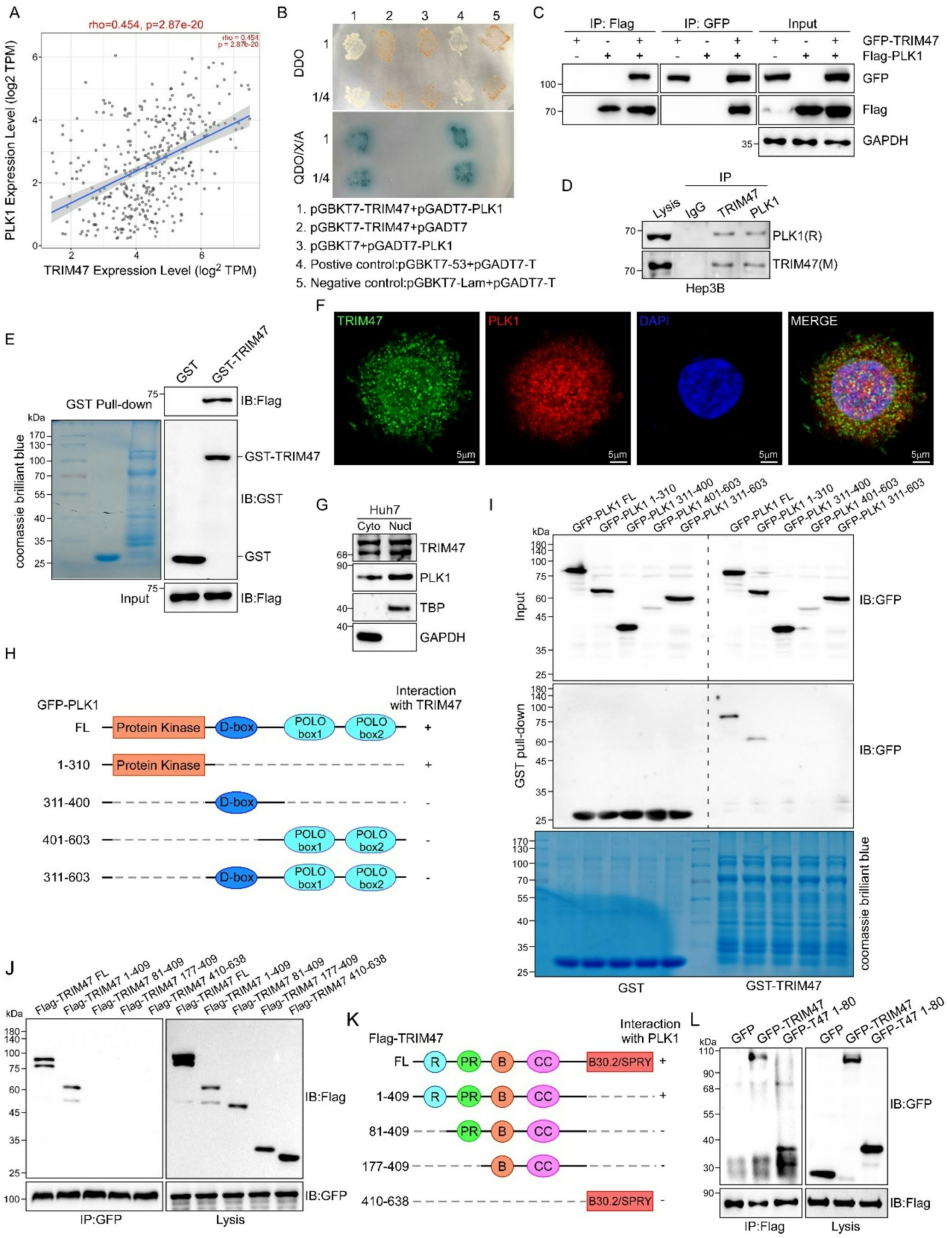
TRIM47 interacted with PLK1. **A.** Correlation between TRIM47 and PLK1 mRNA levels in clinical HCC samples was analyzed via the TIMER database. **B.** The interaction between TRIM47 and PLK1 was verified via a yeast two-hybrid system. **C.** Co-immunoprecipitation analysis of the interaction between GFP-TRIM47 and Flag-PLK1 in HEK293T cells. **D.** Co-immunoprecipitation analysis and protein blotting were conducted in primitive Hep3B cells. **E.** The purified GST or GST-TRIM47 protein was incubated with the lysates of Flag-PLK1-transfected HEK293T cells. After the GST pull-down experiment, the eluted protein and cell lysates were analyzed via western blotting. The expression of the GST and GST-TRIM47 proteins was confirmed by Coomassie brilliant blue staining and an anti-GST antibody. **F.** Co-localization of TRIM47 and PLK1 in Huh7 cells. The TRIM47 proteins were stained with a green fluorescent antibody (Alexa Fluor 488), with red fluorescence (Alexa Fluor 594) representing PLK1 and blue representing the nucleus. **G.** Representative images of the cytosolic and nuclear location of TRIM47 and PLK1 measured by western blot in cytosolic and nuclear fractions, using TBP (TATA-binding protein) as the nuclear control and GAPDH as the cytoplasmic control. **H.** Schematic diagram of PLK1 deletion mutants generated in the pEGFP-C vector. **I.** The interaction of TRIM47 with PLK1 and its deletion mutants was determined by GST pull-down using lysates of HEK293T cells transfected with PLK1 and its deletion mutants as indicated. **J.** The interaction of GFP-PLK1 with Flag-TRIM47 and its deletion mutants was determined by co-IP in HEK293T cells co-transfected with GFP-PLK1 and Flag-TRIM47 deletion mutants. **K.** Schematic diagram of TRIM47 deletion mutants generated in pcDNA3.1-Flag vector. **L.** Co-IP and immunoblotting were performed in HEK293T cells co-transfected with Flag-PLK1 and either GFP-empty vector (EV), full-length GFP-TRIM47 (WT), or the N-terminal GFP-TRIM47 truncation mutant (1–80 aa) to characterize their interaction

Having established TRIM47 as an oncogene in LC with dual effects on cell cycle and apoptosis, we sought to identify its downstream targets. Using a yeast two-hybrid system screen combined with bioinformatic analysis (Figure S4), we identified PLK1 as a candidate interacting protein. Notably, the expression of PLK1 was found to be significantly positively correlated with TRIM47 in HCC tissues (Fig. 4A), which has also been reported to be strongly correlated with the cell cycle and apoptosis of LC cells [31, 32]. As shown in Fig. 4B, the interaction between PLK1 and TRIM47 was first examined via a yeast hybridization assay, which verified the binding of PLK1 and TRIM47 in the yeast system, similar to positive controls (pGBKT7-53 + pGADT7-T). Next, the binding of TRIM47 and PLK1 was confirmed through co-immunoprecipitation assays in two experimental systems: (i) HEK293T cells co-transfected with GFP-TRIM47 and Flag-PLK1 expression vectors (Fig. 4C), and (ii) endogenous protein complexes in native Hep3B cells (Fig. 4D). Both systems consistently demonstrated a specific interaction between TRIM47 and PLK1. To further validate the direct interaction of TRIM47 with PLK1, GST and GST-TRIM47 were synthesized and purified from *E. coli* and incubated with cell lysates from PLK1 (Flag-PLK1)-transfected cells. As shown in Fig. 4E, PLK1 was pulled down by GST-TRIM47 but not by GST. To explore the subcellular localization relationship between TRIM47 and PLK1, immunofluorescence confocal assay showed that endogenous TRIM47 and PLK1 were co-localized in the nucleus and cytoplasm of Huh7 cells (Fig. 4F). In addition, the localization of TRIM47 and PLK1 was determined by western blot in the cytosolic and nuclear fractions, respectively. The results showed that these two proteins were expressed both in the cytoplasm and nucleus, although slightly more in the nucleus (Fig. 4G).

Based on the secondary structural characteristics of TRIM47 and PLK1, we constructed mutants containing different domains to identify the specific interaction sites between TRIM47 and PLK1. First, we transfected GFP-PLK1 or GFP-PLK1 mutants (protein kinase, D-box, POLO-box, D-box + POLO-box domains; Fig. 4H) into HEK-293T cells and collected cell lysates for GST pull-down assays. As shown in Fig. 4I, GST-TRIM47 pulled down full-length PLK1 and only the protein kinase fragments, suggesting that TRIM47 might interact with PLK1 fragments 1-310 aa. For TRIM47, we co-transfected GFP-PLK1 with Flag-TRIM47 or Flag-TRIM47 mutants (1-409 aa, 81–409 aa, 177–409 aa, and 410–638 aa) into HEK-293T cells to verify the binding domain of TRIM47. After 24 h, the cell lysates were collected, and Co-IP was performed with an anti-GFP antibody. In addition to full-length TRIM47, only the mutant containing the RING domain (1-409 aa) could be co-immunoprecipitated with PLK1, indicating that PLK1 binds to the N-terminus of TRIM47 (Fig. 4J and K). Since the 81-409aa domain of TRIM47 cannot interact with PLK1, we further explored whether the 1-80aa of TRIM47 alone can bind to PLK1. Results of Fig. 4L indicated that the 1–80 amino acid fragment of the RING domain alone was able to bind to PLK1.

### TRIM47 stabilizes PLK1 by mediating K63-linked ubiquitination

**Fig. 5:**
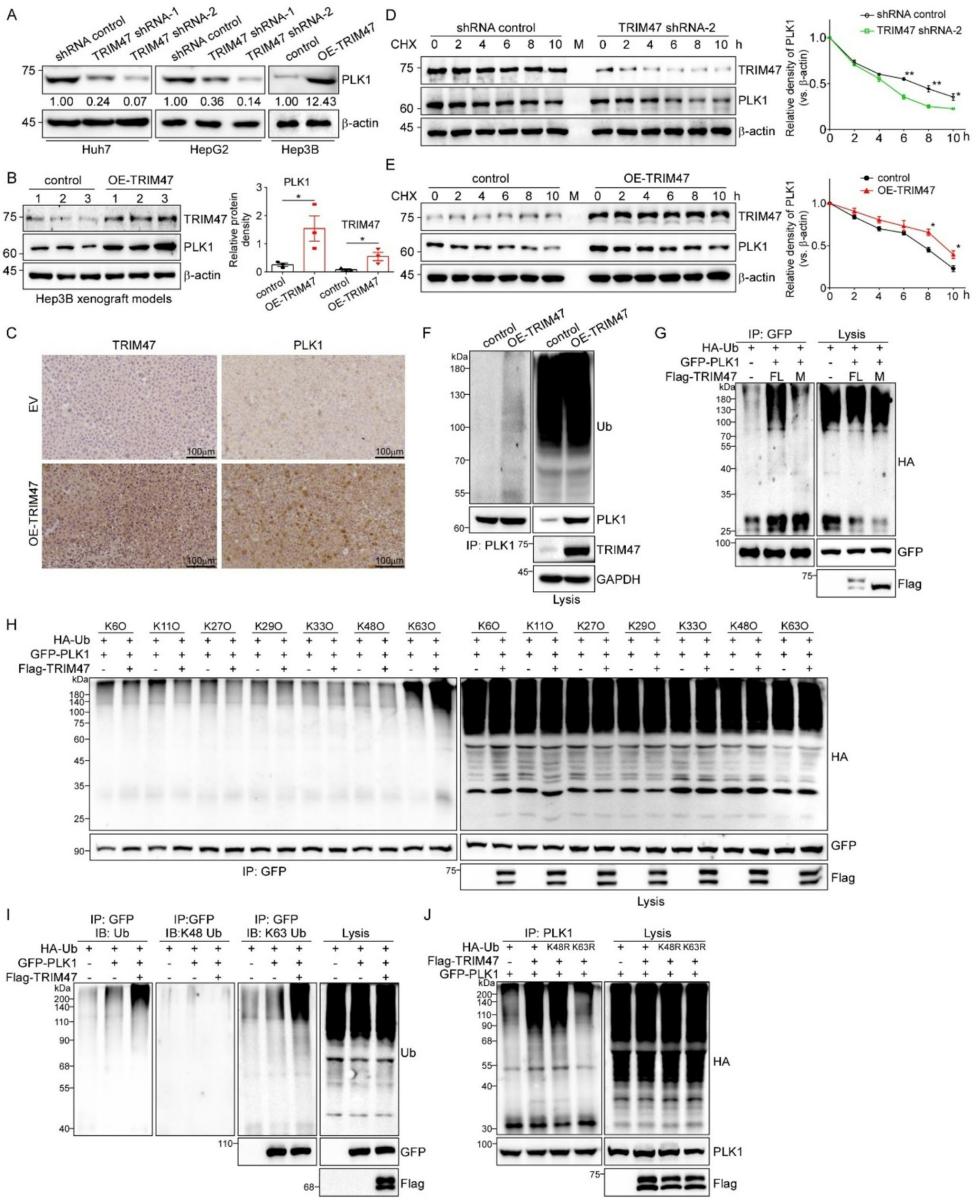
TRIM47 can stabilize PLK1 by mediating its K63-linked ubiquitination. **A.** Western blotting was used to detect the protein expression of PLK1 in stably transfected LC cells. **B.** The protein expression of TRIM47 and PLK1 in tumors from Hep3B xenografts was detected by western blot. **C.** Representative IHC staining of TRIM47 and PLK1 in Hep3B xenograft tumors. **D/E.** TRIM47-knockdown stable Huh7 cells/control cells **D.** or TRIM47-overexpressed stable Hep3B cells/control cells **E.** were treated with CHX (100 µg/mL) for 0, 2, 4, 6, 8, or 10 h. Western blotting was used to detect the protein expression of TRIM47 and PLK1 in the above cells, and ImageJ software was used for gray quantification analysis. * *P* < 0.05, ** *P* < 0.01. **F.** The ubiquitination level of the PLK1 protein was detected after immunoprecipitation with a PLK1 antibody in both TRIM47-overexpressed and control Huh7 cells. **G.** GFP-PLK1 was co-expressed with EV, Flag-TRIM47 (FL) or Flag-TRIM47 ΔRING (Mutant) in HEK293T cells, followed by anti-GFP IP and HA immunoblotting to examine PLK1 ubiquitination. **H.** HEK293T cells were co-transfected with GFP-PLK1 and seven different HA-ubiquitin (HA-Ub) mutants (K6O, indicating K6 only, which means that Ub ligation only occurs via the K6-linked pathway) with or without Flag-TRIM47 for 24 h. Then, the cell lysates were collected, immunoprecipitated with an anti-GFP antibody, and immunoblotted with an anti-HA antibody. **I.** HEK293T cells were co-transfected with GFP-PLK1 plus HA-Ub and/or Flag-TRIM47. After 24 h, the cell lysates were harvested, immunoprecipitated with anti-GFP antibody, and immunoblotted with the indicated antibodies. **J.** GFP-PLK1 and Flag-TRIM47 were co-transfected with HA-Ub, HA-Ub K48R or K63R into HEK293T cells. 24 h later, the cell lysates were immunoprecipitated with an anti-PLK1 antibody

Since TRIM47 has been reported and identified as an E3 ubiquitin ligase in previous studies [33], we aimed to investigate the effects of TRIM47 on PLK1 protein levels, ubiquitination status and molecular mechanism. First, we examined the protein expression of PLK1 in three stable LC cell lines and subcutaneous tumor tissues, and the results revealed a significant positive correlation between TRIM47 and PLK1 protein expression levels (Fig. 5A-C). Hence, TRIM47-knockdown Huh7 cells and TRIM47-overexpressing Hep3B cells with were treated with CHX, a protein synthesis inhibitor, for protein stability assays, respectively. As shown in Fig. 5D, compared with that in the control group (shRNA control), the degradation rate of the PLK1 protein in Huh7 cells was accelerated after TRIM47 knockdown, indicating decreased stability of the PLK1 protein. Treatment with the proteasomal inhibitor MG132 can markedly reverse the effect of TRIM47 knockdown on the protein stability of PLK1 (Figure S5). Consistently, overexpression of TRIM47 prolonged the half-life of the PLK1 protein to almost 10 h (Fig. 5E). These results indicated that TRIM47 could inhibit the proteasomal degradation of the PLK1 protein and stabilize it. Next, we investigated whether TRIM47 regulates the ubiquitination of PLK1 and how it works. First, we found that the ubiquitination of endogenous PLK1 was increased in TRIM47-overexpressed Huh7 stable cells (Fig. 5F) compared with that in control cells. Besides, overexpression of wildtype TRIM47 (full-length, FL) rather than TRIM47 mutant (RING finger domain deletion mutant, M) markedly increased the ubiquitination of PLK1, indicating that the RING finger domain of TRIM47 was indispensable for the PLK1 ubiquitination (Fig. 5G). Employing seven distinct HA-tagged ubiquitin mutants (K6O, K11O, K27O, K29O, K33O, K48O and K63O), we found that TRIM47 specifically mediates K63-linked ubiquitination of PLK1, rather than K48-linked or other ubiquitin chain types (Fig. 5H). Immunoblotting with linkage-specific ubiquitin antibodies (K48- and K63-specific) revealed that TRIM47 overexpression selectively enhanced K63-linked polyubiquitination of PLK1, while showing no effect on K48-linked ubiquitination (Fig. 5I). Moreover, the upregulation of TRIM47 was unable to regulate the ubiquitination of PLK1 when the HA-Ub K63R mutant existed, further confirming that TRIM47 can promote only the K63-linked ubiquitination of PLK1 (Fig. 5J).

### The NF-κB and MAPK pathways mediate TRIM47-induced proliferation of LC

**Fig. 6:**
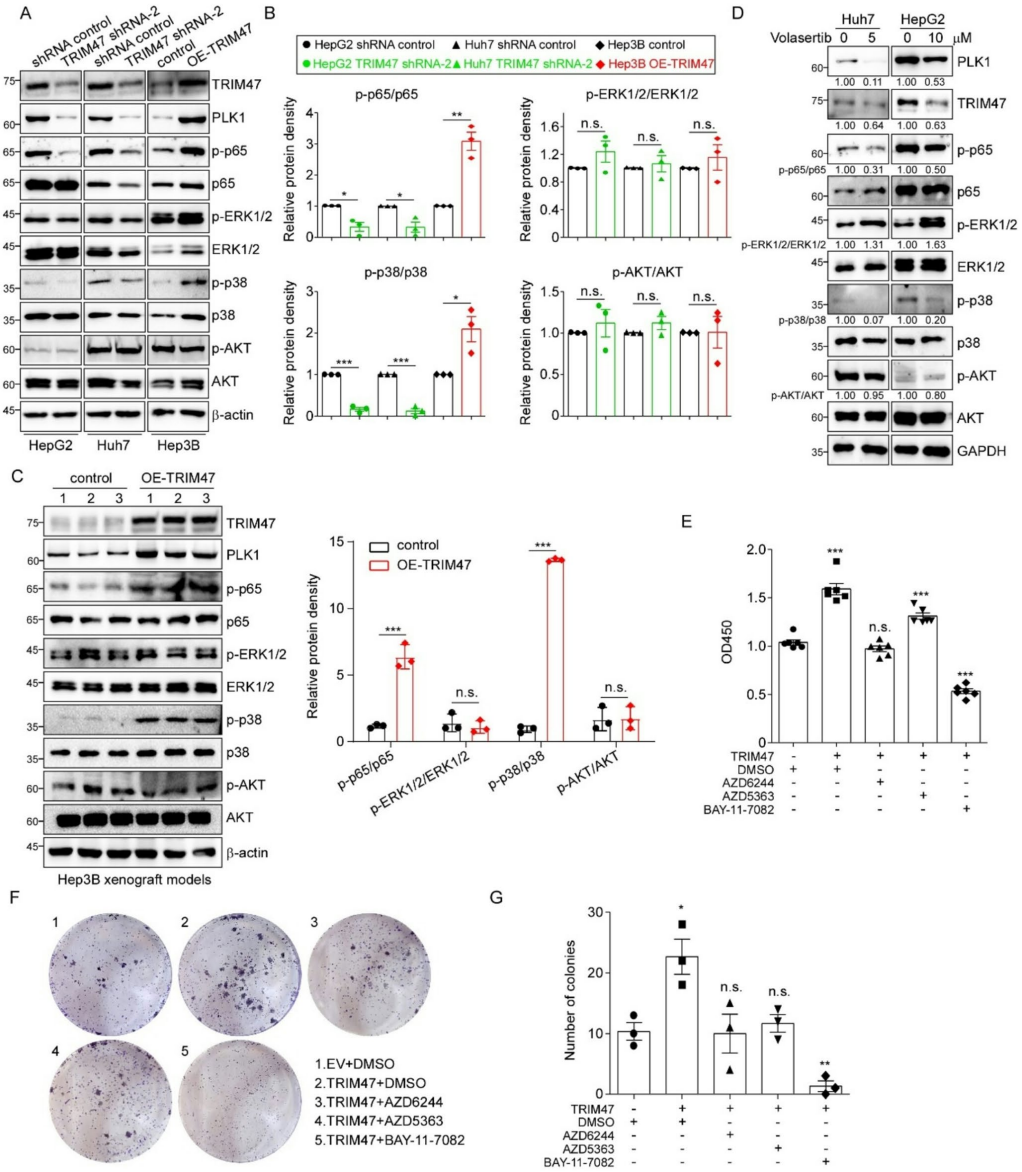
The NF-κB and MAPK pathways are involved in TRIM47-induced cell proliferation in LC. **A.** The total and phosphorylated levels of key proteins in the NF-κB, PI3K/AKT and MAPK pathways were detected in LC cells after TRIM47 silencing or overexpression. **B.** Protein quantitative analysis of A with 3 independent immunoblotting results. **C.** Western blotting was used to detect the activation of the above signaling pathways in Hep3B tumor tissues. The right panel shows the corresponding protein quantitative analysis. **D.** The total amount and phosphorylation levels of key proteins in the above signaling pathways were detected by western blotting after volasertib treatment. **E.** Control and TRIM47-overexpressed Huh7 stable cell lines were treated with AZD6244 (5 µM), AZD5363 (10 µM) or BAY-11-7082 (10 µM) for 48 h and then detected via a CCK-8, respectively. The OD450 value was detected and analyzed after treatment. **F.** Representative images of the colony formation assay in Huh7 cells after treatment with the above inhibitors. **G.** Statistical analysis of the number of cell clones with three replicates. The data are shown as the means ± SD. n.s. *P* > 0.05, * *P* < 0.05, ** *P* < 0.01, *** *P* < 0.001

The pathological process of malignant tumors is often related to abnormal activation of intracellular signaling pathways. Through an extensive literature review, we found that many classical signaling pathways are involved in the process by which PLK1 leads to tumor proliferation, among which the MAPK, PI3K-AKT and NF-κB signaling pathways are the most widely reported [34–37]. However, whether these pathways are activated in our system remains unknown. Therefore, we first evaluated the effects of TRIM47 on these three signaling pathways via western blot assay. The results in Fig. 6A and B indicate that the phosphorylation levels of p65 and p38, but not those of ERK1/2 or AKT, were positively correlated with the TRIM47 protein level in both the TRIM47-knockdown and TRIM47-overexpressed cell lines tested. The activation of related pathways was subsequently confirmed in TRIM47-overexpressed Hep3B tumor tissues. As shown in Fig. 6C, the phosphorylation levels of p65 and p38 were increased in TRIM47-upregulated tumors. In addition, we found that the phosphorylation levels of p65 and p38 were significantly reduced in LC cells after treatment with volasertib, a specific inhibitor of PLK1 (Fig. 6D). To further confirm the signaling pathways associated with TRIM47, we treated Huh7 cells with AZD6244, AZD5363 and BAY-11-7082, which are small molecule inhibitors of the MAPK, PI3K-AKT and NF-κB signaling pathways, respectively. Compared with control cells and DMSO treated stable TRIM47-overexpressed cells, TRIM47-overexpressed cells treated with AZD6244 or BAY-11-7082 could counteract the oncogenic effect of TRIM47 on cell proliferation and colony formation ability, with BAY-11-7082 showing the greatest effect (Fig. 6E-G). These findings suggest that the NF-κB signaling pathway plays a pivotal role in TRIM47-induced LC development while also highlighting the involvement of the MAPK pathway.

### TRIM47 promotes LC cell proliferation in a PLK1-dependent manner

**Fig. 7:**
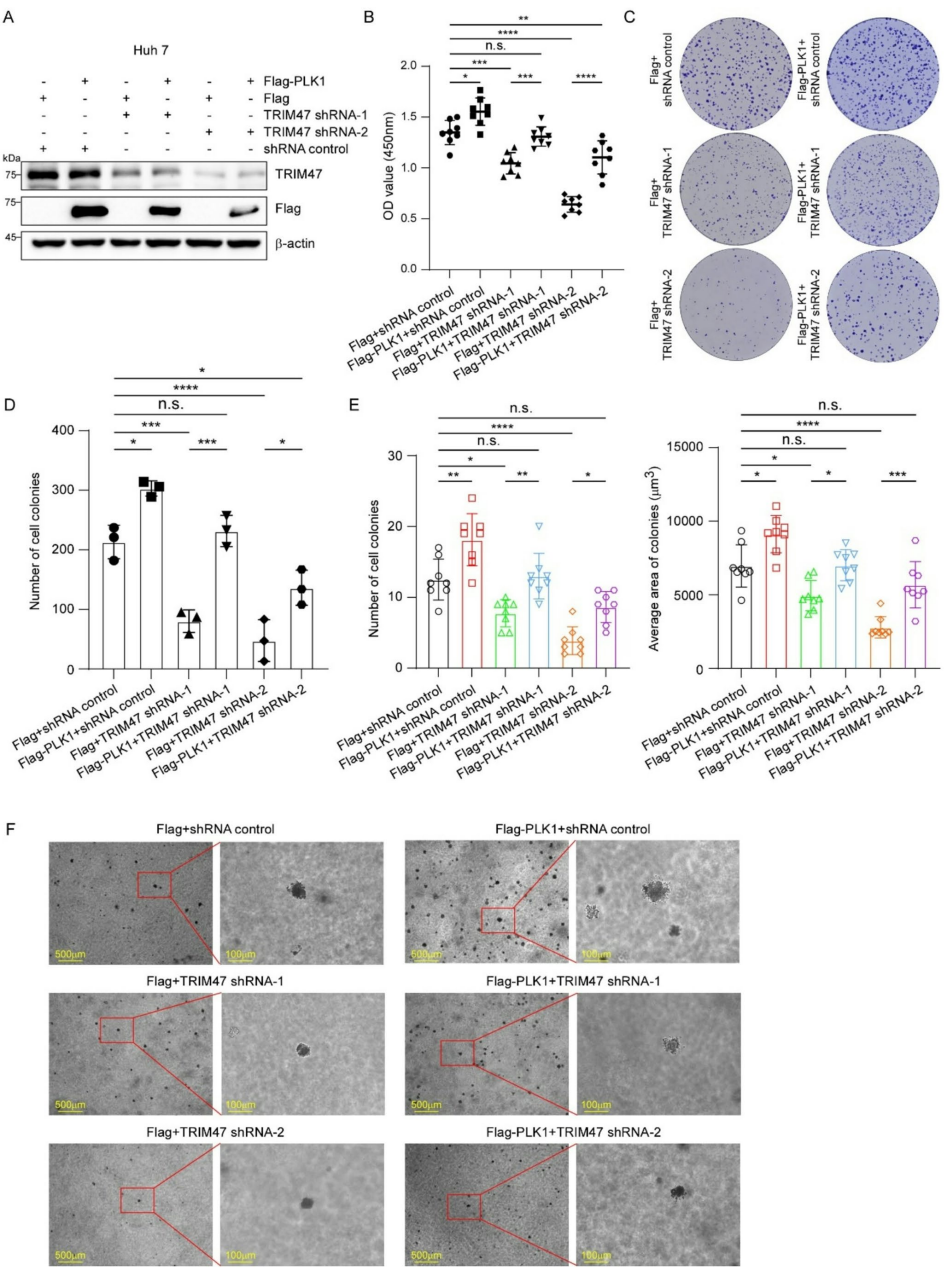
The effect of TRIM47 on LC cell proliferation is dependent on PLK1. **A.** Western blot analysis of 6 groups of co-transfected Huh7 cells (Flag (empty vector) + shRNA control, Flag-PLK1 + shRNA control, Flag + TRIM47 shRNA-1, Flag-PLK1 + TRIM47 shRNA-1, Flag + TRIM47 shRNA-2, Flag-PLK1 + TRIM47 shRNA-2). **B.** CCK-8 assay. The above cells were co-transfected with the indicated vectors. After 48 h, the cells were plated into 96-well plates and then subjected to CCK-8 analysis (*n* = 8). **C.** Colony formation assay. A total of 5 × 10^3^ cells from each group were plated in 6-well plates containing 5% FBS medium. After 10–14 days, the cells were fixed and stained with 2% crystal violet. **D.** The number of cell colonies was calculated and statistically analyzed (*n* = 3). **E-F.** Soft agar colony formation assay. 6 groups of Huh7 cells were seeded in 6-well plates in full growth medium (10% FBS) containing 0.35% agar (1 mL per well) on top of a layer of growth medium containing 0.6% agar (1 mL per well). After 20–25 days, the cells were imaged, and colonies larger than 50 μm were counted, after which the area was calculated (*n* = 8). n.s. *P* > 0.05, * *P* < 0.05, ** *P* < 0.01, *** *P* < 0.001, **** *P* < 0.0001 by one-way ANOVA followed by Tukey’s test

To determine whether TRIM47’s oncogenic effects require PLK1, we performed rescue experiments in Huh7 cells using six experimental groups (as schematized in Fig. 7A). CCK-8 assays showed that TRIM47 knockdown led to a significant decrease in cellular absorbance at 450 nm, an effect that was substantially rescued by PLK1 co-transfection in LC cells (Fig. 7B). Compared with the TRIM47-knockdown group, supplementation with the PLK1 protein also increased the colony-forming ability of LC cells, as expected (Fig. 7C-D). In addition, soft agar colony formation assays revealed that ectopic overexpression of PLK1 counteracted the inhibitory effect of TRIM47 knockdown on the anchorage-independent growth of Huh7 cells (Fig. 7E-F). The similar results were observed in HepG2 cells transfected with the above groups of plasmids as well (Figure S6). Together, these findings establish that TRIM47 promotes LC proliferation primarily through its regulation of PLK1.

### Inhibition of PLK1 activity counteracts TRIM47-driven LC proliferation in vivo

**Fig. 8:**
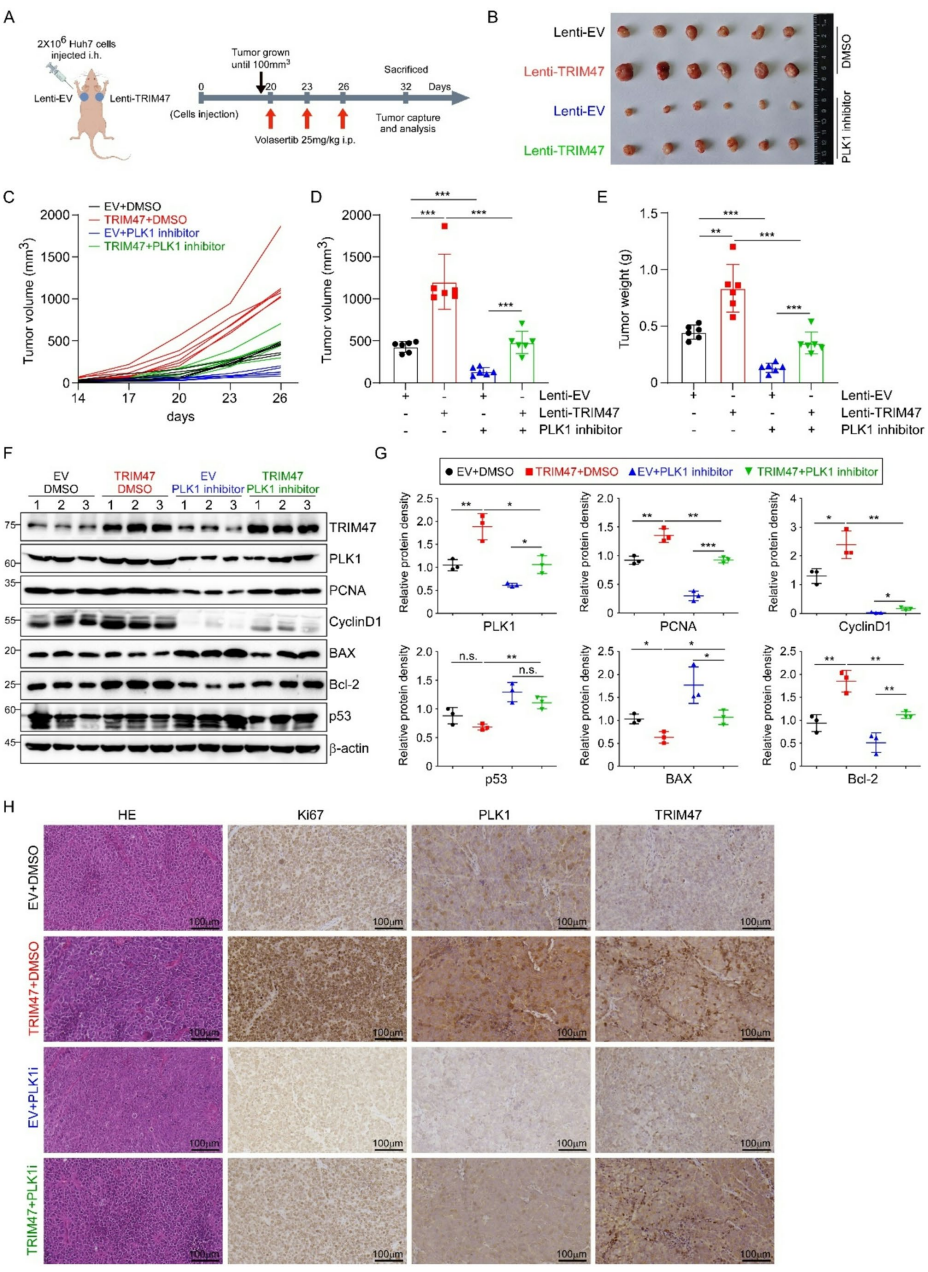
PLK1 inhibition attenuated the oncogenic effects of TRIM47 in vivo. **A.** Scheme of the xenograft model experiment. First, Huh7 cells stably expressing the empty vector (EV) or TRIM47 (2 × 10^6^) were subcutaneously injected into male BALB/c-nu mice. After the tumors reached 100 mm^3^, the mice were randomly divided into two groups (*n* = 6 for each group) and treated i.p. with control vehicle (DMSO) or the PLK1 inhibitor GSK461364 (25 mg/kg) at the same dose. Treatments were administered every 3 days for 3 doses. **B-E.** Overexpression of TRIM47 promoted the growth of Huh7 cells in a xenograft model, whereas a PLK1 inhibitor attenuated the oncogenic effect of TRIM47. 14 days after injection, the tumor volume **C**. was measured every 3 days, and the animals were sacrificed on the 26th day. Images of the tumors are shown in **B**, the tumor volume and weight data are listed in **D** and **E** (*n* = 6). **F.** The expression of proliferation-, apoptosis- and cyclin-related proteins in tumor tissues was detected via western blotting. **G.** Statistical analysis of the data in panel F (*n* = 6). **H.** Tumors were isolated and fixed, and relevant indicators were detected via HE and IHC. n.s. *P* > 0.05, * *P* < 0.05, ** *P* < 0.01, *** *P* < 0.001 by one-way ANOVA followed by Tukey’s test

**Fig. 9:**
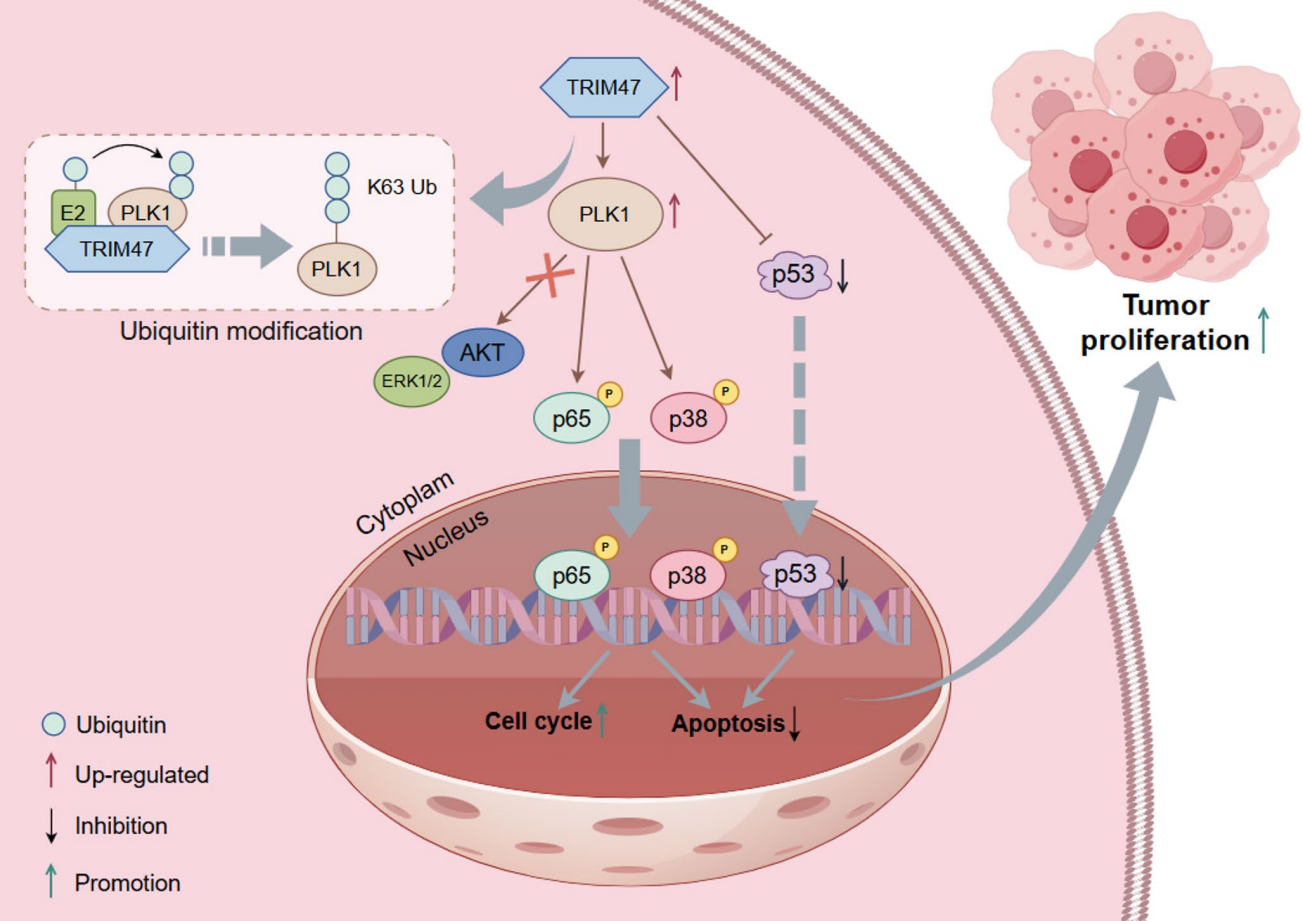
Working model. The expression of TRIM47 is up-regulated in LC cells, which increases the stability of PLK1 by ubiquitin modification and then phosphorylates p65 and p38 to promote their nuclear regulation, which leads to the reduction of cell apoptosis and shortening of cell cycle. Meanwhile, TRIM47 can promote the degradation of p53 and be involved in the regulation of cell apoptosis. Thus, the proliferation ability of LC cells was enhanced

Since we had demonstrated that TRIM47 promoted LC proliferation by stabilizing PLK1 through ubiquitination in vitro, we then intended to explore whether the regulatory mechanism was the same in the tumor xenograft model. Therefore, TRIM47-overexpressed Huh7 cells and the corresponding control cells were injected subcutaneously into both sides of male BALB/c-nu mice (Fig. 8A). The volume of the transplanted tumors was measured every 3 days starting on day 14, recorded and calculated as shown in Fig. 8C. When the average tumor volume reached 100 mm^3^, the mice were randomly divided into two groups, in which the experimental group was intraperitoneally injected with 25 mg/kg of the PLK1-specific inhibitor GSK461364 every 3 days, whereas the control group was injected with the same volume of solvent for a total of three times, and the tumor volume was continuously recorded every 3 days. At the end of observation, the tumors of each group are exhibited in Fig. 8B. The results revealed that TRIM47 upregulation significantly increased the mean tumor volume and tumor weight, whereas inhibition of PLK1 expression significantly alleviated the effect of TRIM47 (Fig. 8D and E). These results indicated that TRIM47 promoted tumor growth in vivo by targeting PLK1. As shown by the results of western blotting (Fig. 8F and G) and IHC (Fig. 8H), the upregulation of TRIM47 increased the protein levels of the proliferation markers PCNA and Ki67 and the cycle marker cyclin D1, increased the expression of the apoptosis marker Bcl-2 and decreased the expression of BAX and p53, while their expression returned to normal after PLK1 inhibition. These results collectively demonstrated that PLK1 is a downstream target protein of TRIM47, which plays a key role in the TRIM47-mediated growth of LC xenograft tumors. In summary, TRIM47 was validated to promote the proliferation of LC cells both in vitro and in vivo via PLK1 and related signaling pathways (Fig. 9).

## Discussion

LC is characterized by high malignancy, easy metastasis and poor prognosis and is estimated to cause at least 830,180 deaths per year [38]. Although many emerging systemic therapies, including new molecularly targeted monotherapies (e.g., ramucirumab) and new combination therapies (e.g., bevacizumab in combination with atezolizumab), have shown encouraging results in clinical trials [7, 39], only a small number of patients can achieve sustained clinical benefit, and the treatment of advanced LC is still a worldwide problem. In recent years, the role of TRIM47 in tumorigenesis has received increasing attention. As a member of the E3 ubiquitin ligase TRIM family, TRIM47 often plays a role in promoting different cancers as a proto-oncogene by modulating the ubiquitination of key proteins involved in tumor progression, such as promoting the development and drug resistance of triple-negative breast cancer by mediating BRCA1 protein degradation [40], facilitating aerobic glycolysis in pancreatic cancer cells by ubiquitinating and degrading FBP1 [41], promoting the malignant behavior of renal cell carcinoma by ubiquitinating and degrading p53 [42], etc. However, the oncogenic role of TRIM47 in LC has only been experimentally verified very recently [43], and the different regulatory mechanisms of TRIM47 in LC occurrence and development remain to be explored.

In this study, via bioinformatics database and tissue microarray analyses, we found that TRIM47 was upregulated in HCC tissues and was significantly correlated with increased stage and overall survival. Therefore, we conducted in vitro experiments and found that TRIM47 knockdown significantly inhibited the proliferation of LC cells, whereas TRIM47 overexpression had the opposite effects. By establishing a subcutaneous xenograft model in immunodeficient nude mice, we further demonstrated that TRIM47 knockdown in LC cells can inhibit tumor proliferation, lead to G2/M cell cycle retardation and induce cell apoptosis, and vice versa. Given that p53 is a key regulator in the apoptosis process, we further investigated whether TRIM47 affects the degradation of p53 in hepatic cancer cells. Results of Figure S7 showed that TRIM47 can modulate the degradation of p53 in a UPS dependent manner, indicating that TRIM47 may regulate tumor cell apoptosis via p53, the molecular mechanism needs further investigation. Moreover, we explored whether it has an effect on autophagy. Therefore, we examined p62 protein levels in LC stable cell lines, and western blot results showed that TRIM47 had no significant effect on p62 expression (Figure S8), suggesting that TRIM47 may not be involved in the regulation of autophagy in LC cells.

Mechanistically, we found that PLK1 is a downstream target of TRIM47 and verified their interaction through yeast two-hybrid experiments, and immunofluorescence analysis demonstrated their significant co-localization in LC cells. Further studies revealed that the overexpression of TRIM47 in LC cells promoted the K63-linked ubiquitination and stability of PLK1, thus regulating the activation of the NF-κB and MAPK signaling pathways and thereby shortening the cell cycle and accelerating proliferation. Therefore, our results suggest that TRIM47 has an oncogenic effect on LC and may be a potential target for its treatment.

Ubiquitination is one of the most important post-translational modifications and plays a vital role in the pathogenesis and development of LC [44, 45]. There is increasing evidence that targeting the ubiquitination system is a promising strategy for the treatment of malignant tumors, including LC. Previous studies have shown that TRIM47 has a variety of complex functions in cancer, including as an E3 ubiquitin ligase [46]. In this study, we comprehensively elucidated the regulatory effect of TRIM47 on PLK1 in LC for the first time. As an E3 ligase, TRIM47 targets and stabilizes PLK1 through ubiquitin modification. Notably, screening of seven ubiquitination sites revealed that TRIM47 specifically enhances the K63-linked polyubiquitination of PLK1, thereby exacerbating LC lesions. Previous studies have demonstrated that K48-linked polyubiquitin chains are commonly associated with the proteasomal degradation of certain substrates via the ubiquitin-proteasome system. Conversely, K63-linked ubiquitylation can modulate various protein characteristics, including protein‒protein interactions, translocation, and activation, among different biological processes [47, 48]. In some tumor cells, K63 ubiquitination can promote tumor progression by stabilizing certain proteins. For example, FBXL6 promotes K63-dependent ubiquitination of HSP90AA1 and stabilizes it in HCC [49], which is consistent with the role of K63-linked ubiquitination observed in our study. This is a new discovery concerning the pathogenesis of LC and the downstream signaling pathways involved.

PLK1, a member of the Polo-like kinase family, is widely present in eukaryotic cells and participates in the initiation, maintenance, and termination of mitosis as a serine/threonine protein kinase [50, 51]. Recent studies have shown that PLK1 is also involved in the development of malignant tumors, including LC [52, 53]. However, the relationships among TRIM47, PLK1 and downstream signaling pathways remain to be elucidated. In our study, we found that PLK1 can bind to TRIM47. The RING domain (1–80 aa) of TRIM47 and the kinase domain of PLK1 (1-310 aa) contribute to this interaction. Therefore, we studied the functional significance of TRIM47 as an E3 ubiquitin ligase that interacts with PLK1 and reported that TRIM47 is an upstream regulator of PLK1 and promotes its ubiquitination. Although PLK1 is known to be regulated by key oncogenic pathways, such as AURKA (which phosphorylates Thr137 /210 to activate PLK1 during G2/M phase) [54], FOXM1 (which binds the PLK1 promoter to enhance transcription) [55], and E2F1 (which activates PLK1 expression in prostate cancer by promoting the transcription of ORC6) [56], Our findings uncover a previously unrecognized TRIM47-PLK1 regulatory axis, in which TRIM47 modulates PLK1 primarily through ubiquitination-related mechanisms.

We subsequently analyzed the existing research literature and found that the MAPK, PI3K/AKT and NF-κB pathways may be the key pathways involved in LC progression related to PLK1. Therefore, we examined the effects of TRIM47 on the activation of these signaling pathways in liver cancer cells. TRIM47 was verified to regulate the levels of p-p65 and p-p38 in LC cells, corresponding to the activation of the NF-κB and P38/MAPK pathways, respectively. Upon the inhibition of PLK1 by volasertib, the activation of the NF-κB and P38/MAPK pathways was obviously suppressed. After treatment with BAY-11-7082, a specific inhibitor of the NF-κB pathway, the tumor-promoting effect of TRIM47 was significantly inhibited in Huh7 cells. In addition, the MAPK pathway specific inhibitor AZD6244 can also partially counteract the oncogenic function of TRIM47. Therefore, we found that the activation of the NF-κB and MAPK pathways, especially the NF-κB pathway, is very important for the TRIM47-mediated induction of LC cell proliferation. Notably, data from the THPA database revealed a significant sex-specific difference in the impact of TRIM47 on HCC survival. The opposite trend in females underscores the complexity behind this association, warranting further investigation into the underlying mechanisms.

Since the regulatory interaction between PLK1 and TRIM47 was a major focus of this study, the key role of PLK1 in this axis was further confirmed via rescue experiments. On the basis of the proven tumor-promoting effect of TRIM47, a PLK1 plasmid was transfected into TRIM47-knockdown LC cell lines to counteract the retardation of proliferation caused by TRIM47 interference. Similarly, inhibition of PLK1 in vivo effectively attenuated TRIM47-mediated tumor promotion in xenograft models. All these results effectively revealed that TRIM47 promotes LC progression in a PLK1-dependent manner. Owing to the dose-limiting toxicity and poor efficacy of PLK1 inhibitors as a single therapy, their clinical application has not yet been achieved [57]. However, the combination of PLK1 inhibitors is a promising method for the treatment of tumors. Elodie Montaudon et al. reported that the combination of mTORC1 and PLK1 inhibitors has high synergistic antitumor activity in adenocarcinoma NSCLC [58]. Therefore, the combination of a PLK1 inhibitor and TRIM47 inhibition may serve as a new option for the clinical treatment of LC.

In conclusion, we demonstrated that TRIM47 is an important tumor-promoting factor in LC. Mechanistically, TRIM47, an E3 ubiquitin ligase, promotes the K63-linked polyubiquitination and stability of PLK1, thereby promoting the activation of the NF-κB cascade during LC progression. The TRIM47/PLK1/NF-κB signaling cascade is a potential preventive and therapeutic target for LC. In addition, TRIM47-mediated modulation of PLK1 ubiquitination and stabilization affected the p38/MAPK pathway. More interestingly, pharmacological inhibition of PLK1 in LC cells led to concomitant reduction in TRIM47 expression at both translational and transcriptional levels (Figure S9), indicating that TRIM47 may be a downstream gene of PLK1. The expression of TRIM47 and PLK1 mutually promotes each other, forming a positive feedback loop to further enhance the malignant biological behavior of LC, which needs further elucidation.

## Materials and methods

### Cell culture

Human liver cancer cell lines (Huh7, HepG2, Hep3B) were purchased from Wuhan Procell Life Science and Technology Ltd., China, between 2019 and 2023. HEK293T (HEK-293T human embryonic kidney cells) were kindly provided by Dr. Chao Shen and Dr. Congyi Zheng (College of Life Sciences, Wuhan University) as gifts in March 2019. In December 2019, Huh7, HepG2, Hep3B and HEK293T cells were identified by short tandem repeat (STR) analysis, and PCR analysis was recently negative for mycoplasma contamination. The cells were routinely cultured in MEM and DMEM (Servicebio, Wuhan, China) supplemented with 10% FBS (ExCell Bio, Taicang, China) and 1% penicillin and streptomycin at 37 °C and 5% CO_2_.

### Reagents

Antibodies and reagents used in the study are described below: TRIM47 (26885-1-AP), β-actin (66009-1-Ig), GAPDH (60004-1-Ig), Flag tag (20543-1-AP/66008-4-Ig), GFP tag (50430-2-AP/66002-1-Ig), BAX (50599-2-Ig), Bcl-2 (12789-1-AP), cyclin D1 (60186-1-Ig), p53 (10422-1-AP), Ki67 (27309-1-AP), PCNA (10205-2-AP), and HA tag (66006-2-Ig) antibodies were purchased from Proteintech (Wuhan, China). PLK1 (A2548) antibodies were purchased from ABclonal (Wuhan, China). p65 (8242S), p-p65 (3033S), Erk1/2 (4695S), p-Erk1/2 (4370S), p21 (2947S), p27 (3686S), p38 (9212S), p-p38 (4511S), Akt (9272S), p-Akt (4060S), K63-linked specific Ub (5621S), K48-linked specific Ub (8081S), and Ub (3936S) were purchased from Cell Signaling Technology (Danvers, MA, USA). TRIM47 (H00091107-M02) was purchased from Abnova (Taibei, China). The lentiviruses pLV-Scramble [shRNA-NC], pLV-shTRIM47 [shRNA-1] (sequence: 5’-TGAAGCTCCCAGGGACTATTT-3’) and pLV-shTRIM47 [shRNA-2] (sequence: 5’-TACTGGGAGGTGGAGATTATC-3’) were purchased from Cyagen Biosciences (Suzhou, China). Inhibitors Volasertib, BAY11-7082, Capivasertib (AZD5363), and Selumetinib (AZD6244) were purchased from Topscience (Shanghai, China).

### Cell proliferation assay

For the low-serum growth experiment, the cells were seeded in 12-well plates (10^5^ cells/well), and three replicates were made for each cell sample. After being attached to the wall, the cells were grown in medium containing 1% serum, and the cells on day 0 were counted at the same time. The medium was changed every 2 days, and the cells on days 2, 4, and 6 were counted at regular intervals. For the CCK-8 cell activity assay, cells were inoculated into 96-well plates at a density of 5000 cells per well. After 12–24 h, CCK-8 reagent (Yeasen Scientific, Shanghai, China) with a 1/10 volume of medium was added, and the cells were cultured in an incubator at 37 °C for 0.5-4 h. The absorbance at 450 nm was detected with a microplate reader.

### Colony formation and soft agar experiments

For the colony formation assay, cells were seeded into 6-well plates at a gradient density of 5 × 10^2^ or 1 × 10^3^ cells per well and cultured for approximately 2 weeks. When visible clones were found in the culture dish, the cells were fixed at 4 °C overnight, dyed with 2% crystal violet solution for 1–2 h, washed slowly with running water and dried at room temperature. The number of clones containing more than 50 cells was counted under a microscope and photographed.

For the soft agar assay, preheated 2 × 1640 medium was mixed with an equal volume of 1.2% agarose solution, added to the lower layer of a 6-well plate (1 mL per well) and cooled to solidification at 4°C. The preheated 2 × 1640 medium was subsequently mixed with an equal volume of 0.7% agarose solution and a suspension containing 1 × 10^4^ cells and then added to the upper layer of the 6-well plate at a volume of 1 mL/well. After cooling to coagulation, the cells were cultured at 37 °C and 5% CO_2_ for 14–21 days. The colonies with a diameter of ≥ 50 μm were imaged and counted, and the area of each colony was calculated.

### Western blot and co-immunoprecipitation (Co-IP)

The cells were harvested or lysed with 2× loading buffer or lysis buffer, and after BCA quantitation (Glpbio, Shanghai, China), the whole cell lysates were analyzed via western blotting as described previously [59]. Subcellular fractionation was performed to isolate nuclear and cytoplasmic proteins using a commercial extraction kit (Abbkine Scientific, Wuhan, China) according to the manufacturer’s protocol. Subsequently, 50 µg aliquots of each protein sample were electrophoresed on SDS-polyacrylamide gels. The protein density was analyzed via ImageJ software and normalized to that of the endogenous housekeeping protein (GAPDH or β-actin). For the co-IP experiments, the transfected HEK293T cells or primitive Hep3B cells were lysed with 0.5% Lubrol-Px lysis buffer (50 mM KCl, 2 mM CaCl_2_, 20% glycerol and 50 mM Tris-HCl, pH 7.4). The supernatant was mixed with 0.5-2 µL of antibody and incubated at 4 °C for 2–4 h. After that, 120 µL of protein G beads (Beyotime Biotechnology, Shanghai, China) were added to each tube and incubated overnight at 4 °C with gentle rotation (10 r/min). On the second day, 500 g centrifugation was used to discard the supernatant, and after washing three times, the binding protein was analyzed by immunoblotting according to the above method.

### Quantitative real-time PCR (RT-qPCR)

Total RNA was reverse transcribed into the first strand of complementary DNA (cDNA) via a reverse transcription kit (Yeasen Scientific, Shanghai, China). RT-PCR was performed on a ViiA-7 real-time PCR system instrument (ABI, Waltham, MA, USA) with a SYBR-Green PCR Master Mix Kit (Yeasen Scientific, Shanghai, China). We selected β-actin as an endogenous control and calculated the relative level of the indicator gene via the 2^−ΔΔCt^ method. The sequences of primers used are listed in Table S1.

### Cell cycle experiments

The cells were digested with trypsin, harvested, and then centrifuged at 200 × g for 6 min at room temperature. The supernatant was removed, resuspended in medium containing serum (to inactivate trypsin) and centrifuged again to remove the supernatant. Cell counts were performed, and 1 × 10^6^ to 1 × 10^7^ cells were thoroughly resuspended in 0.5 mL of PBS. To prepare for fixation, 4.5 mL of 70% ethanol fixative was added to 15 mL centrifuge tubes, and the tubes were placed on ice. A total of 0.5 mL of the above cell suspension was transferred to a tube and fixed on ice for more than 2 h. Ethanol was removed thoroughly by centrifugation. The cell precipitate was suspended in 5 mL of PBS, and the supernatant was subsequently discarded by centrifugation again. The cells were resuspended in 1 mL of propidium iodide (PI) staining solution and then incubated at 37 °C for 15 min or at room temperature for 30 min. Detection was performed via a PE flow cytometer (CytoFLEX, Beckman Coulter). PI staining solution: 20 mg of RNase A and 2 mg of PI were added to 100 mL of PBS containing 0.1% (v/v) Triton X-100, pH 7.4.

### Cell apoptosis assay

After digestion with trypsin without EDTA, the cells were collected via centrifugation at 300 × g for 5 min at 4 °C. The cells were washed twice with precooled PBS and centrifuged at 300 × g for 5 min at 4 °C each time. The PBS was aspirated and discarded, and the cells were resuspended by adding 100 µL 1×Binding Buffer. 5µL Annexin V-Alexa Fluor 647 and 10 µL of PI (Yeasen Scientific, Shanghai, China) were added, and the mixture was gently mixed. The staining reaction was protected from light and incubated at room temperature for 15 min. Then, 400 µL of 1× binding buffer was added, mixed and placed on ice, and the samples were examined by flow cytometry within 1 h.

### TUNEL staining assay

Paraffin-embedded tissue sections were deparaffinized and dehydrated at room temperature. The sections were gently moistened and washed with PBS, and excess liquid was carefully blotted dry with filter paper. TUNEL staining of the tumors was performed via a TUNEL Apoptosis Detection Kit (Yeasen Scientific, Shanghai, China) according to the manufacturer’s instructions. 100 µL Proteinase K working solution was added to each sample and incubated for 20 min at room temperature. Then, 100 µL of 1× equilibration buffer was added to completely cover the sample area to be tested, and the mixture was incubated for 10–30 min at room temperature. After washing with absorbent paper around the equilibrated area, 50 µL of TdT incubation buffer was added to cells with an area of 5 cm^2^. The cells were incubated at 37 °C in a wet box in the dark for 60 min. The samples were stained in a staining cylinder after slow washing with PBS. After washing, the samples were immediately analyzed under a fluorescence microscope with a standard fluorescence filter setup for green fluorescence at 520 ± 20 nm and blue DAPI at 460 nm.

### Xenograft tumor model

For tumor models with LC cells, 4–6-week-old male BALB/c nude mice purchased from SPF Biotechnology (Beijing, China) were randomly assigned to each experimental group. A total of 1 × 10^7^ HepG2 cells or 5 × 10^6^ hepatoma cells (Huh7 and Hep3B) in PBS were resuspended in Matrigel (Corning, NY, USA) at a ratio of 1:1 and injected subcutaneously into the posterior neck of BALB/c nude mice, with 5–6 mice in each group. Approximately 2 weeks after injection, the tumor volume was measured with calipers every 2 to 3 days.

For the rescue experiment with the PLK1 inhibitor, Huh7 cells (2 × 10^6^) were inoculated into the posterior neck subcutaneous of male BALB/c-nu mice (4 weeks) (Beijing, China). The mice were randomly divided into two groups until the average size of the tumors reached 100 mm^3^ and received intraperitoneal (i.p.) injections of GSK461364 (25 mg/kg) or control vehicles respectively every 3 days for 3 doses in total.

Tumors were measured with a Vernier caliper, and tumor volume was calculated as follows: volume = length × width × width/2. All the mice were sacrificed at the end of observation, and the transplanted tumors were isolated for hematoxylin‒eosin (HE) staining, western blotting and IHC analysis. All studies were reviewed and approved by the Institutional Animal Care and Use Committee (IACUC) at Nanchang University (NCU). In no case was the maximum IACUC permissible tumor burden exceeded.

### Yeast two-hybrid

The full-length cDNA of the human *TRIM47* gene was subsequently subcloned and inserted into the pGBKT7 vector as bait, while the human *PLK1* gene was subsequently inserted into the pGADT7 vector as prey. The indicated combinations of vectors were transformed into Y2HGold yeast strain including negative controls (pGBKT7-Lam + pGADT7-T) and positive controls (pGBKT7-53 + pGADT7-T), and plated onto DDO (SD/-Leu/-Trp) and QDO/X/A (SD/-Ade/-His/-Leu/-Trp/X-α-Gal) plates. The blue colonies on higher stringency agar plates QDO/X/A were considered to have interactions.

### GST pull-down

The GST/GST-TRIM47 plasmid was transformed into BL21 *E. coli*, and its protein expression was induced by IPTG (0.5 mM) for 12 h. The GST/GST-TRIM47 protein was subsequently purified. HEK293T cell lysates transfected with Flag-PLK1 were incubated with GST-TRIM47 or GST (negative control) overnight at 4 °C. Protein complexes were precipitated with Pierce™ Glutathione Agarose (Thermo Scientific, Waltham, MA, USA), followed by SDS-PAGE (coomassie brilliant blue staining) and immunoblotting.

### Immunofluorescence (IF)

To detect the co-localization of TRIM47 and PLK1, Huh7 cells were pre-inoculated on glass slides one day before IF detection. When the cell density reached a range of 20% to 30%, the cells were fixed in 4% cold PFA overnight and incubated with 0.5% Triton X-100 for 10 min to make the cells transparent. After being blocked with 5% bovine serum albumin, the cells were incubated with TRIM47 primary antibody (1:500, Abnova) and PLK1 primary antibody (1:500, ABclonal) for 12 h at 4 °C. The next day, Alexa Flour 594/488 secondary antibodies were used to bind to TRIM47/PLK1 for 2 h at room temperature. The nuclei were then stained with DAPI (Servicebio, Wuhan, China), and photos of the slides were taken via a Zeiss LSM800 confocal microscope.

### Immunohistochemistry (IHC)

HE and IHC staining of tumors from the in vivo xenograft assay were performed as previously described [60]. Paraffin sections of tumor tissue were dewaxed with water, and antigen retrieval was performed. Endogenous peroxidase was blocked in 3% hydrogen peroxide solution. Then, 5% BSA was used for blocking. Ki67, TRIM47 and PLK1 primary antibodies and secondary antibodies were added. Finally, DAB staining solution was used for color development, and the nuclei were counterstained with hematoxylin. Images were captured with an FSX100 microscope equipped with a digital camera system (Olympus).

### Statistical analysis

The data obtained in this study were statistically analyzed via the GraphPad Prism 9 statistical software package, and the measurement data are expressed as the means ± standard deviations (means ± SD). Statistical comparisons between the two groups were performed using an unpaired Student’s t-test. For comparisons involving multiple groups with different variables, a one-way or two-way analysis of variance (ANOVA) was applied, followed by Tukey’s post hoc test for pairwise comparisons. *, *P* < 0.05; **, *P* < 0.01; ***, *P* < 0.001; ****, *P* < 0.0001.

## Supplementary Information

Below is the link to the electronic supplementary material.

Supplementary Material 1

## Data Availability

All datasets generated for this study are included in the article/Supplementary Material.
