# Supplementary material for: TRIM47-facilitated PLK1 stabilization promotes the proliferation of liver cancer cells
Source: Cell Oncol (Dordr). 2025 Dec 29;49(1):7. doi: 10.1007/s13402-025-01130-0 (PMC12748135; doi:10.1007/s13402-025-01130-0)

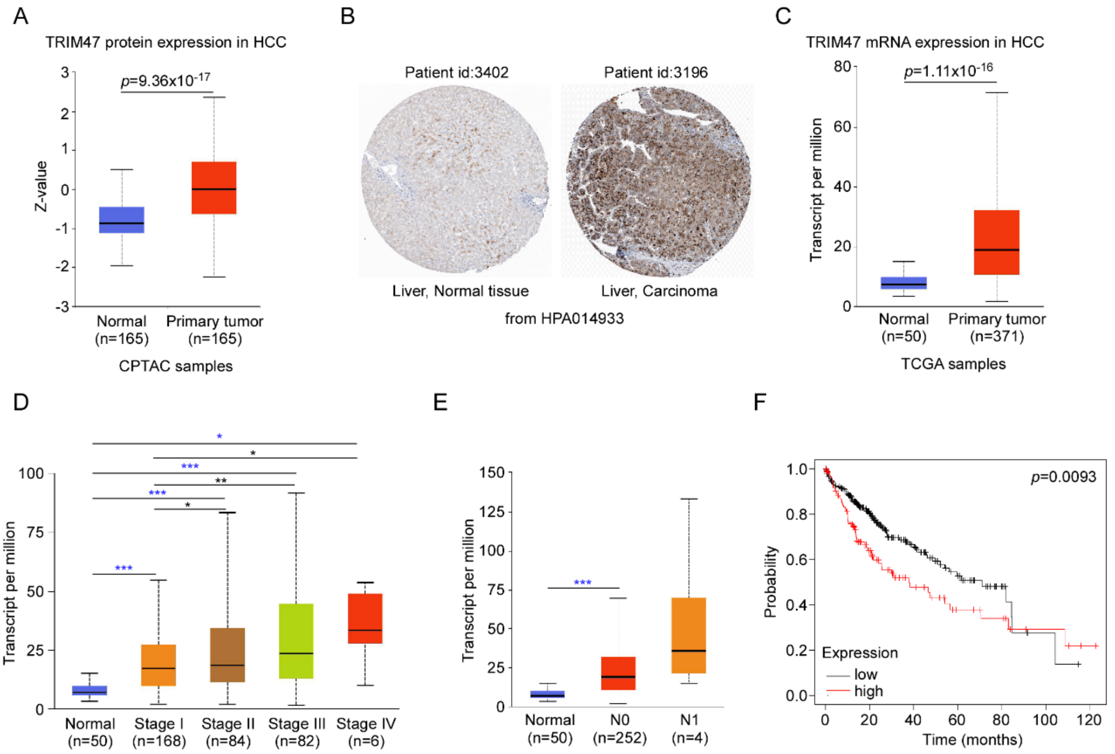

Figure S1: TRIM47 is highly expressed in human hepatic cell carcinoma tissues.
A. The expression level of TRIM47 protein in hepatocellular carcinoma tissues and normal liver tissues in UALCAN database. **B.** Representative IHC staining images of TRIM47 in clinical liver cancer tissues and normal liver tissue samples from HPA database. **C.** The mRNA levels of TRIM47 in hepatocellular carcinoma tissues and normal liver tissues in TCGA database. **D.** TRIM47 mRNA levels in normal livers and HCC tumors with different stages. **E.** UALCAN database was used to analyze the correlation between TRIM47 expression level and lymph node metastasis in patients with liver cancer. **F.** The prognostic value of TRIM47 for overall survival was determined by Kaplan-Meier analysis.

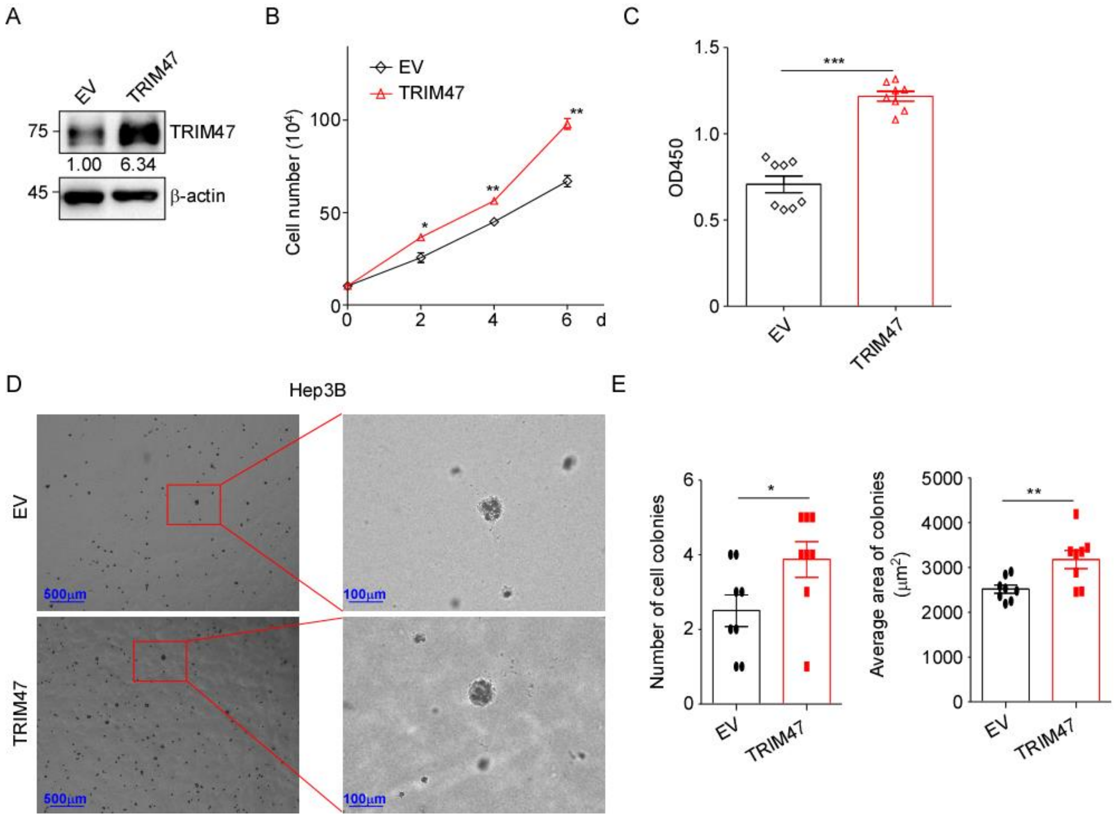

Figure S2: Overexpression of TRIM47 accelerated the proliferation of HCC cells.
A. Hep3B cells were infected with lentivirus to construct stable cell lines overexpressing TRIM47 or empty vector (EV). Western blot analysis of TRIM47 protein expression was used to confirm the construction of stable cells. **B**. The above stable cells were cultured using 1% FBS medium. At the indicated times, cells were trypsinized and counted using a cell counter. Data are presented as mean ±SD (n=3). **C.** The two stably cultured cell lines were inoculated into 96 culture plates, and the absorbance at 450 nm was detected by CCK-8 after 24 hours. **D.** Soft agar colony formation experiment. Ten thousand Hep3B cells infected with lentivirus were resuspended in full growth medium containing 0.35% agar (1mL/well) and inoculated on a growth medium containing 0.6% agar (1mL/well). Then the cells were cultured for about 20-25 days. Images were taken and colonies larger than 50 μM were calculated. **E.** The number of cell colonies was calculated in 8 randomly selected views, and the data were expressed as mean ± SD (n=8). The average area of colonies was then calculated in 8 randomly selected views. The data were expressed as mean ± SD (n=8). * P<0.05, ** P<0.01, *** P<0.001.

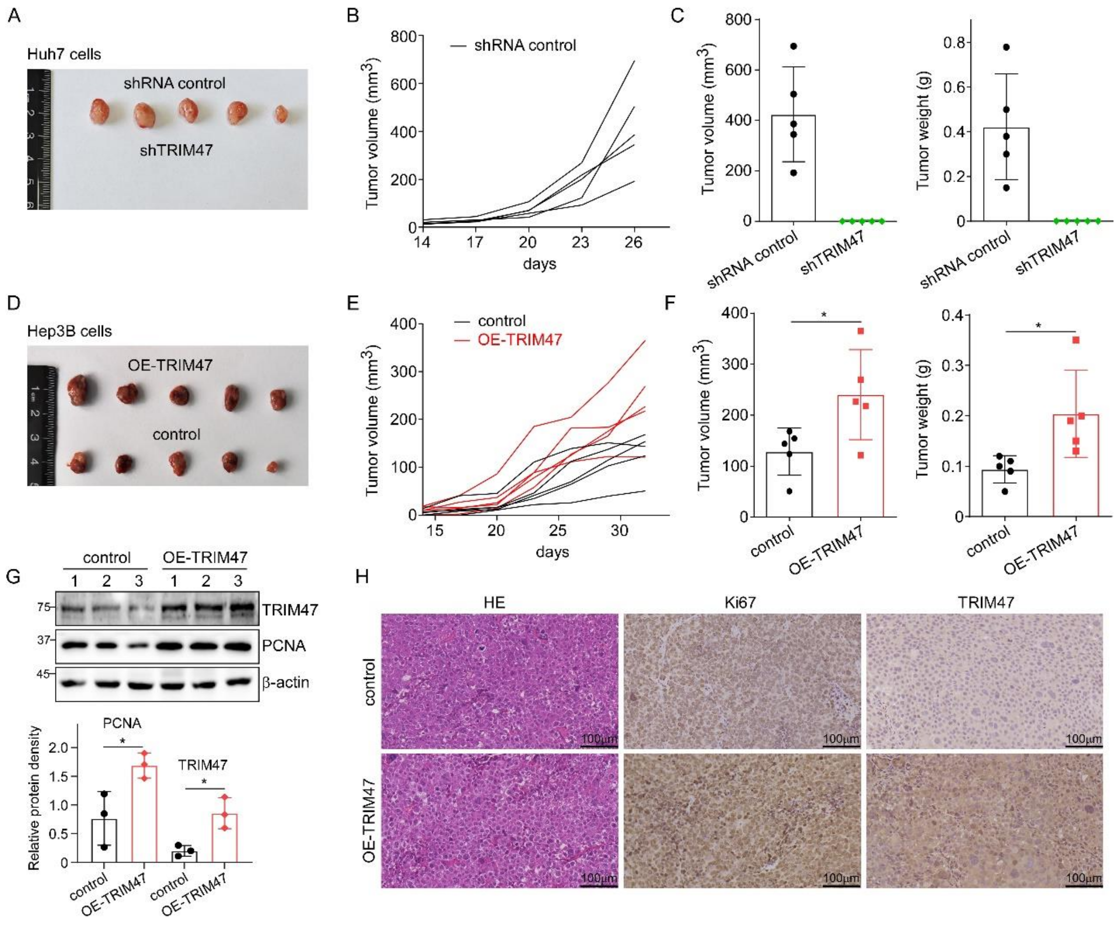

Figure S3: TRIM47 promoted HCC cell tumor growth in vivo.

A-C. TRIM47 knockdown inhibited the formation of tumors with Huh7 cells in vivo. Five male nude mice received bilateral subcutaneous injections of 5×10^6 TRIM47-knockdown stable cells or control cells in the dorsal cervical region. Fourteen days later, the tumor volume was measured every 3 days (**B**), and the mice were sacrificed on day 26. Tumors were isolated, measured and weighed. Images of the tumors are shown in **A**, while data on the tumor volume and weight are listed in **C**. Data are presented as the means ± SD (n=5). **D-F.** TRIM47 overexpression promoted Hep3B cell growth in a xenograft model. Five male nude mice received bilateral subcutaneous injections of 5×10^6 TRIM47-overexpressed stable cells or control cells in the dorsal cervical region. 14 days later, the tumor volume was measured every 3 days (**E**), and the mice were sacrificed on day 32. Tumors were isolated, measured and weighed. The images of the tumors are shown in **D**, while the tumor volume and weight data are listed in **F**. The data are presented as the means ± SD (n=5). * $P<0.05$. **G.** Upregulation of TRIM47 increased the protein expression of PCNA in vivo. Results are expressed as the mean ± SD (n=3). * $P<0.05$ by Student's t test. **H.** Tumors were isolated, fixed, and subjected to HE and IHC assays. TRIM47 promoted the expression of the proliferation marker Ki67.

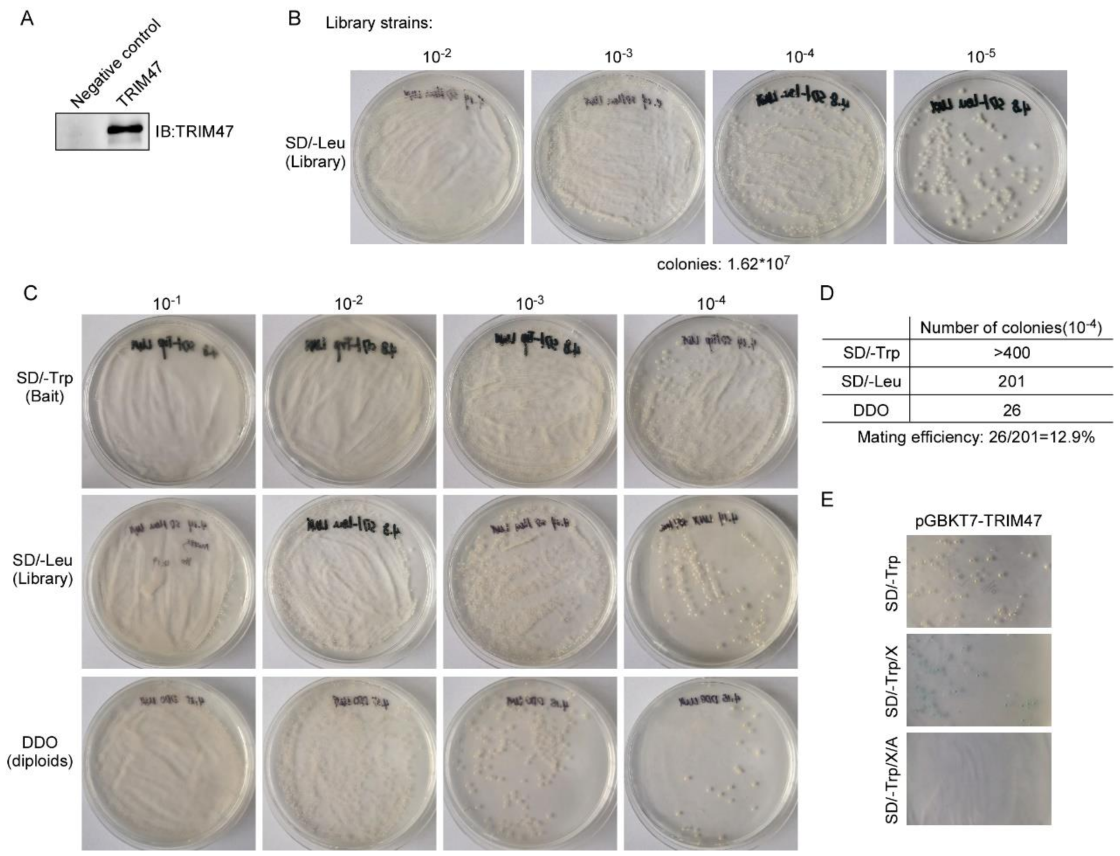

Figure S4: The screening of TRIM47 interacting proteins via yeast-two-hybrid system.
A. pGBKT7-TRIM47 was transformed into Y2HGold strain and the expression of TRIM47 in the yeast strains was detected by western blot. **B.** The titer of the library strains was carried out using 100 μL of 10^{-2}, 10^{-3}, 10^{-4} and 10^{-5} aliquots on SD/-Leu plates. Our 1 mL library strains contain 1.62×10^{7} cells, which is enough for mating. **C, D.** After mating for a period of 20-24h, 100 μL of 1/10, 1/100, 1/1000 and 1/10000 dilutions were spread onto the SD/-Trp, SD/-Leu and DDO plates, respectively (**C**). 5d later, the mating efficiency was calculated as: No. of diploids/No. of limiting partner×100%. Our mating efficiency was 12.9% (**D**), which is more than the average efficiency provided by the instructions (2-5%), indicating the successful screening procedure. **E.** Auto-activation assay. The transformation of TRIM47 into Y2HGold strain can't grow on the SD/-Trp/X/A plates, indicating that the bait protein didn't lead to auto-activation.

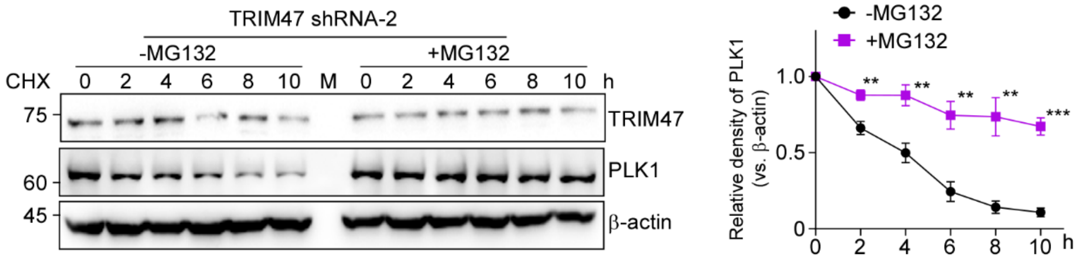

Figure S5: MG132 can reverse the effect of TRIM47 on the protein stability of PLK1. TRIM47 knockdown stable cells/control cells (Huh7) were treated with or without MG132 (10μM) and then incubated with CHX (100 μg/mL) for indicated times. Western blot was used to detect the protein expression of TRIM47 and PLK1 in the above cells, and ImageJ software was used for gray quantification analysis. The data were expressed as mean ± SD (n=3). ** P<0.01, *** P<0.001.

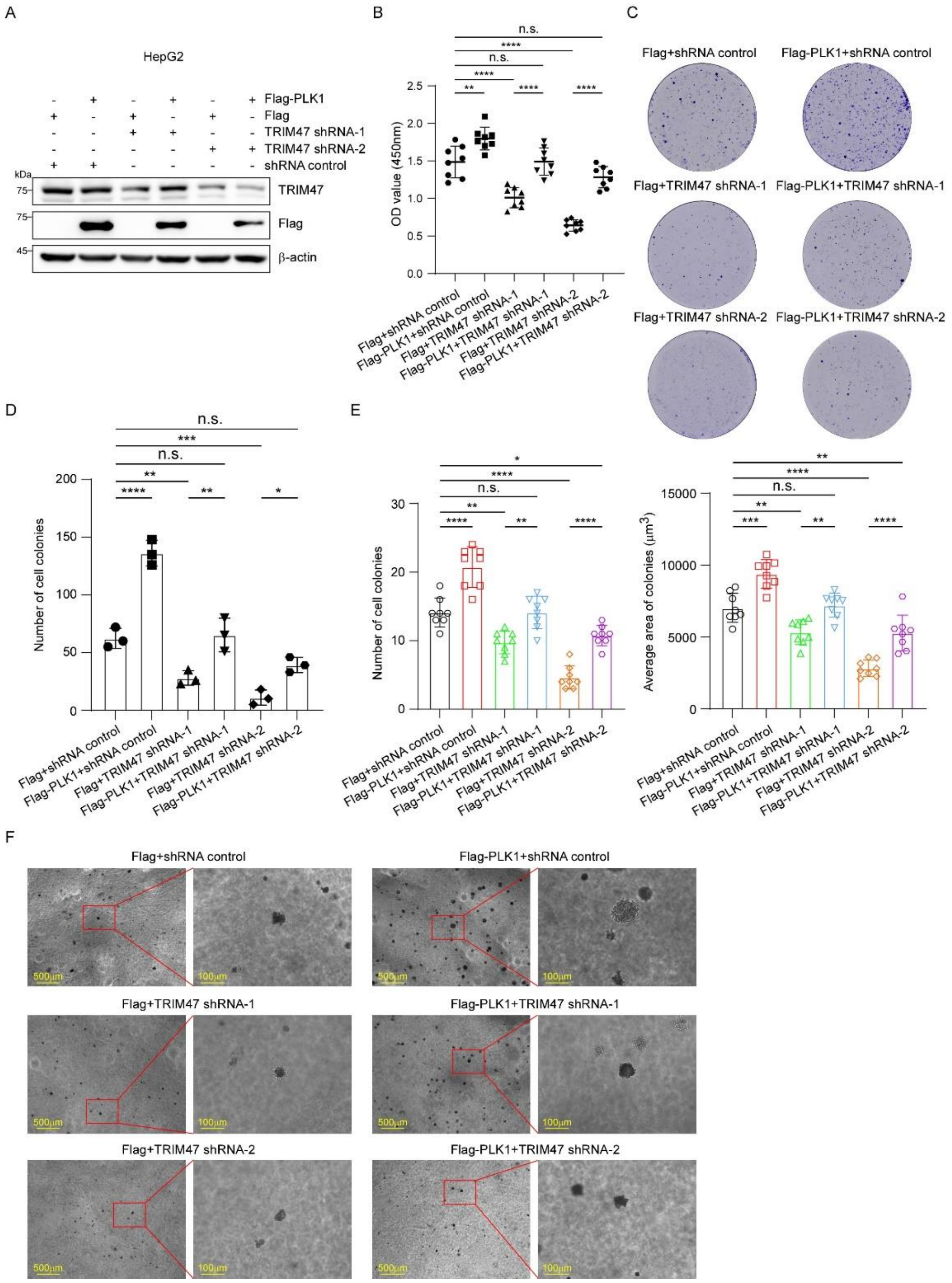

Figure S6: The effect of TRIM47 on HepG2 cell proliferation is dependent on PLK1.

A. Western blot analysis of 6 groups of co-transfected HepG2 cells (Flag (empty vector) + shRNA control, Flag-PLK1 + shRNA control, Flag + TRIM47 shRNA-1, Flag-PLK1 + TRIM47 shRNA-1, Flag + TRIM47 shRNA-2, Flag-PLK1 + TRIM47 shRNA-2). **B.** CCK-8 assay. The above cells were co-transfected with the indicated vectors. After 48 hours, the cells were plated into 96-well plates and then subjected to CCK-8 analysis (n=8). **C.** Colony formation assay. A total of 5×10^3 cells from each group were plated in 6-well plates containing 5% FBS medium. After 10-14 days, the cells were fixed and stained with 2% crystal violet. **D.** The number of cell colonies was calculated and statistically analyzed (n=3). **E-F.** Soft agar colony formation assay. 6 groups of HepG2 cells were seeded in 6-well plates in full growth medium (10% FBS) containing 0.35% agar (1 mL per well) on top of a layer of growth medium containing 0.6% agar (1 mL per well). After 20-25 days, the cells were imaged, and colonies larger than 50 μm were counted, after which the area was calculated (n=8). n.s. $P>0.05$, * $P<0.05$, ** $P<0.01$, *** $P<0.001$, **** $P<0.0001$ by one-way ANOVA followed by Tukey's test.

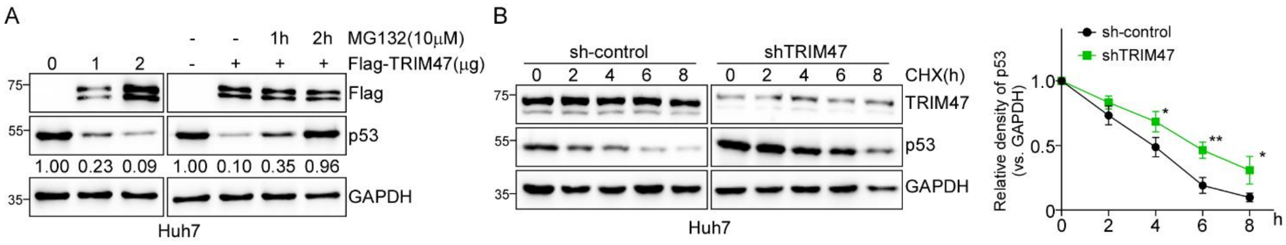

Figure S7: TRIM47 regulated the protein stability of p53 dependent on UPS.

A. Overexpression of TRIM47 suppressed endogenous protein levels of p53 in Huh7 cells dose-dependently. MG132 can reverse the protein decline of p53. **B.** Knockdown of TRIM47 prolonged the half-life of endogenous p53 in hepatic cancer cells. The data were expressed as mean ± SD (n=3). * P<0.05, ** P<0.01.

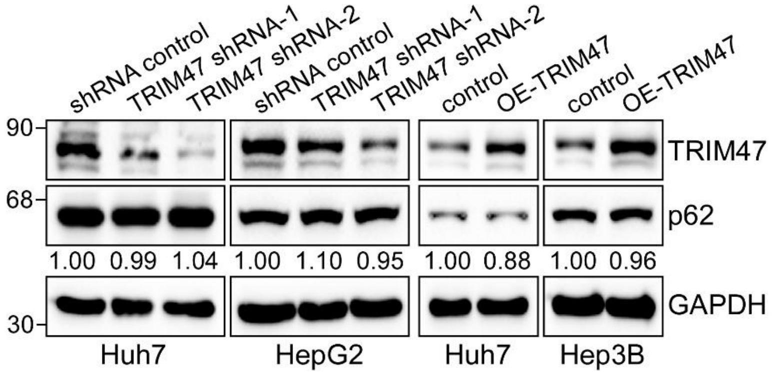

Figure S8: The expression levels of p62 protein in HCC stable cell lines.

Whole-cell lysates were prepared from TRIM47-knockdown Huh7 and HepG2 cells, TRIM47-overexpressed Huh7 and Hep3B cells, along with their corresponding control cells. p62 protein levels were subsequently analyzed by western blot.

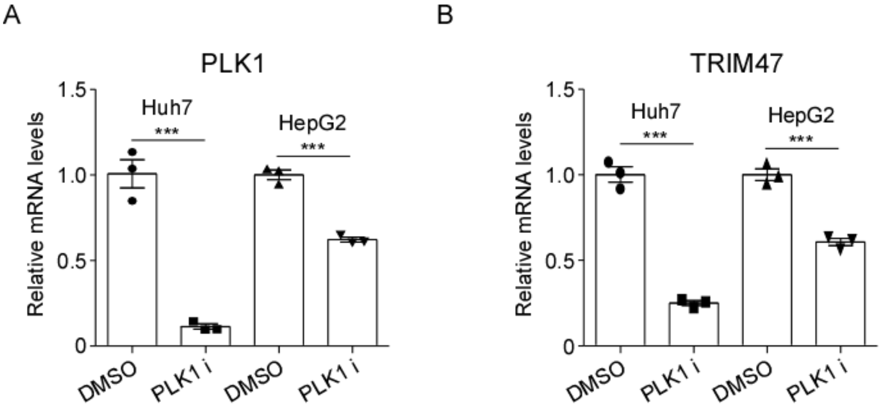

Figure S9: The effect of PLK1 inhibitor on the mRNA level of TRIM47 in HCC cells.

Quantitative real-time PCR was used to detect the mRNA expression level of PLK1(**A**) and TRIM47(**B**) in Huh7 and HepG2 cells treated with PLK1 inhibitor. The data were expressed as mean ± SD (n=3). *** P<0.001.

Table S1: Primer sequences used in RT-qPCR experiment.

Primer names	Sequence
TRIM47 Forward Primer	5'-CAGTCCAAAGTCCTGAGCG-3'
TRIM47 Reverse Primer	5'-GCTACGGCTGCACTCTTGAT-3'
PLK1 Forward Primer	5'-CCATCACCTGCCTGACCATT-3'
PLK1 Reverse Primer	5'-GGGGGTTCTCCAAGCCTTTA-3'
β-ACTIN Forward Primer	5'-ACGTTGCTATCCAGGCTGTG-3'
β-ACTIN Reverse Primer	5'-GAGGGCATACCCCTCGTAGA-3'

Figure S10: Full unedited pictures for WB results

Figure 1A

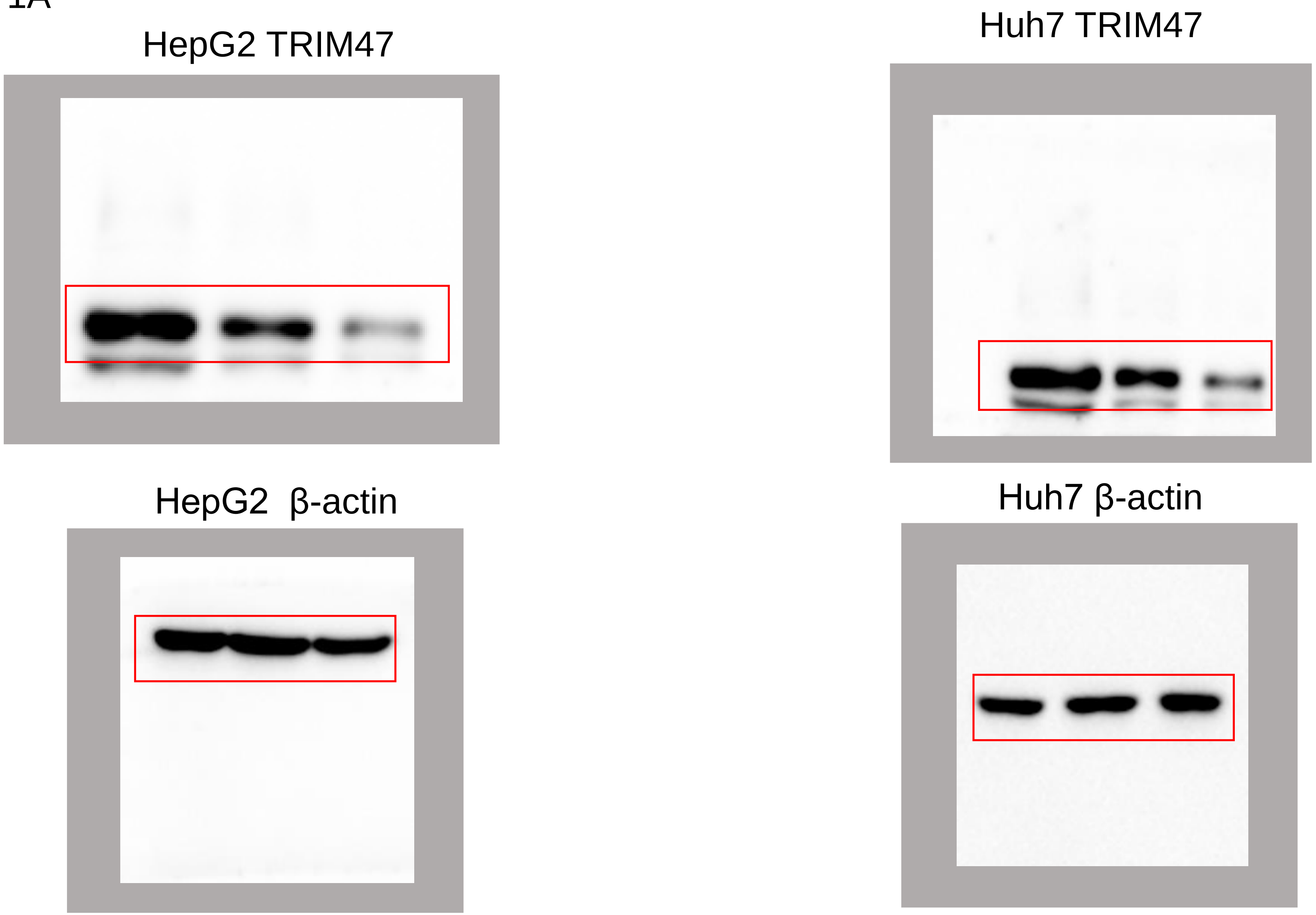

Figure 2D

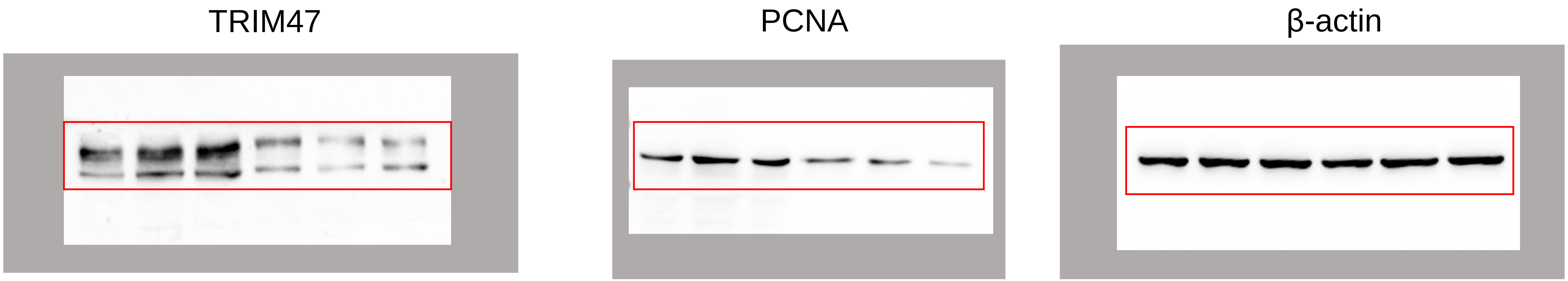

Figure 2I

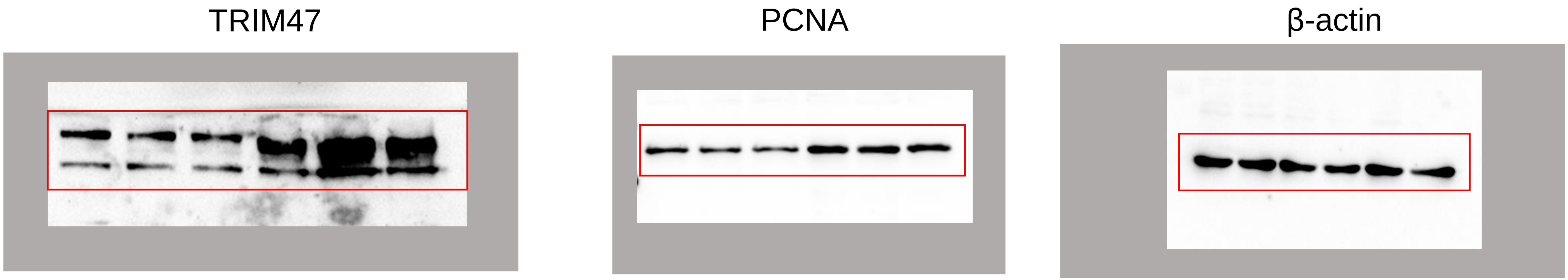

Figure 3C

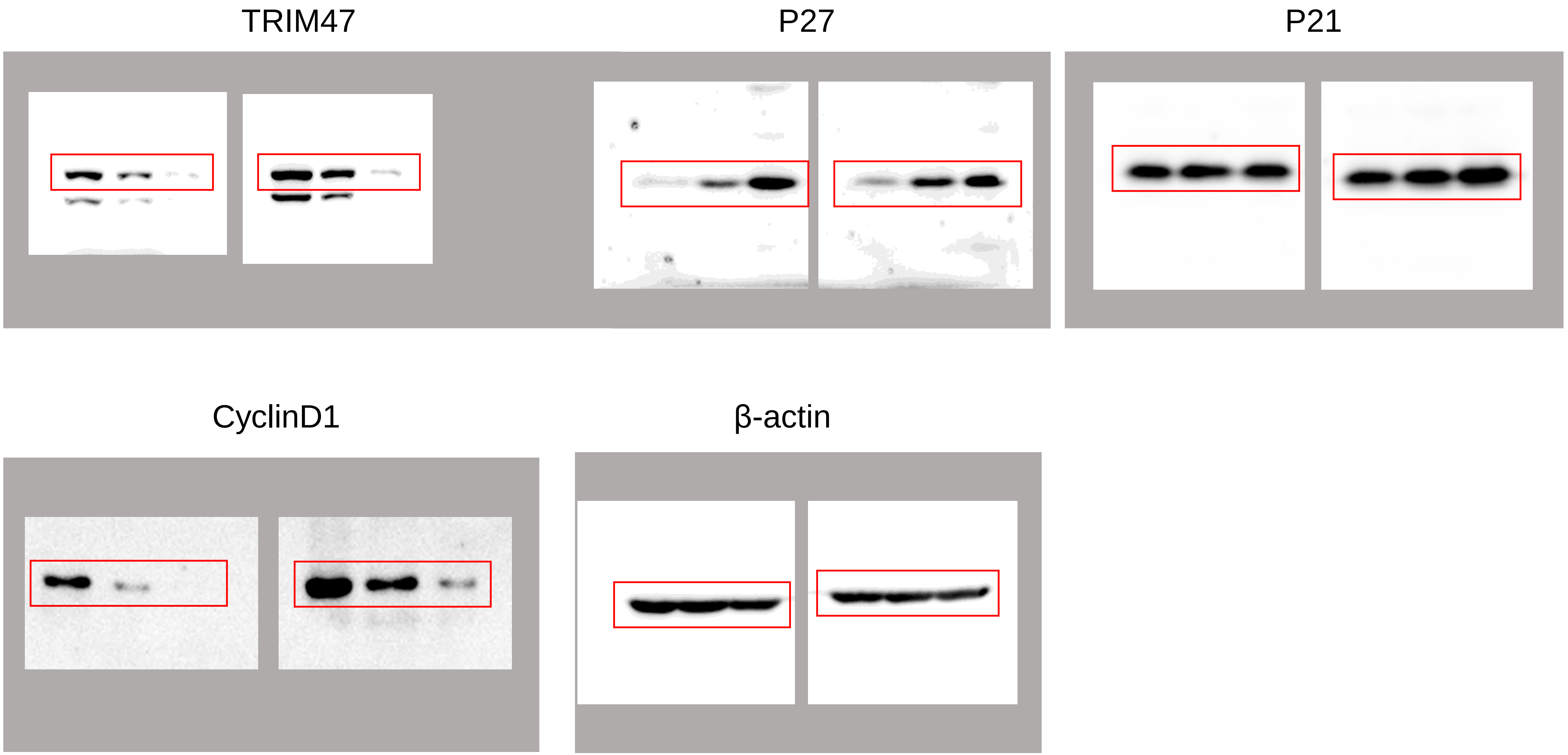

Figure 3F

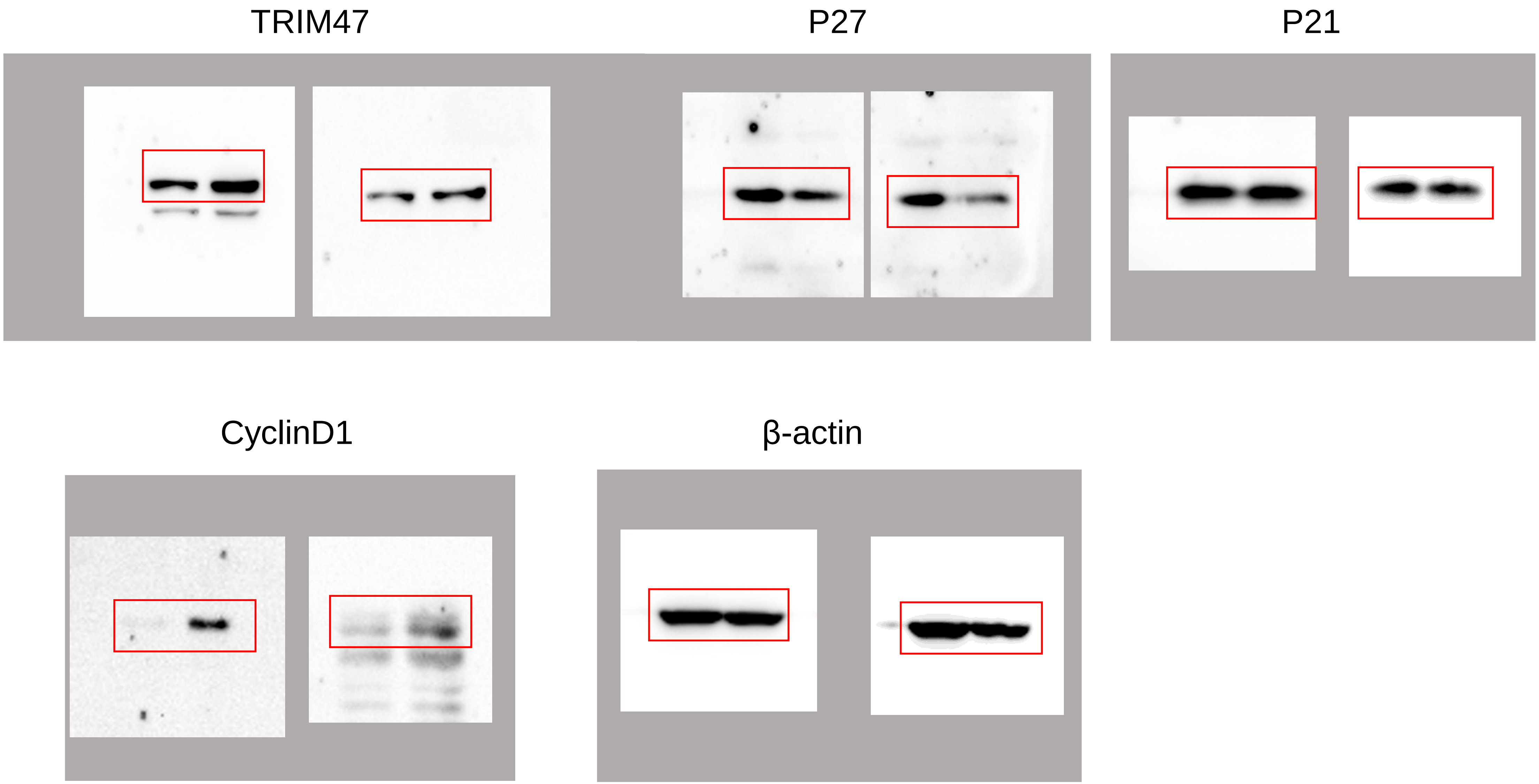

Figure 3G

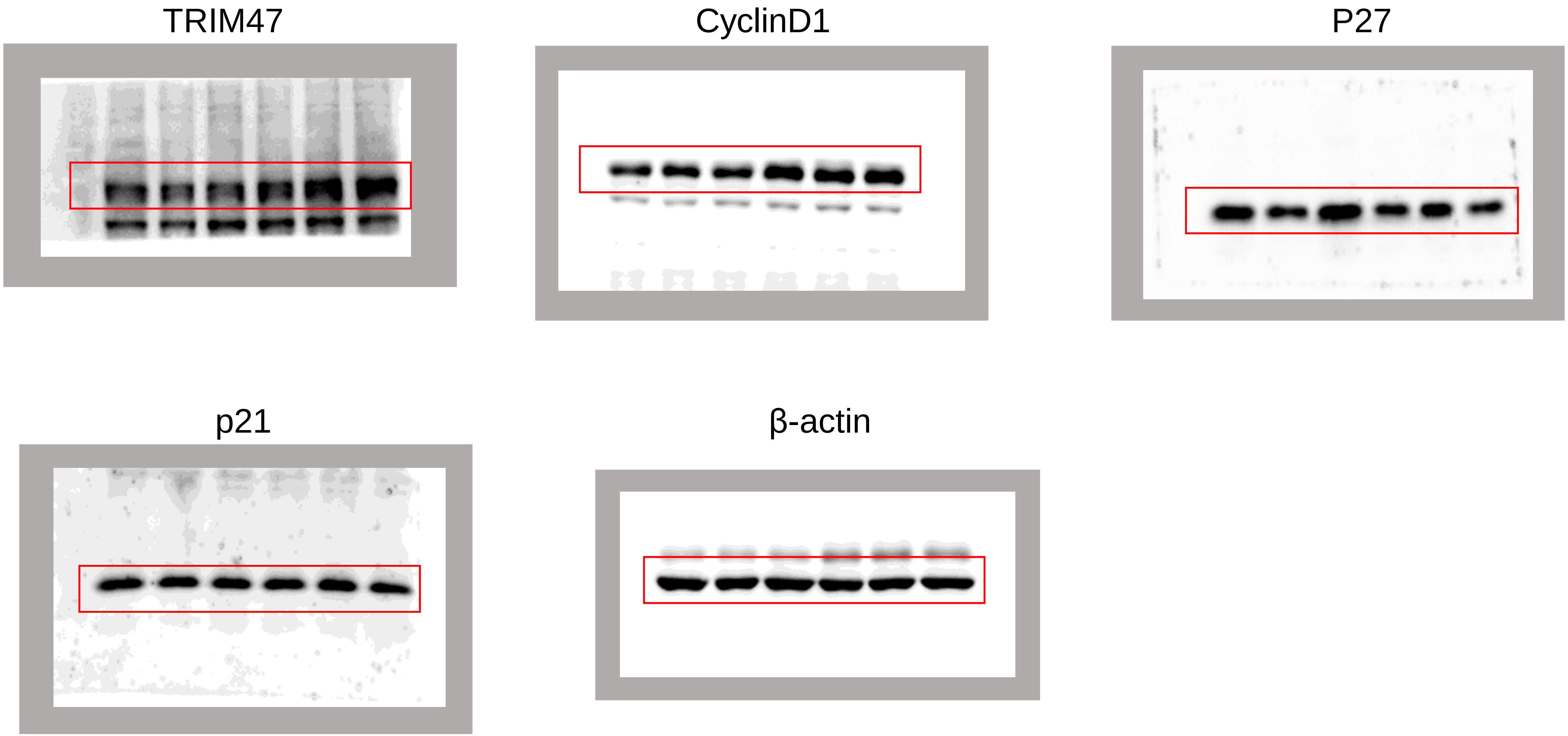

Figure 3I

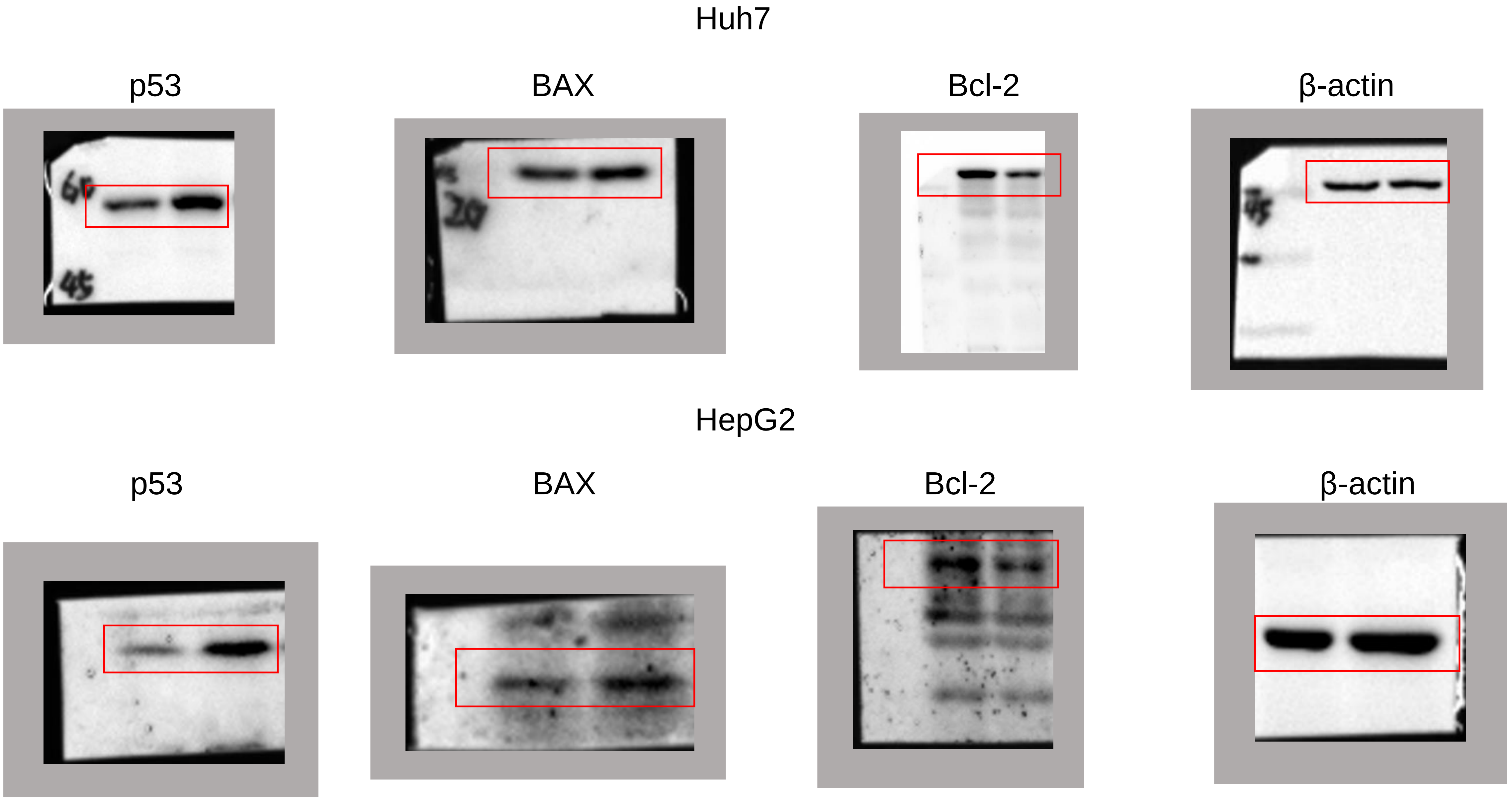

Figure 3K

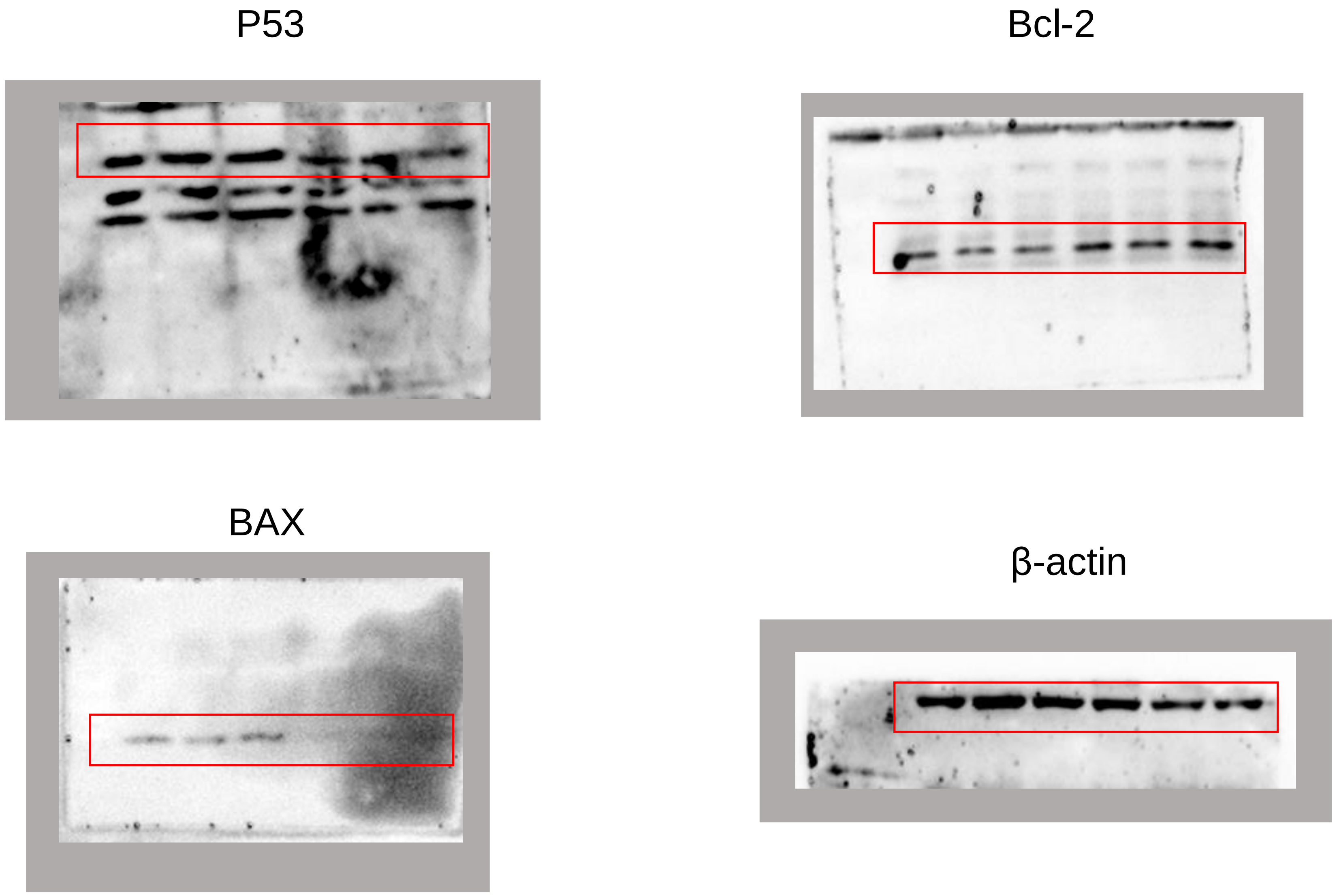

Figure 4C

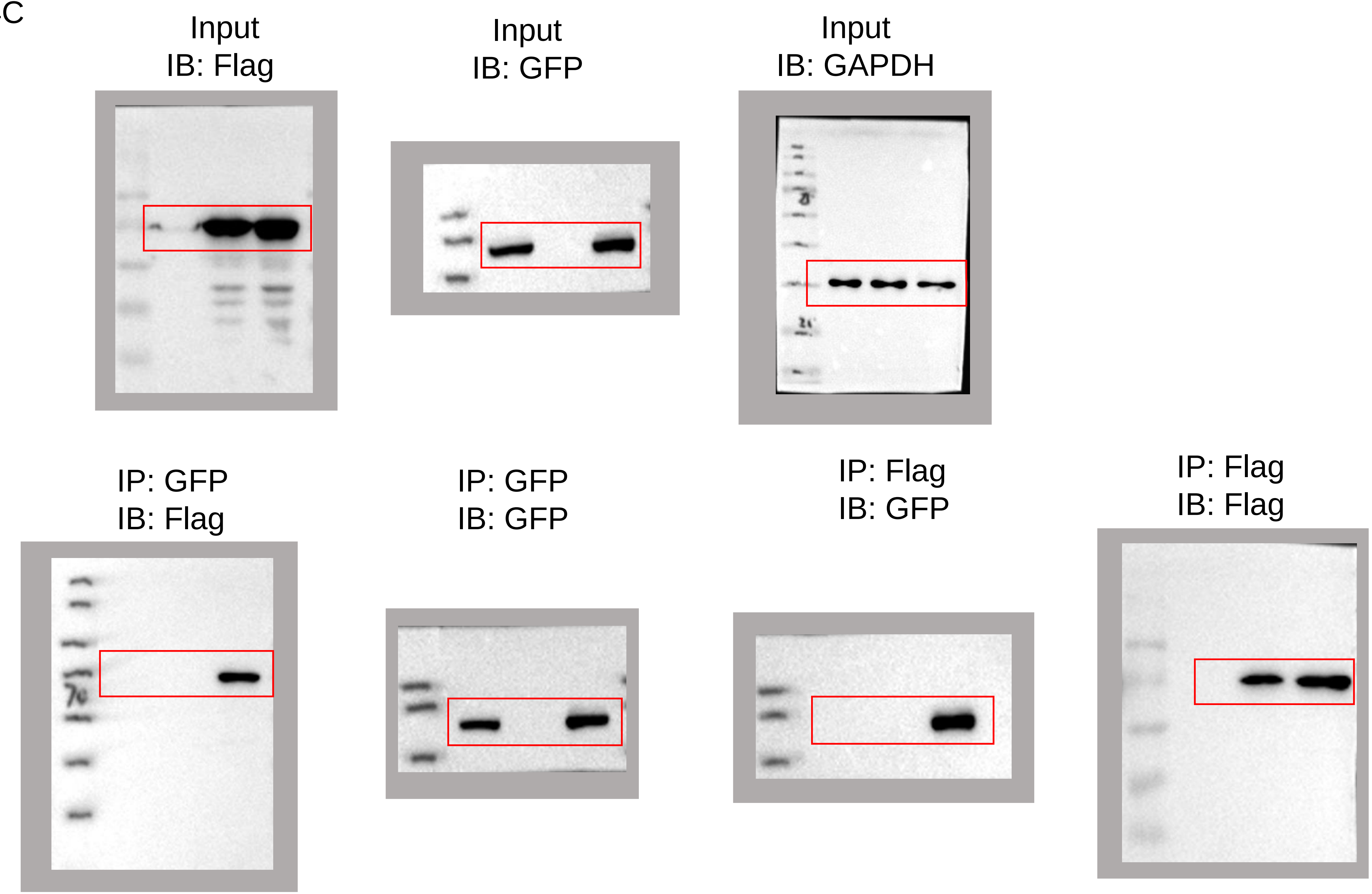
Input
IB: Flag
Input
IB: GFP
Input
IB: GAPDH
IP: GFP
IB: Flag
IP: GFP
IB: GFP
IP: Flag
IB: GFP
IP: Flag
IB: Flag

Figure 4D

PLK1

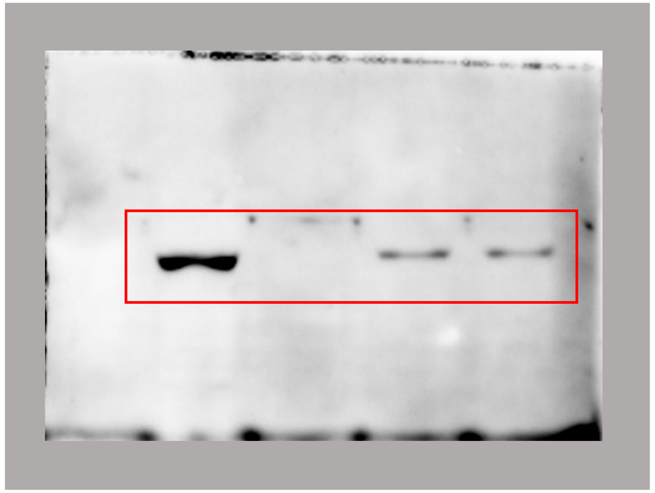

TRIM47

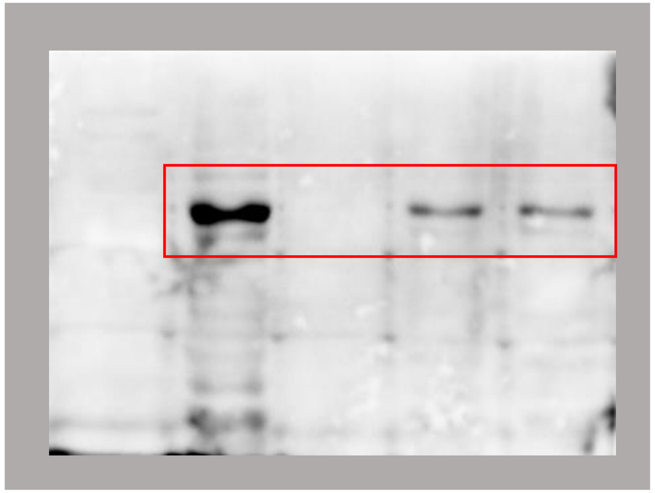

Figure 4E

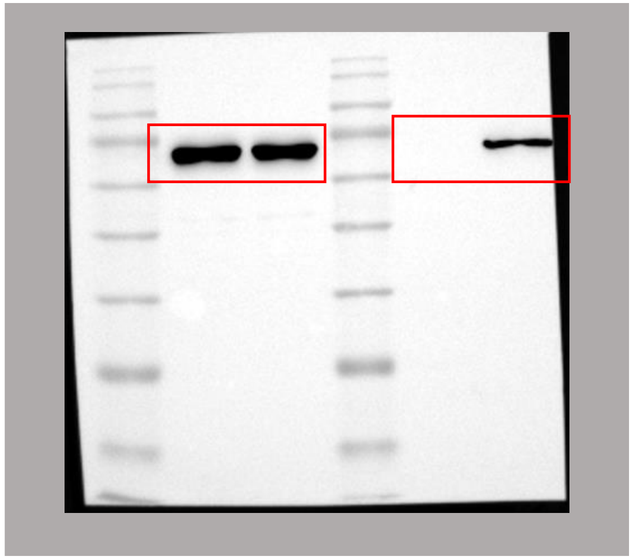
Input
IB: Flag
GST pull-down
IB:Flag

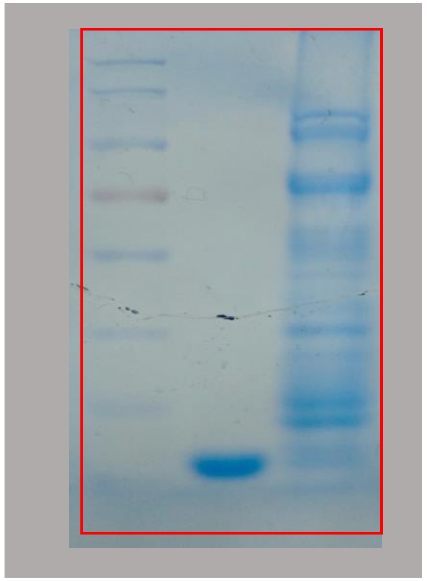
coomassie brilliant blue

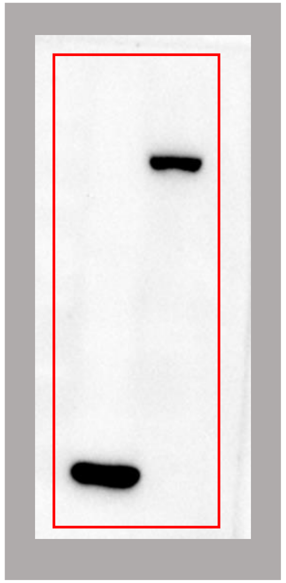
GST pull-down
IB:GST

Figure 4G

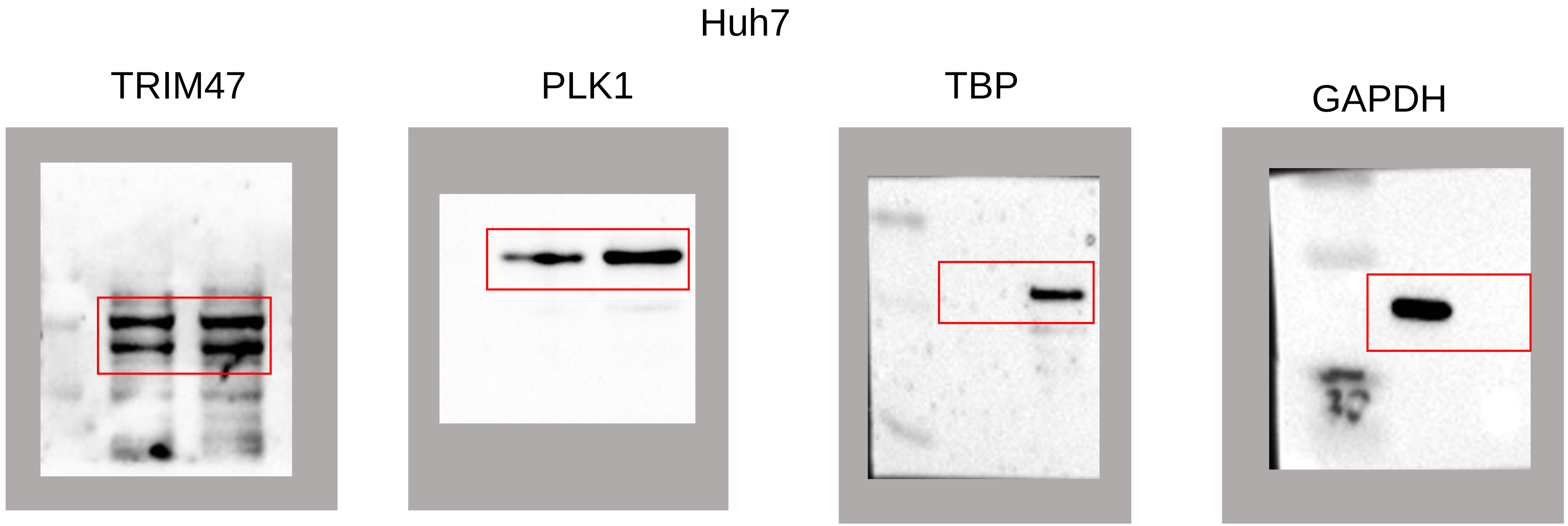

Figure 4I

Input
IB: GFP

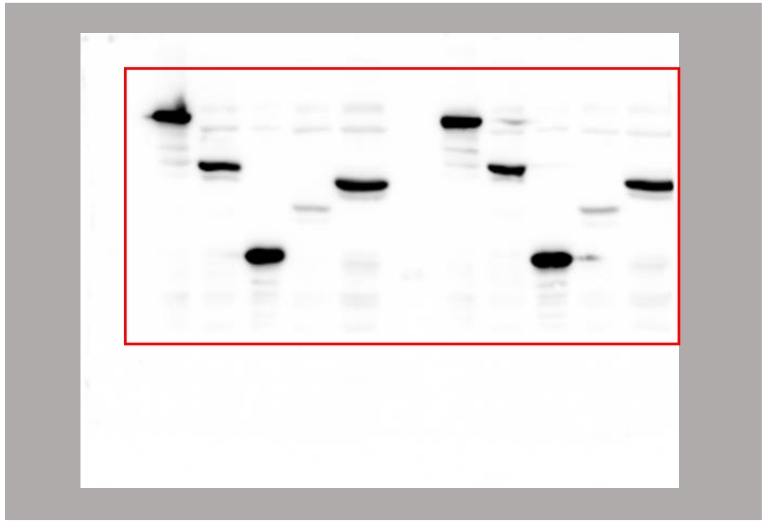

GST pull-down
IB: GFP

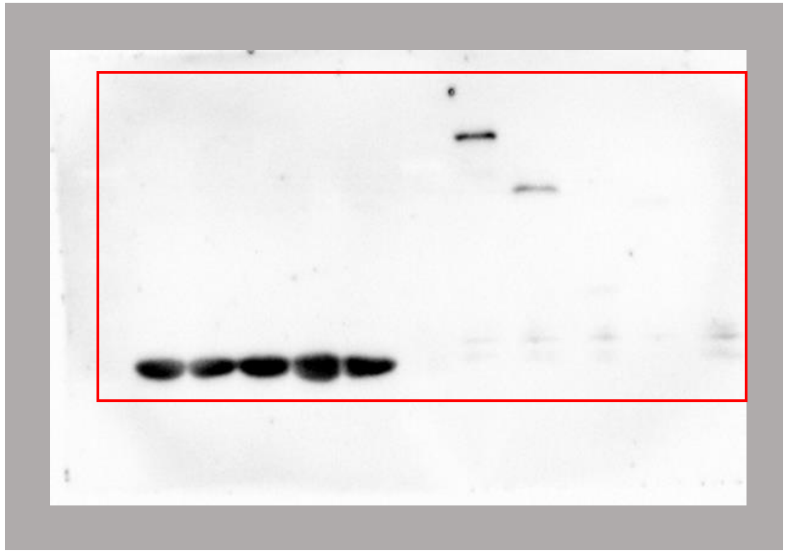

coomassie brilliant blue

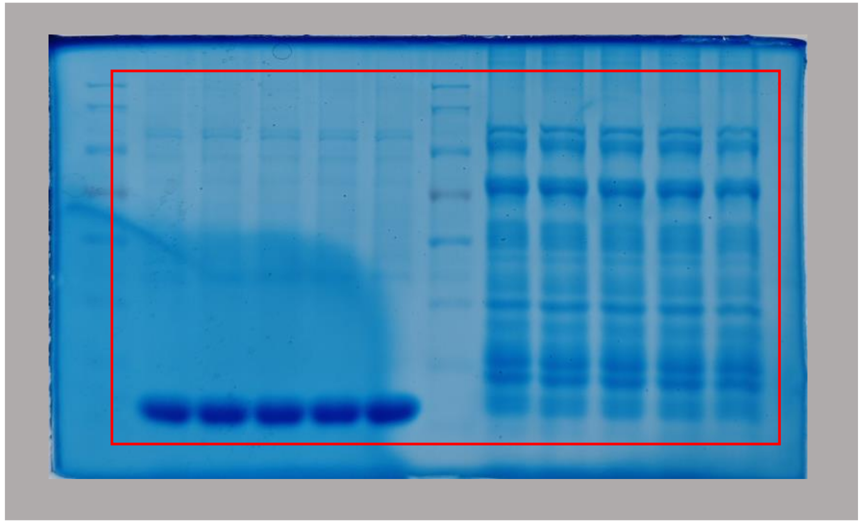

Figure 4J

Input
IB: Flag

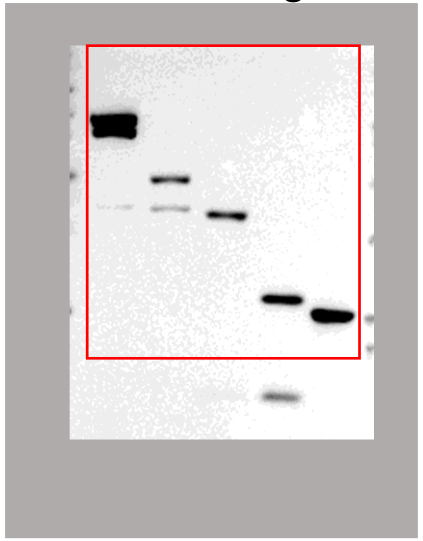

Input
IB: GFP

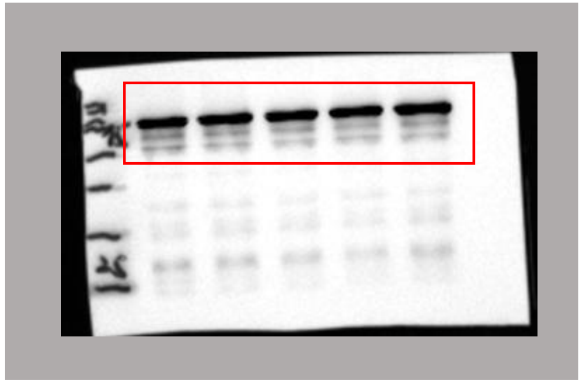

IP: GFP
IB: Flag

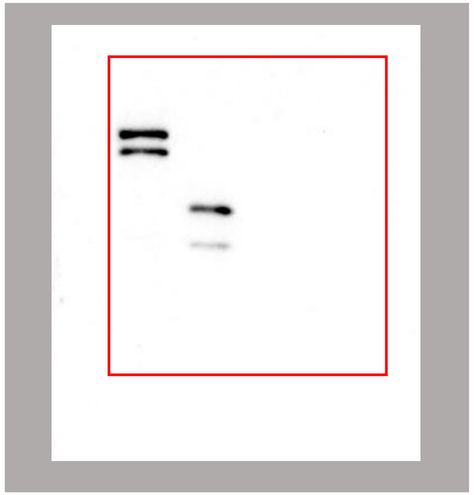

IP: GFP
IB: GFP

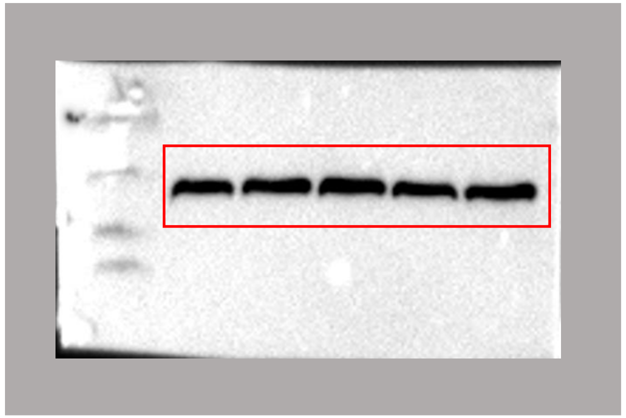

Figure 4L

Input
IB: GFP

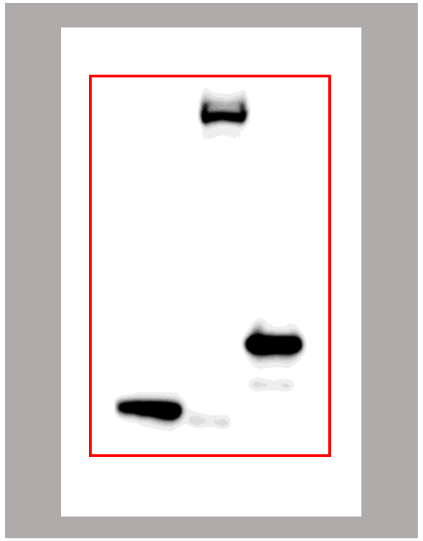

Input
IB: Flag

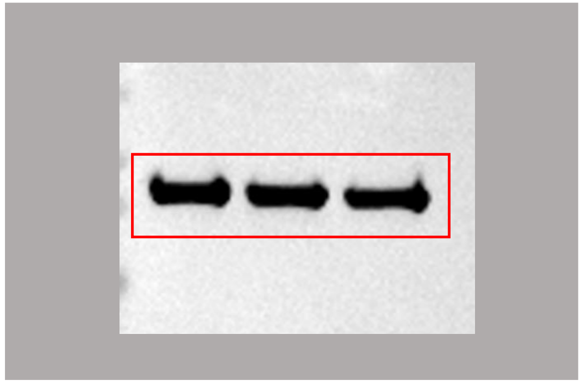

IP: Flag
IB: GFP

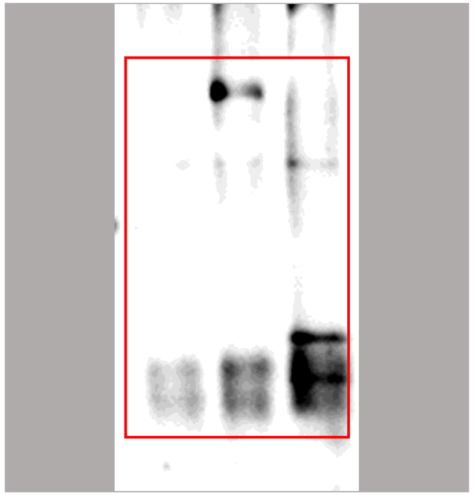

IP: Flag
IB: Flag

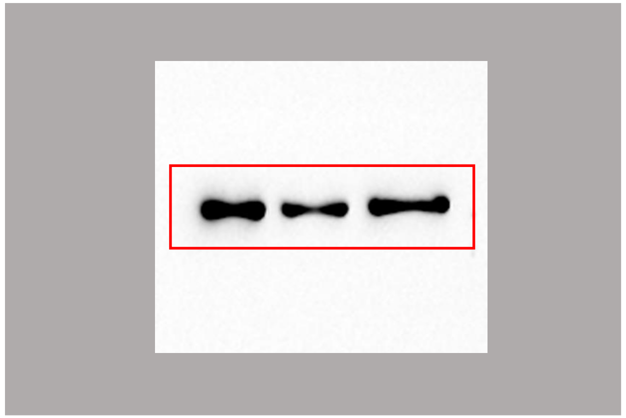

Figure 5A

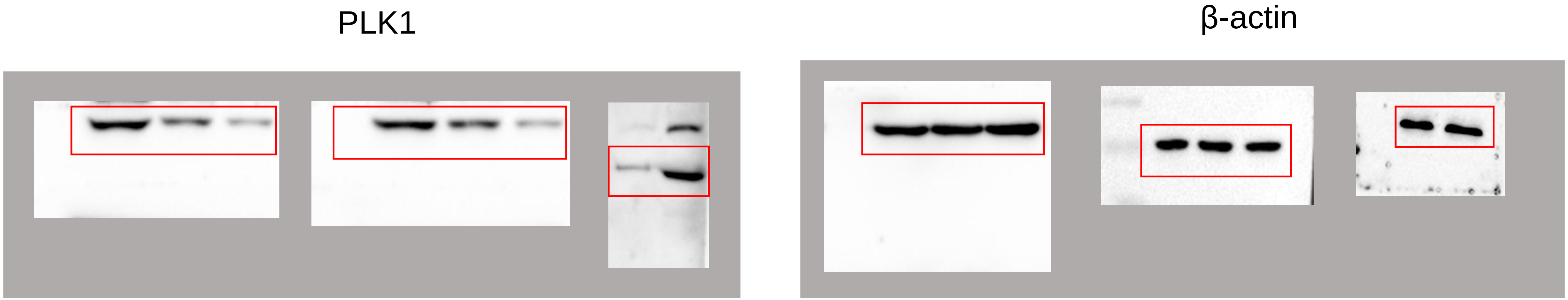

Figure 5B

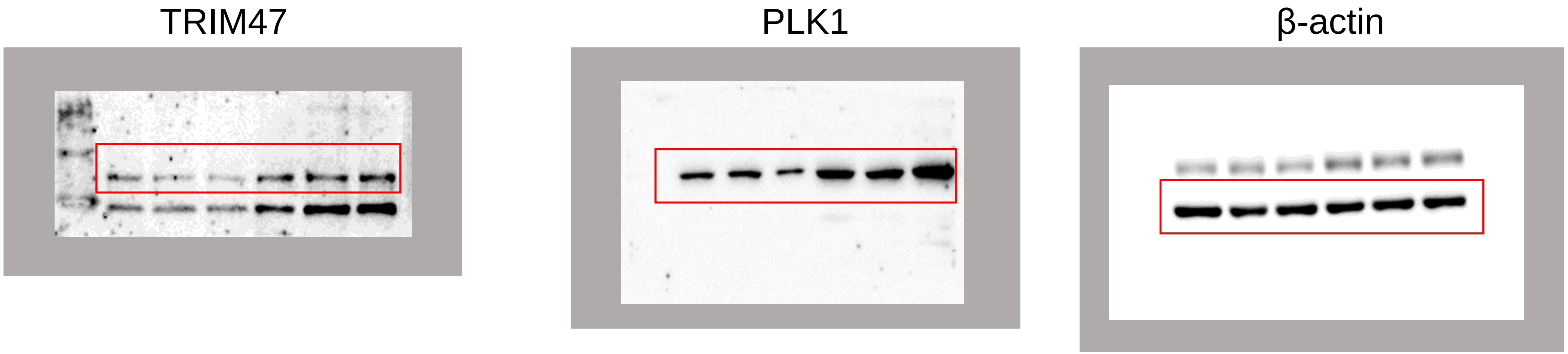

Figure 5D

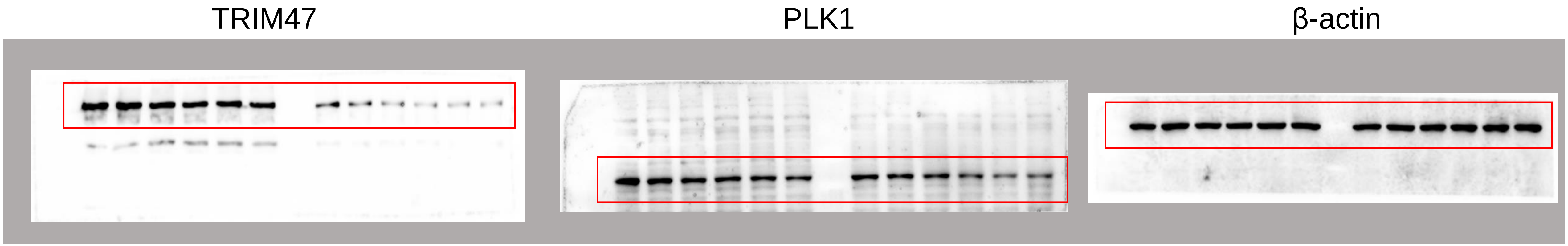

Figure 5E

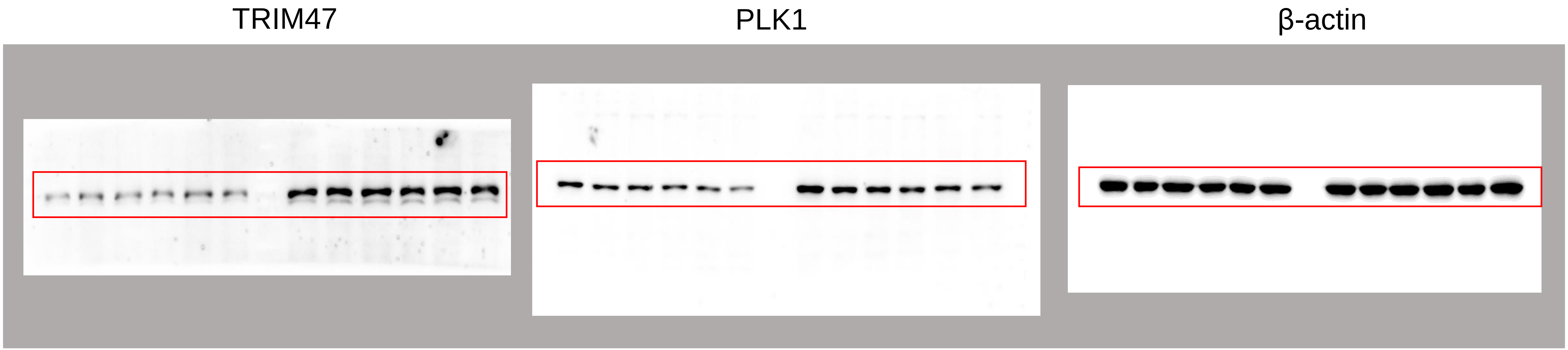

Figure 5F

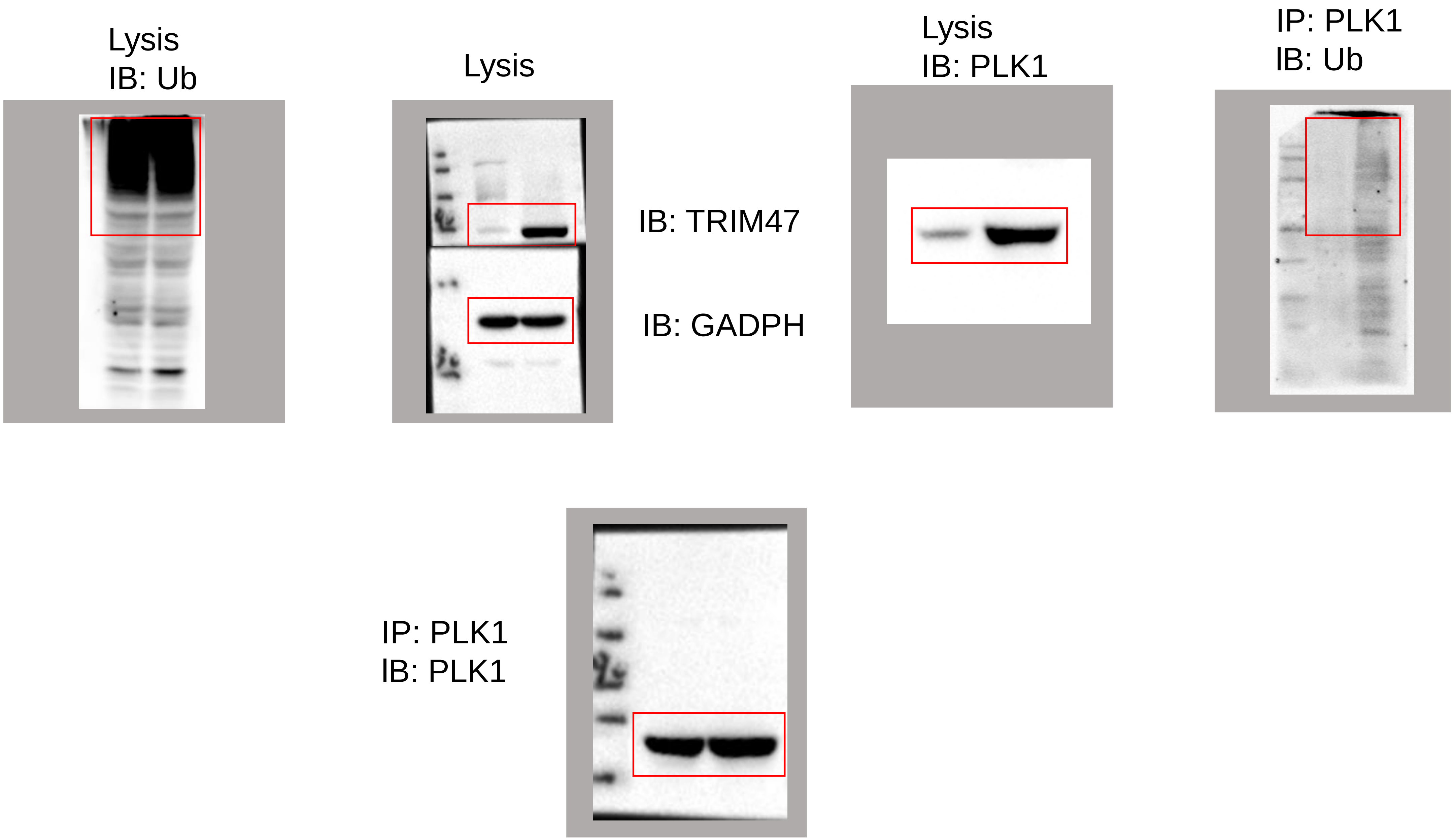
Lysis
IB: Ub
Lysis
IB: TRIM47
IB: GADPH
Lysis
IB: PLK1
IP: PLK1
IB: Ub
IP: PLK1
IB: PLK1

Figure 5G

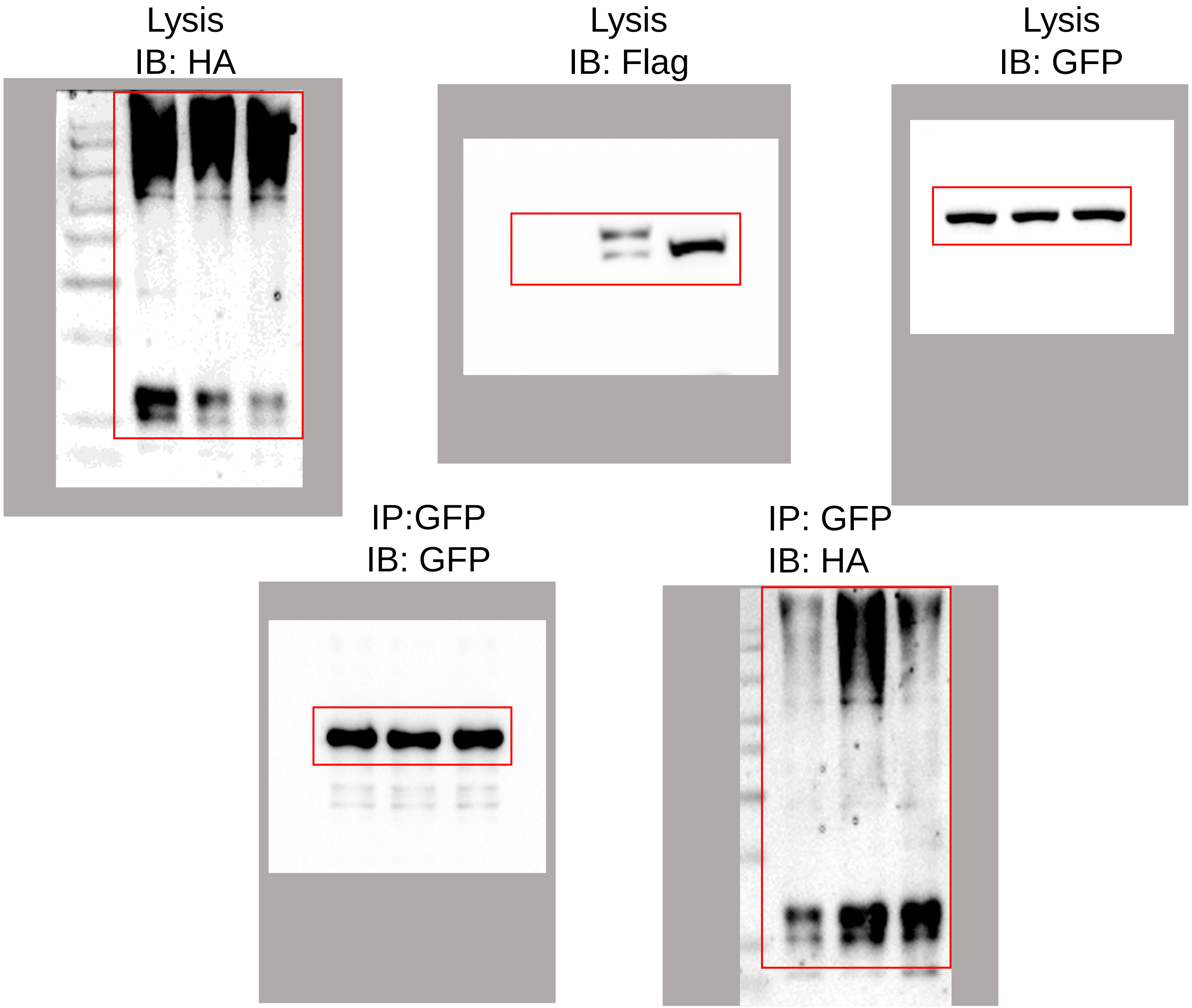

Figure 5H

Lysis
IB: HA

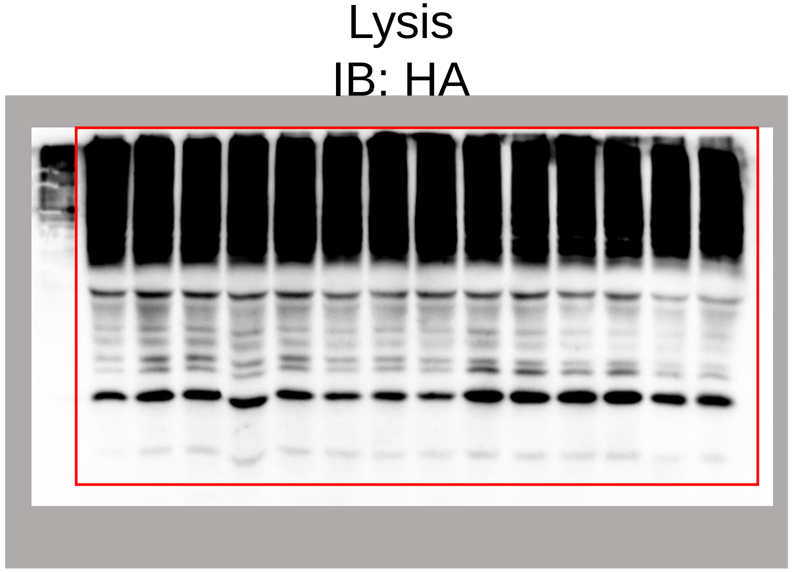

Lysis
IB: GFP

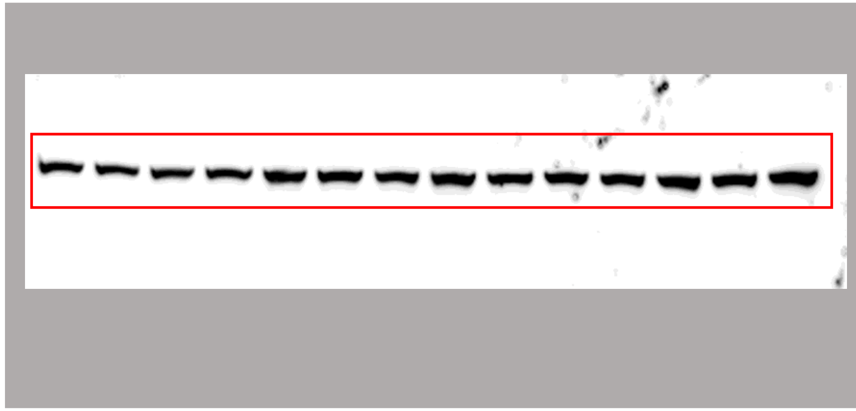

Lysis
IB: Flag

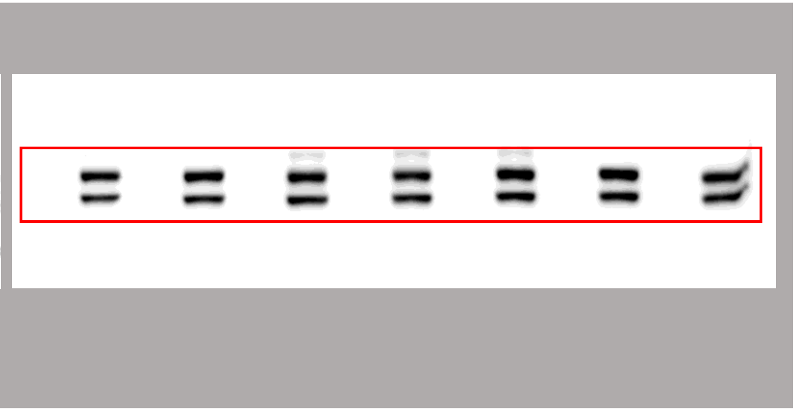

IP: GFP
IB: HA

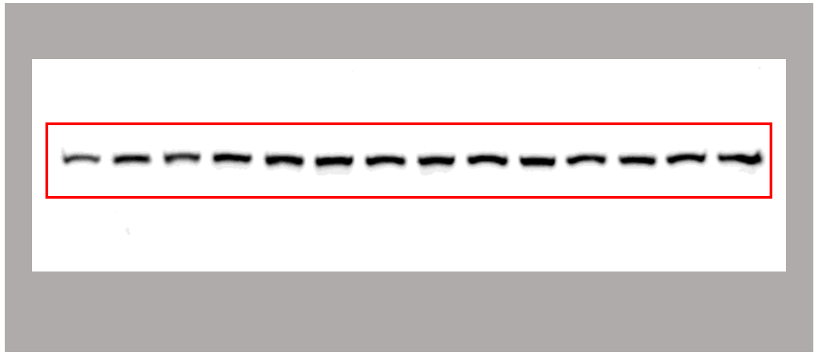

IP: GFP
IB: HA

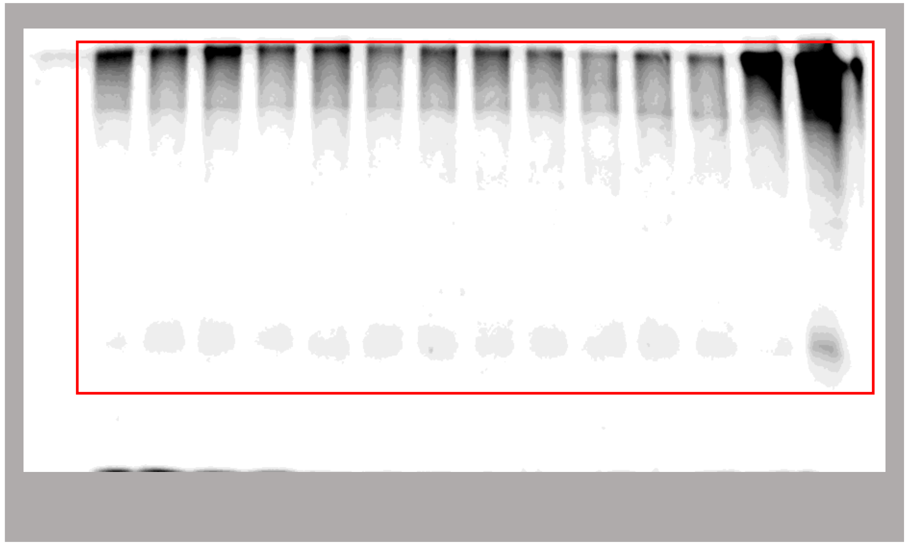

Figure 5I

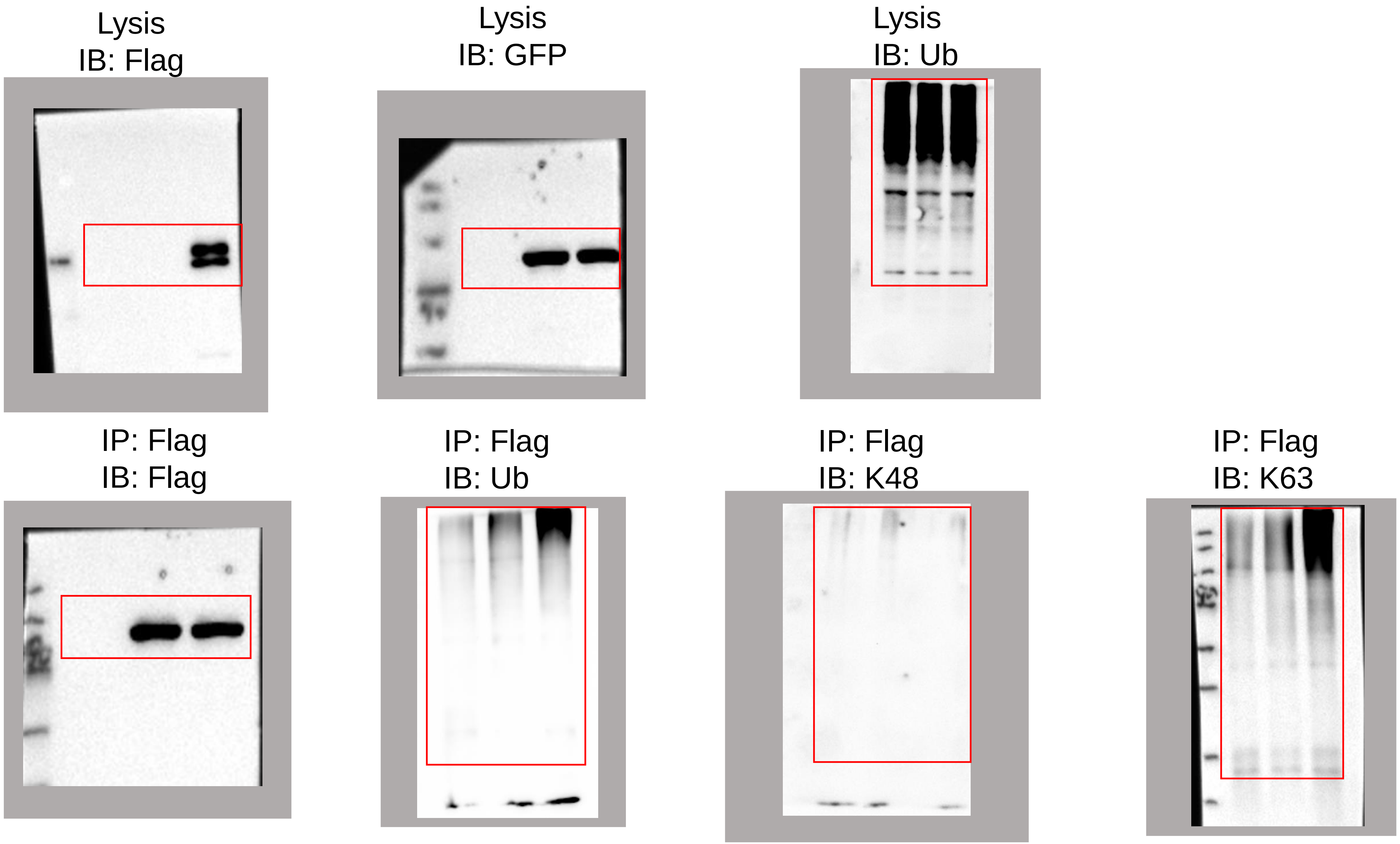
Lysis
IB: Flag
Lysis
IB: GFP
Lysis
IB: Ub
IP: Flag
IB: Flag
IP: Flag
IB: Ub
IP: Flag
IB: K48
IP: Flag
IB: K63

Figure 5J

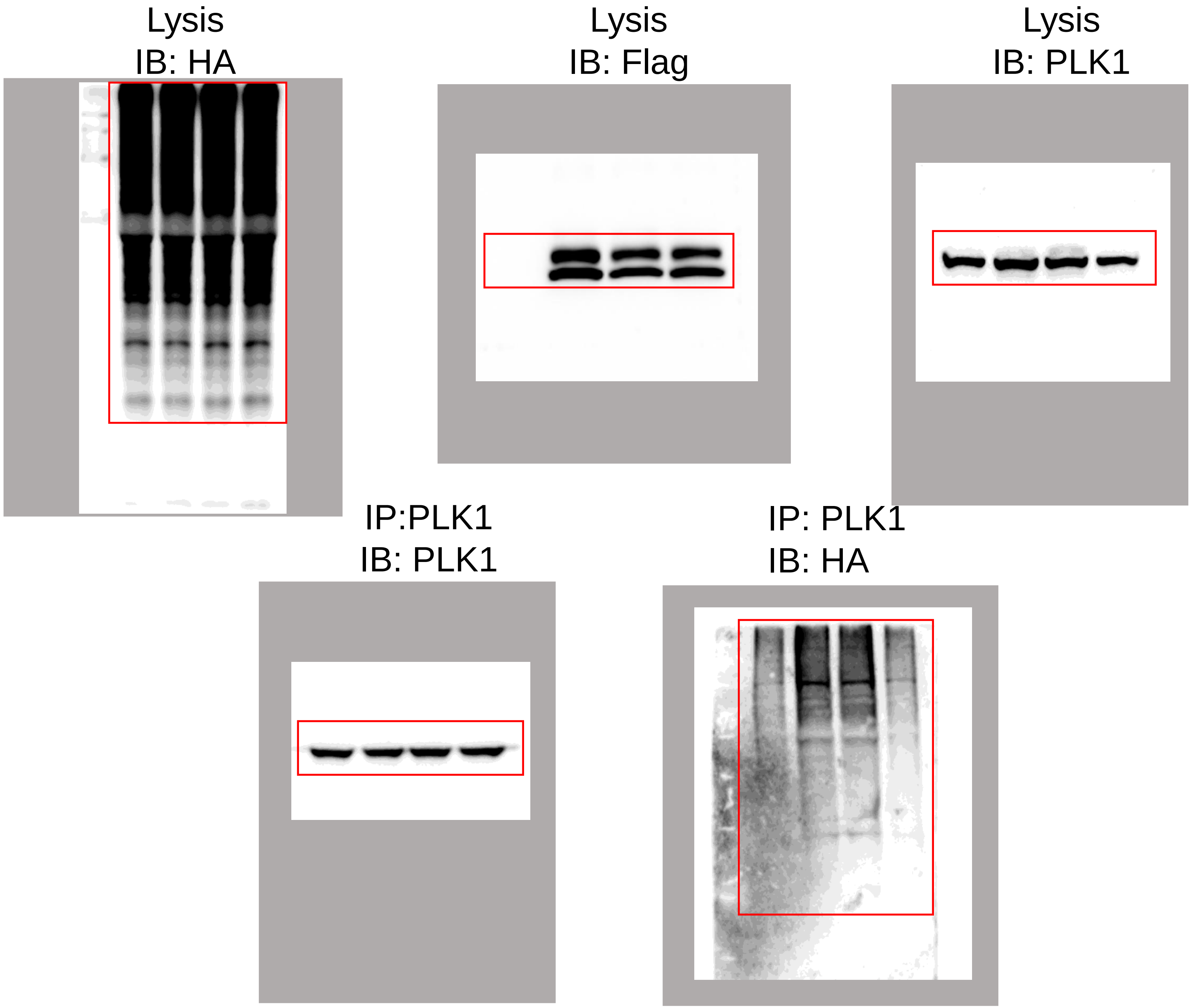

Lysis
IB: HA
Lysis
IB: Flag
Lysis
IB: PLK1
IP:PLK1
IB: PLK1
IP: PLK1
IB: HA

Figure 6A

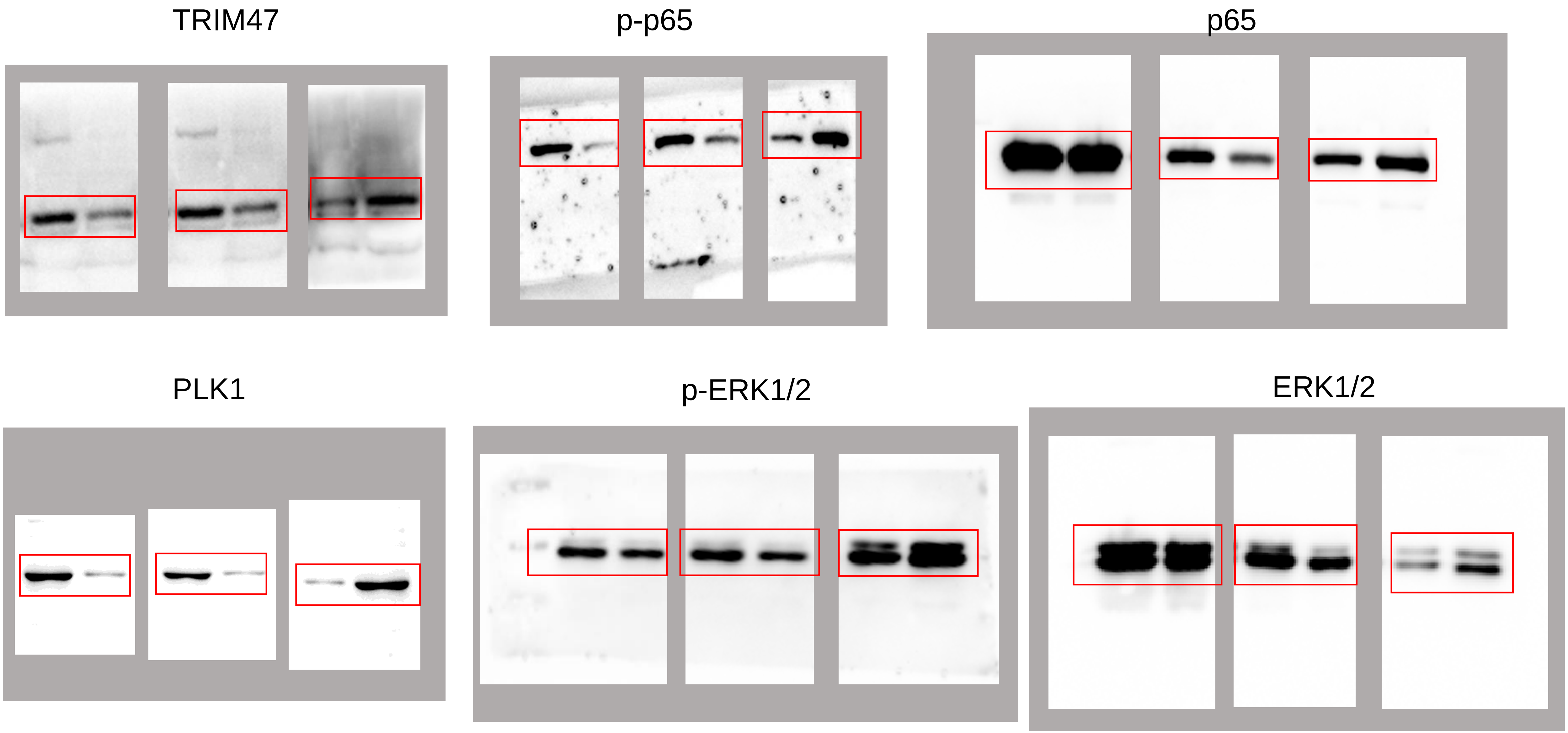

Figure 6A

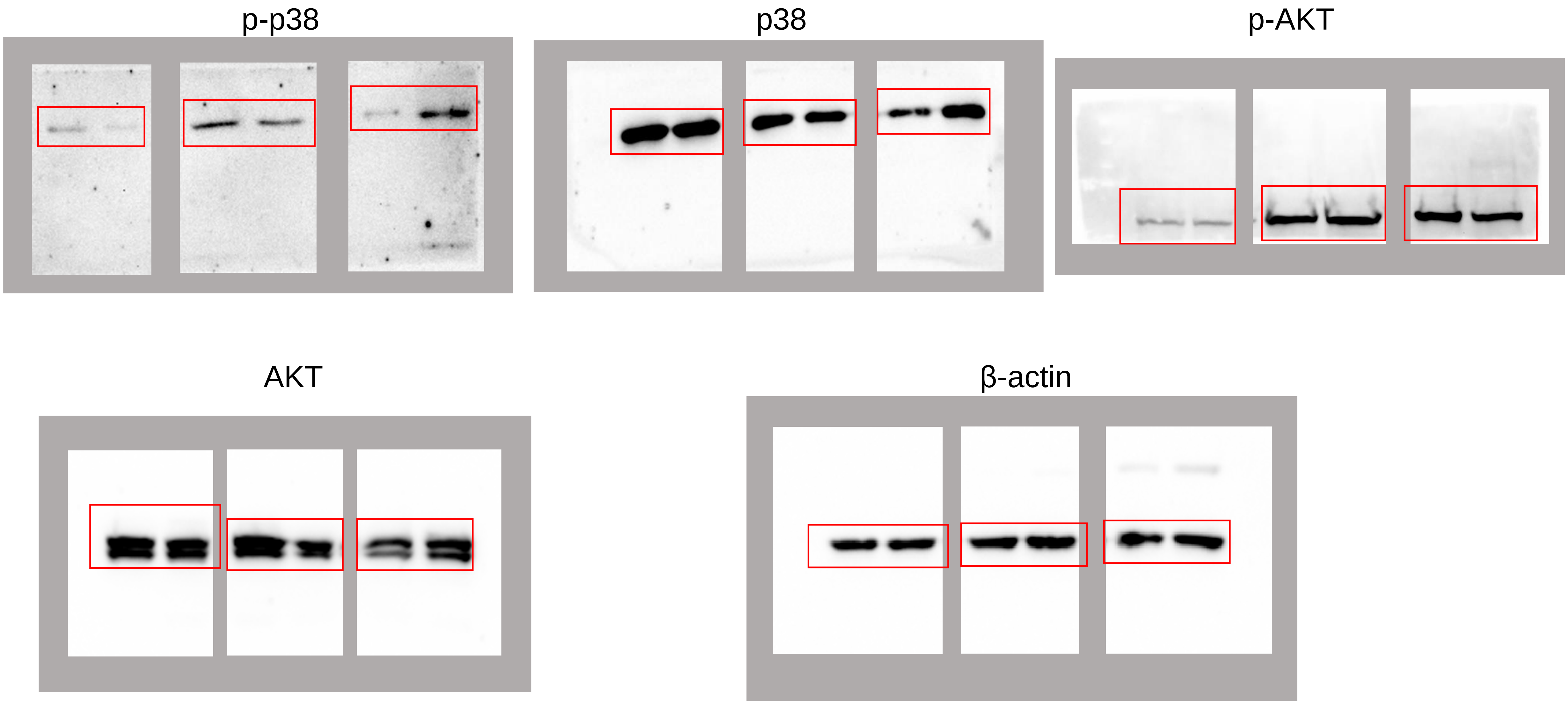

Figure 6C

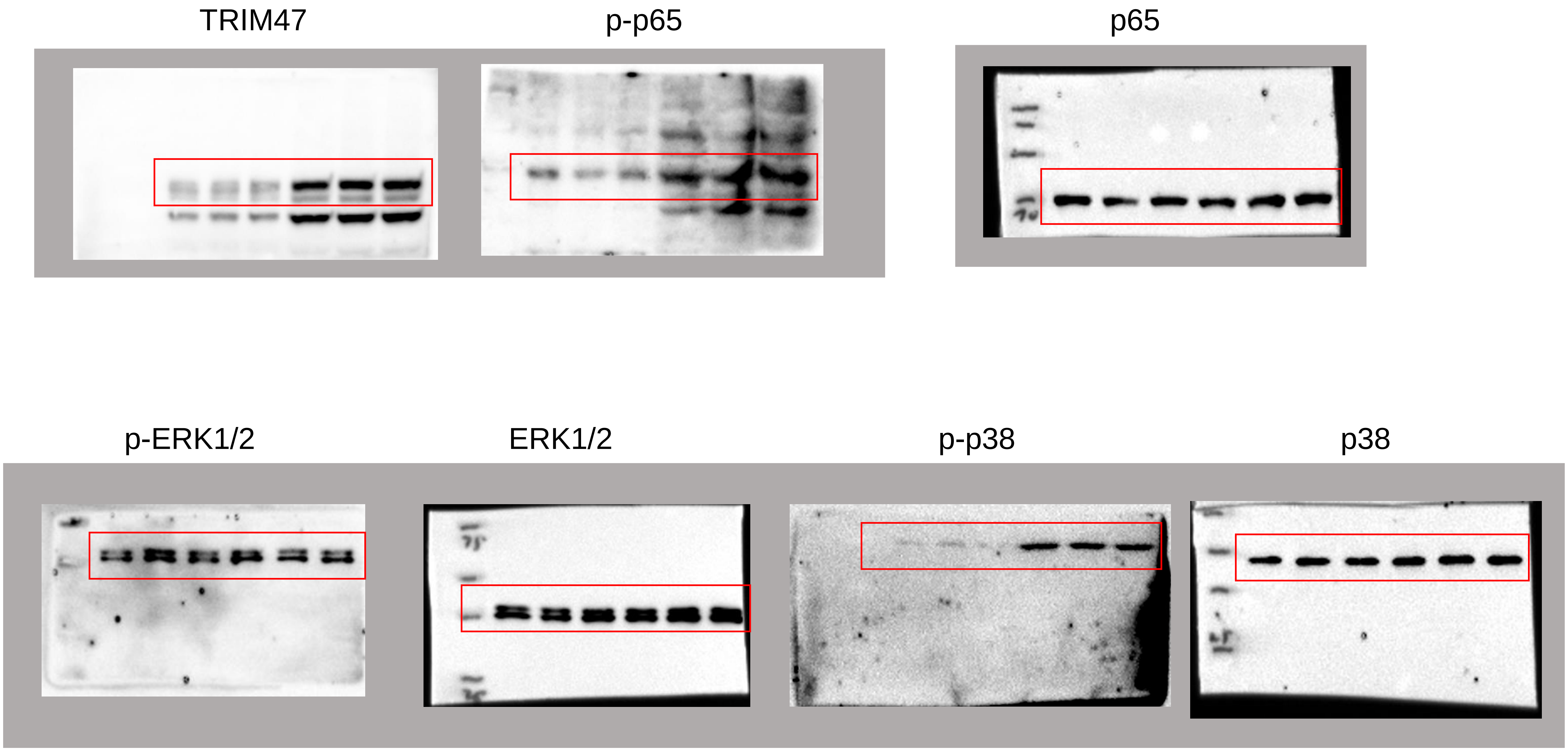

Figure 6C

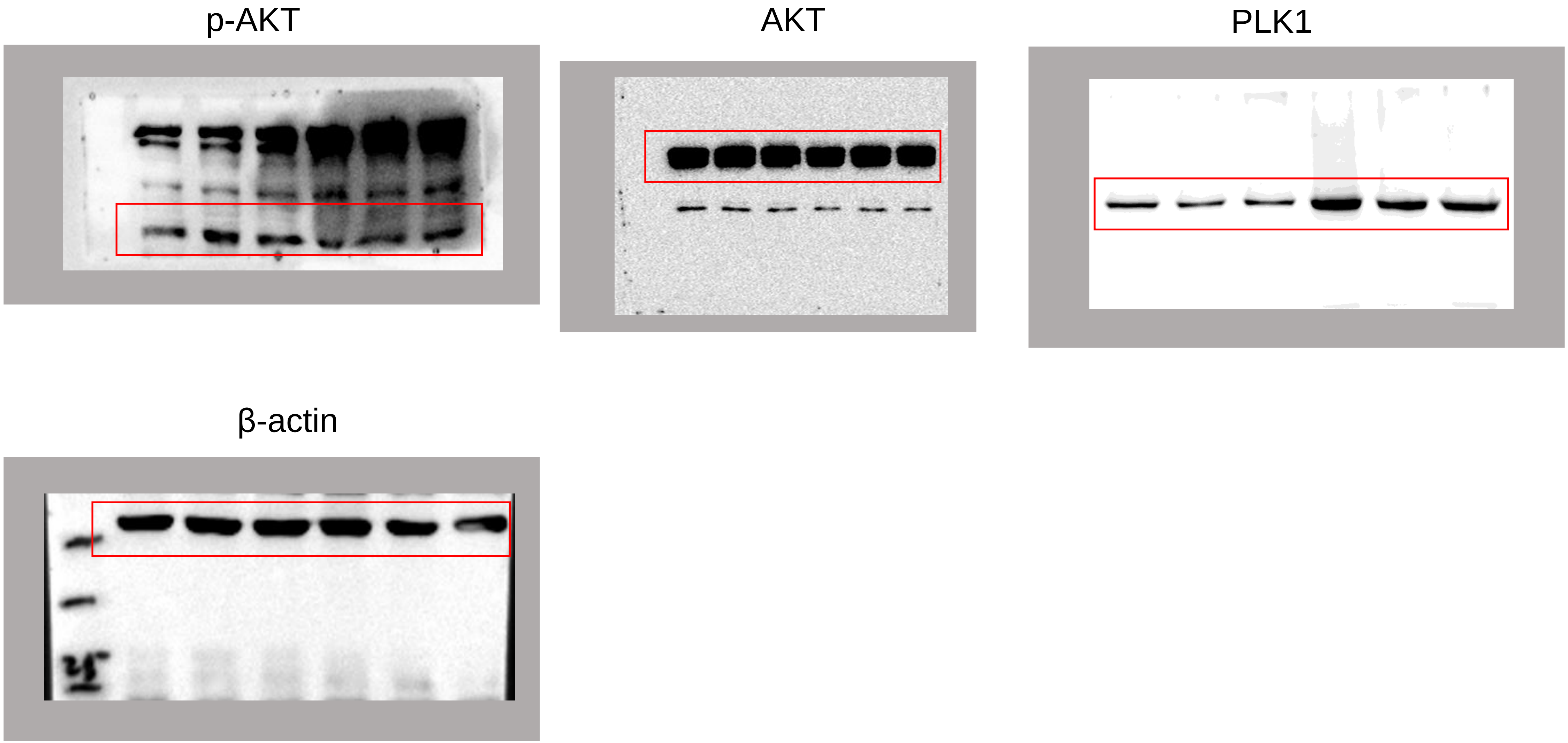

Figure 6D

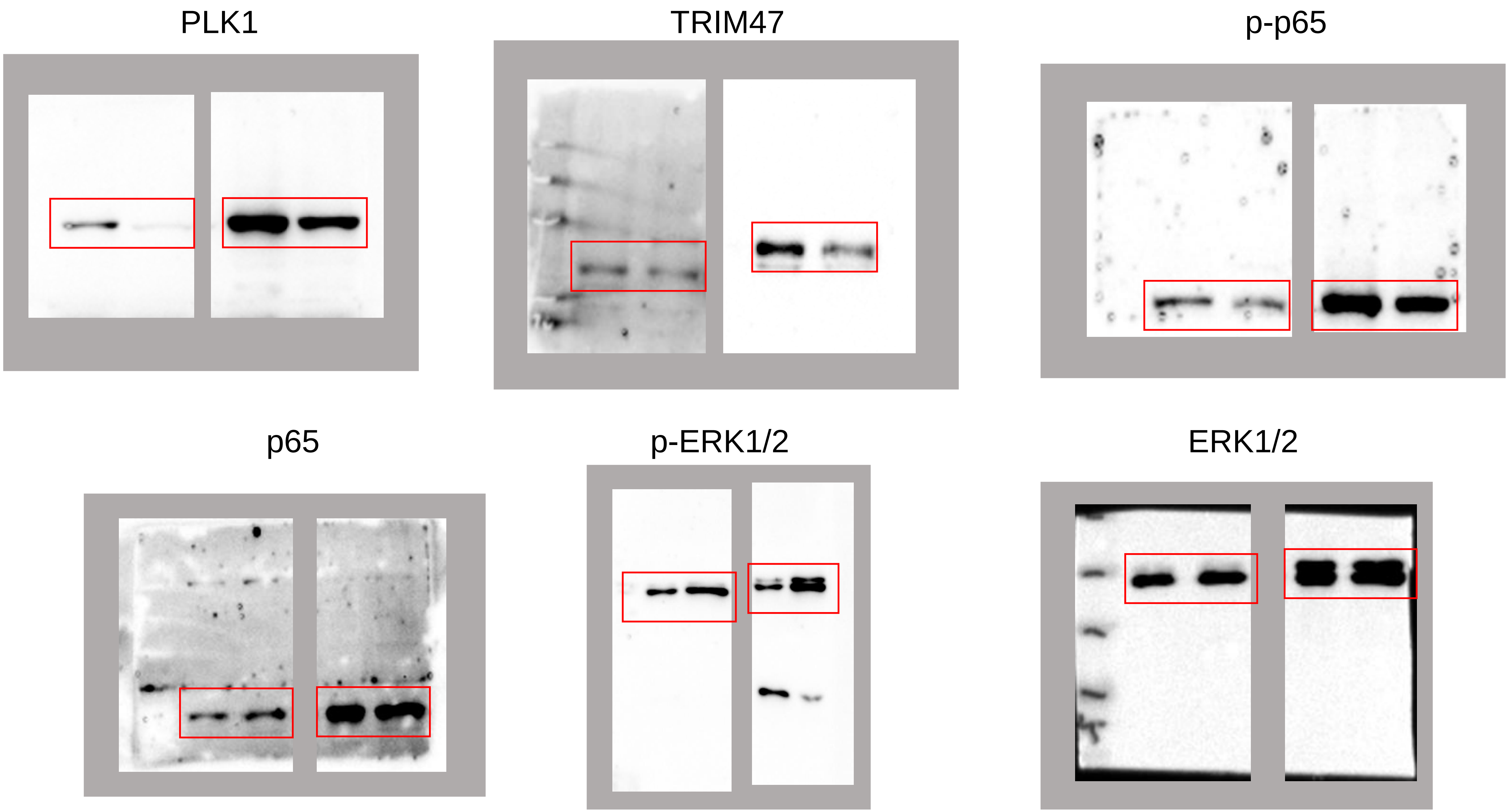

Figure 6D

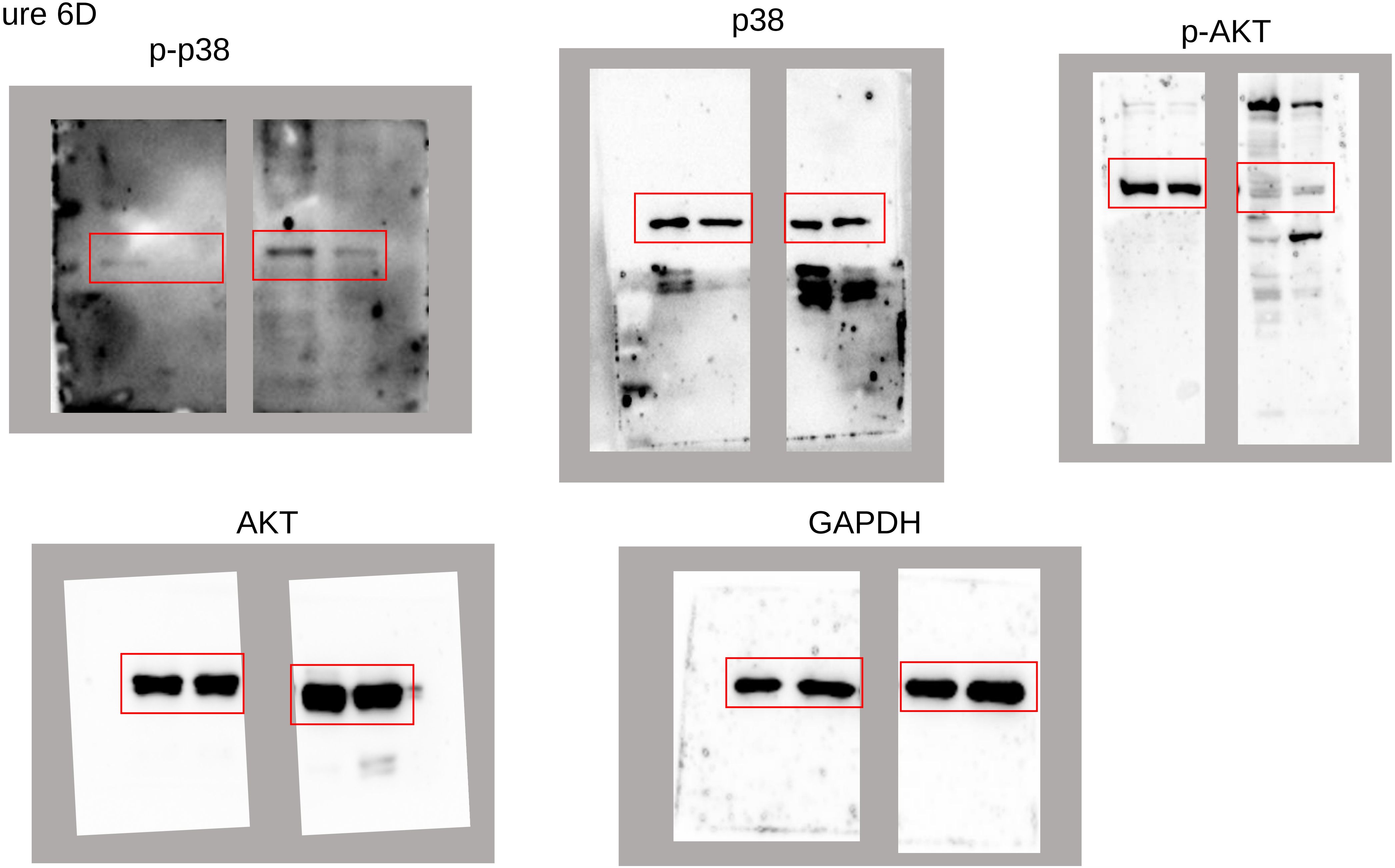

Figure 7A

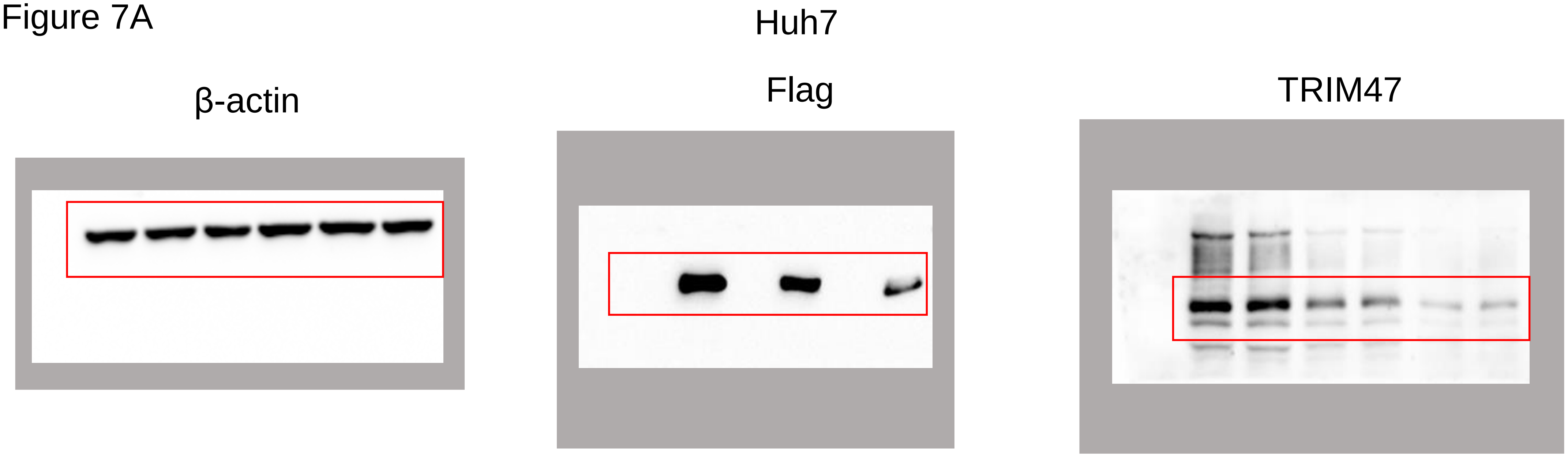

Figure 8F

TRIM47

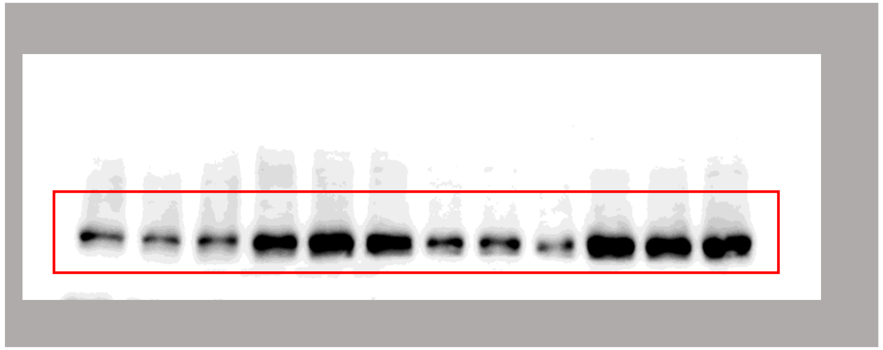

PLK1

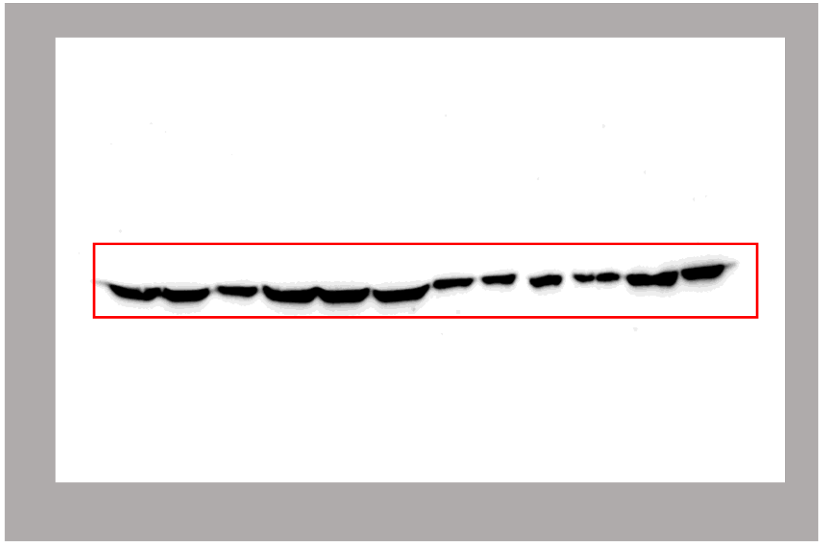

PCNA

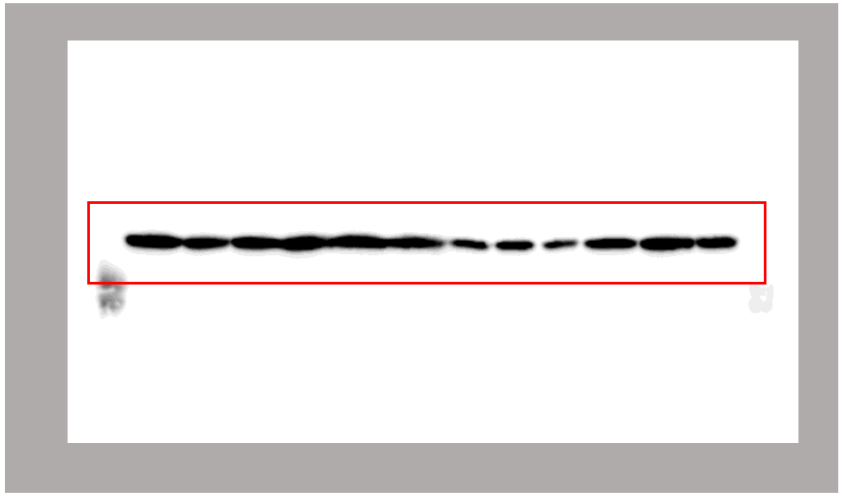

CyclinD1

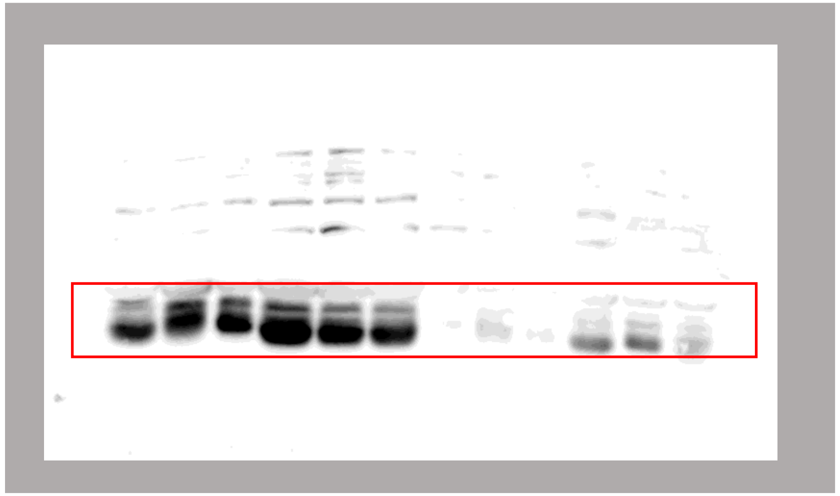

Figure 8E

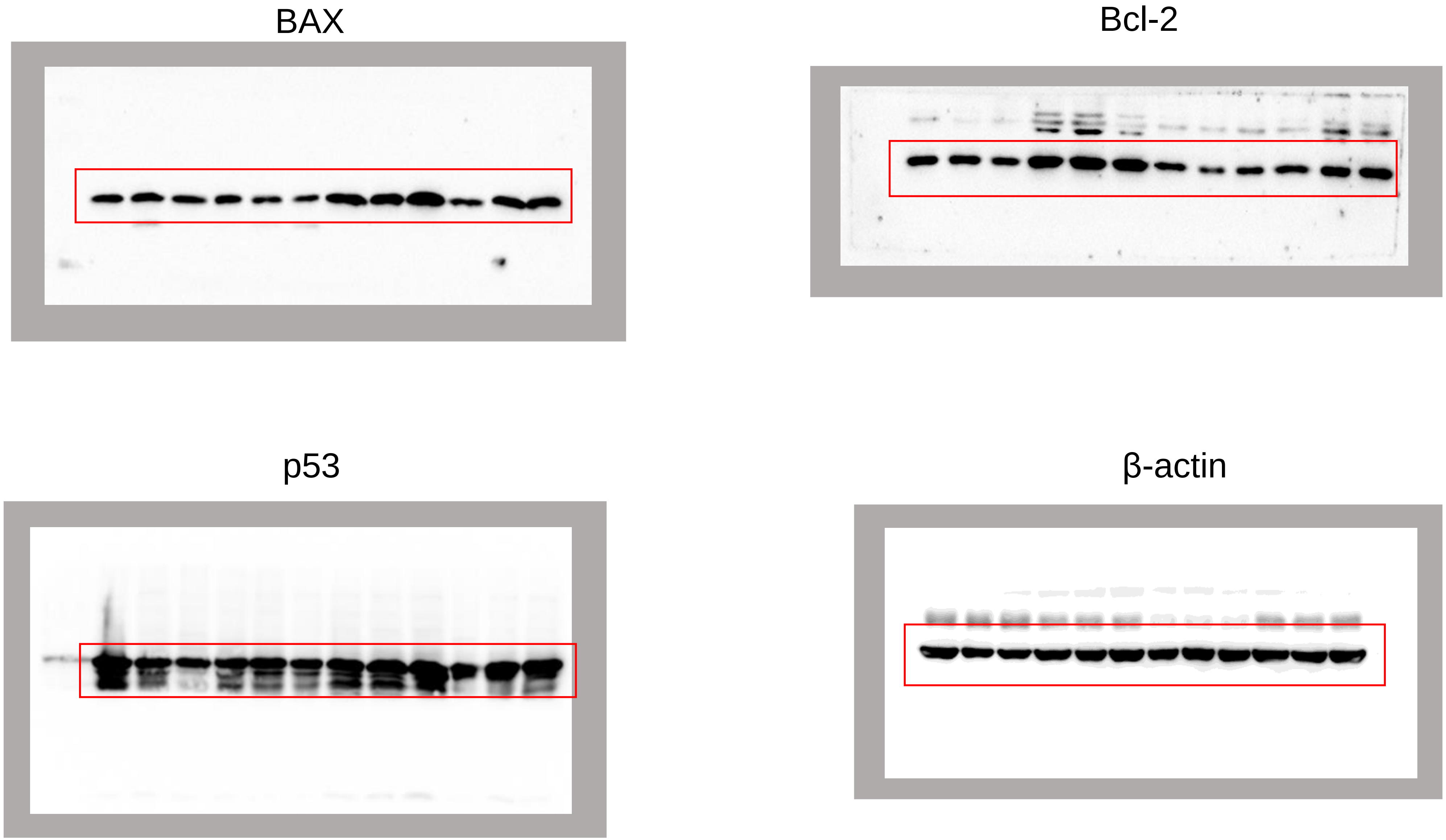

Supplement: Supplementary file 1 — Supplementary Material 1 [file 13402_2025_1130_MOESM1_ESM.pdf]
